# Supplementary material for: Remapping parasite landscapes: Nationwide prevalence, intensity and risk factors of schistosomiasis and soil-transmitted helminthiasis in Rwanda
Source: PLoS Negl Trop Dis. 2025 Aug 25;19(8):e0013328. doi: 10.1371/journal.pntd.0013328 (PMC12377619; doi:10.1371/journal.pntd.0013328)
Supplement: S1 Text — (DOCX) [file pntd.0013328.s001.docx]

**Remapping Parasite Landscapes: Nationwide Prevalence, Intensity and Risk Factors of Schistosomiasis and Soil-Transmitted Helminthiasis in Rwanda**

**S1 Text. Age-Adjusted Prevalence of Soil-Transmitted Helminths and Schistosoma *mansoni***

**Calculation of Age-Adjusted Estimates**

The age-adjusted estimates were calculated to ensure they reflected the underlying population structure, using population data from the Rwanda Population Projection (medium scenario, 2020) and analyzed with the **survey** package in R. The methodology involved several steps:

1. **Population Data Preparation**:
   - Population data by age and sex for 2020 was extracted from the Rwanda Population Projection.
   - The total population was calculated by summing the population across all age groups.
   - The population was grouped into three specific age categories:
     - 1–4 years (pre-SAC)
     - 5–15 years (SAC)
     - 16+ years (Adults).
   - The proportions of the total population in each age category were computed.
   - National weights for each age group were calculated by dividing the national-level population estimate for each age category by the corresponding sample size from the survey.
   - These national weights were applied to calculate the overall prevalence and totals at the national level.
2. **District-Level Population Adjustment**:
   - District-level population data for 2020 was extracted from the Rwanda Population Projection and adjusted using the age-category proportions derived from the national population.
   - The total number of survey participants in each age category was summarized from the study dataset.
   - Sampling weights for each age group were calculated by dividing the district-level population estimate by the corresponding sample size from the survey.
3. **Survey Design**:
   - The **survey** package in R was used to define the survey design.
   - The design incorporated the calculated sampling weights, with sectors as the primary sampling units (PSUs), villages as the secondary sampling units, and districts as strata.
4. **Weighted Estimation of Prevalence**:
   - Using survey design-based methods, the following estimates were derived for each dependent variable:
     - **Means**: Weighted prevalence of binary outcomes, including standard errors and confidence intervals.
     - **Totals**: Weighted total number of individuals affected, including standard errors and confidence intervals.

**Age-Adjusted Prevalence of Soil-Transmitted Helminths and Schistosoma mansoni**

| **Species** | | **Overall** | | **pre-SAC (1 - 4 years)** | | **SAC (5 - 15 years)** | | **Adults (16 and above)** | |
| --- | --- | --- | --- | --- | --- | --- | --- | --- | --- |
|  |  | **Prevalence** | **95% CI** | **Prevalence** | **95% CI** | **Prevalence** | **95% CI** | **Prevalence** | **95% CI** |
| **Soil-Transmitted Helminths (STH)** | |  |  |  |  |  |  |  |  |
|  | Any STH | 42.4 | [40 - 44.8] | 30.2 | [27.5 - 32.9] | 38.8 | [35.7 - 42] | 46.1 | [43.7 - 48.5] |
|  | Ascaris lumbricoides | 26.8 | [24.3 - 29.4] | 24.5 | [21.8 - 27.1] | 30.5 | [27.6 - 33.5] | 25.6 | [23.1 - 28.1] |
|  | Hookworm | 15.3 | [14.1 - 16.5] | 4.3 | [3.7 - 4.9] | 6.1 | [5.3 - 6.9] | 21.3 | [19.5 - 23] |
|  | Trichius trichiura | 11.9 | [9.7 - 14] | 8.4 | [6.6 - 10.2] | 15 | [12.4 - 17.7] | 11 | [8.9 - 13.1] |
| **Schistosoma mansoni** | |  |  |  |  |  |  |  |  |
|  | S.mansoni (KK) | 1.9 | [1.3 - 2.4] | 0.6 | [0.3 - 0.9] | 2.4 | [1.5 - 3.3] | 1.8 | [1.3 - 2.4] |
|  | S.mansoni (CCA - Trace positive) | 23.5 | [22 - 25.1] | 35.6 | [33.7 - 37.6] | 25.6 | [23.8 - 27.4] | 20.5 | [18.8 - 22.2] |
|  | S.mansoni (KK or CCA - Trace positive) | 23.9 | [22.3 - 25.5] | 35.4 | [33.5 - 37.3] | 25.9 | [24.1 - 27.8] | 20.9 | [19.2 - 22.7] |
|  | S.mansoni (CCA - Trace negative) | 9.1 | [8.1 - 10.2] | 15.5 | [14.3 - 16.8] | 10.1 | [8.8 - 11.4] | 7.6 | [6.4 - 8.7] |
|  | S.mansoni (KK or CCA - Trace negative) | 9.7 | [8.6 - 10.9] | 15.6 | [14.4 - 16.9] | 10.7 | [9.2 - 12.1] | 8.3 | [7.1 - 9.5] |
|  | *CCA: Circulating Cathodic Antigen*  *KK: Kato-Katz*  *pre-SAC: preschool-aged children*  *SAC: school-aged children* | | | | | | | | |
