# Supplementary material for: Remapping parasite landscapes: Nationwide prevalence, intensity and risk factors of schistosomiasis and soil-transmitted helminthiasis in Rwanda
Source: PLoS Negl Trop Dis. 2025 Aug 25;19(8):e0013328. doi: 10.1371/journal.pntd.0013328 (PMC12377619; doi:10.1371/journal.pntd.0013328)
Supplement: S4 Table — (DOCX) [file pntd.0013328.s005.docx]

**S4 Table. Summary results of 2008, 2014, and 2020 surveys used for comparison**

| **Condition** | **Survey Year** | **Age group and type of survey** | **Tested** | **Prevalence** |
| --- | --- | --- | --- | --- |
| Any STH | 2008 | 10-16 years (schools) | 8313 | 65.8 |
|  | 2014 | 9-18 years (schools) | 9251 | 45 |
|  | 2020 | Filtered 5 - 15 years (Community) | 6104 | 38.8 |
| Ascaris | 2008 | 10-16 years (schools) | 8313 | 38.6 |
|  | 2014 | 9-18 years (schools) | 9251 | 37 |
|  | 2020 | Filtered 5 - 15 years (Community) | 6098 | 30.5 |
| Hookworm | 2008 | 10-16 years (schools) | 8313 | 31.6 |
|  | 2014 | 9-18 years (schools) | 9251 | 5 |
|  | 2020 | Filtered 5 - 15 years (Community) | 6096 | 6.1 |
| Trichuris | 2008 | 10-16 years (schools) | 8313 | 27 |
|  | 2014 | 9-18 years (schools) | 9251 | 23 |
|  | 2020 | Filtered 5 - 15 years (Community) | 6101 | 15 |
| S. mansoni (CCA - Trace positive) | 2008 | 10-16 years (schools) | 8313 | Not used |
|  | 2014 | 9-18 years (schools) | 9251 | 36.1 |
|  | 2020 | Filtered 5 - 15 years (Community) | 6151 | 25.6 |
| S. mansoni (KK) | 2008 | 10-16 years (schools) | 8313 | 2.7 |
|  | 2014 | 9-18 years (schools) | 9251 | 1.9 |
|  | 2020 | Filtered 5 - 15 years (Community) | 6100 | 2.4 |
