# Supplementary material for: Remapping parasite landscapes: Nationwide prevalence, intensity and risk factors of schistosomiasis and soil-transmitted helminthiasis in Rwanda
Source: PLoS Negl Trop Dis. 2025 Aug 25;19(8):e0013328. doi: 10.1371/journal.pntd.0013328 (PMC12377619; doi:10.1371/journal.pntd.0013328)
Supplement: S1 Data — (DOC) [file pntd.0013328.s006.doc]

## **Registration of participants**

**[Participant details were uploaded in tablets prior to data collection]**

| Date of the visit | *DD-*  *MM-*  *YYYY)* | |___|___|-  |___|___|  |___|___|___|___| | | Team leader’s  Family name  First name  Initials | |___|___|___| | | |
| --- | --- | --- | --- | --- | --- | --- | --- |
| District | | |  | | | Code 2 digits (NISR) | |___|___| |
| Sector | | |  | | | Code 4 digits (NISR) | |___||___|___||___| |
| Cell | | |  | | | Code 6 digits (NISR) | |___||___|___||___||___|___| |
| Village | | |  | | | Code 8 digits (NISR) | |___|___|___|___|___||___|___|___| |

| **Registration of individuals selected for the remapping** | | | | | | | | | | |
| --- | --- | --- | --- | --- | --- | --- | --- | --- | --- | --- |
|  | **Family name and first name** | **Age** | **Sex** | **Code of part.**  **Part. / HH ID** | **Participant participation (Y:Yes; N:No; P:Pending)** | | | | | |
|  |  |  |  |  | **Stool** | **Urine** | **Questionnaire** | **Treated** | | **Referred to HC** |
|  |  |  |  |  |  |  |  | **Alb/Meb** | **PZQ** |  |
|  |  |  |  | |__|__|/|__|___|__| |  |  |  |  |  |  |
|  |  |  |  | |__|__|/|__|___|__| |  |  |  |  |  |  |
|  |  |  |  | |__|__|/|__|___|__| |  |  |  |  |  |  |
|  |  |  |  | |__|__|/|__|___|__| |  |  |  |  |  |  |
|  |  |  |  | |__|__|/|__|___|__| |  |  |  |  |  |  |
|  |  |  |  | |__|__|/|__|___|__| |  |  |  |  |  |  |
|  |  |  |  | |__|__|/|__|___|__| |  |  |  |  |  |  |
|  |  |  |  | |__|__|/|__|___|__| |  |  |  |  |  |  |
|  |  |  |  | |__|__|/|__|___|__| |  |  |  |  |  |  |

| **Individual POC-CCA & Hematuria results** | | | | | |  |
| --- | --- | --- | --- | --- | --- | --- |
| Date of the visit | (*DD-MM-YYYY)* | |___|___|-|___|___|-|___|___|___|___| | District (name) |  | Lab Tech  Initials __________ | |
|  |  |  | Sector (Name) |  |  |  |
|  |  |  | Village (name) |  |  |  |

| **Results** | | |  | | |
| --- | --- | --- | --- | --- | --- |
|  | **Part. / HH ID** | **Results CCA^1^** | | **Hematuria^2^** |  |
|  | |__|__|/|__|__|__ |  | |  |  |
|  | |__|__|/|__|__|__ |  | |  |  |
|  | |__|__|/|__|__|__ |  | |  |  |
|  | |__|__|/|__|__|__ |  | |  |  |
|  | |__|__|/|__|__|__ |  | |  |  |
|  | |__|__|/|__|__|__ |  | |  |  |
|  | |__|__|/|__|__|__ |  | |  |  |
|  | |__|__|/|__|__|__ |  | |  |  |
|  | |__|__|/|__|__|__ |  | |  |  |
|  | |__|__|/|__|__|__ |  | |  |  |
|  | |__|__|/|__|__|__ |  | |  |  |
|  | |__|__|/|__|__|__ |  | |  |  |
|  | |__|__|/|__|__|__ |  | |  |  |
|  | |__|__|/|__|__|__ |  | |  |  |
|  | |__|__|/|__|__|__ |  | |  |  |
|  | |__|__|/|__|__|__ |  | |  |  |
|  | |__|__|/|__|__|__ |  | |  |  |
|  | |__|__|/|__|__|__ |  | |  |  |
|  | |__|__|/|__|__|__ |  | |  |  |
|  | |__|__|/|__|__|__ |  | |  |  |
|  | |__|__|/|__|__|__ |  | |  |  |
|  | |__|__|/|__|__|__ |  | |  |  |
|  | |__|__|/|__|__|__ |  | |  |  |
|  | |__|__|/|__|__|__ |  | |  |  |
|  | |__|__|/|__|__|__ |  | |  |  |
|  | |__|__|/|__|__|__ |  | |  |  |
|  | |__|__|/|__|__|__ |  | |  |  |
|  | |__|__|/|__|__|__ |  | |  |  |
|  | |__|__|/|__|__|__ |  | |  |  |
|  | |__|__|/|__|__|__ |  | |  |  |
|  | |__|__|/|__|__|__ |  | |  |  |
|  | |__|__|/|__|__|__ |  | |  |  |
|  | |__|__|/|__|__|__ |  | |  |  |
|  | |__|__|/|__|__|__ |  | |  |  |
|  | |__|__|/|__|__|__ |  | |  |  |
|  | |__|__|/|__|__|__ |  | |  |  |
|  | |__|__|/|__|__|__ |  | |  |  |
|  | |__|__|/|__|__|__ |  | |  |  |
|  | |__|__|/|__|__|__ |  | |  |  |
|  | |__|__|/|__|__|__ |  | |  |  |
|  | |__|__|/|__|__|__ |  | |  |  |
|  | |__|__|/|__|__|__ |  | |  |  |
|  | |__|__|/|__|__|__ |  | |  |  |
|  | |__|__|/|__|__|__ |  | |  |  |
|  | |__|__|/|__|__|__ |  | |  |  |
|  | |__|__|/|__|__|__ |  | |  |  |
|  | |__|__|/|__|__|__ |  | |  |  |
|  | |__|__|/|__|__|__ |  | |  |  |
|  | |__|__|/|__|__|__ |  | |  |  |
|  | |__|__|/|__|__|__ |  | |  |  |
|  | |__|__|/|__|__|__ |  | |  |  |
|  | |__|__|/|__|__|__ |  | |  |  |
|  | |__|__|/|__|__|__ |  | |  |  |
|  | |__|__|/|__|__|__ |  | |  |  |
|  | |__|__|/|__|__|__ |  | |  |  |
|  | |__|__|/|__|__|__ |  | |  |  |
|  | |__|__|/|__|__|__ |  | |  |  |
|  | |__|__|/|__|__|__ |  | |  |  |
|  | |__|__|/|__|__|__ |  | |  |  |
|  | |__|__|/|__|__|__ |  | |  |  |
|  | |__|__|/|__|__|__ |  | |  |  |
|  | |__|__|/|__|__|__ |  | |  |  |

*^1^* ***Negative****=When no red band in the test area;* ***Invalid****: When no control band even if the test band appears,* ***trace=*** *when red band in the test area is barely visible;* ***1+=*** *when red band in the test area is less intense than control band;* ***2+=*** *when red band in the test area is intense as control band ;* ***3+=*** *when red band in the test area is more intense than control band*

*^2^* ***Haematuria :*** *Is classified trace, 1+, 2+, 3+, 4+. Read color changes on standardized reference on the bottle of urine strips.* ***Write «V» for visible hematuria***

| **Individual Kato-Katz results**  |  | | | | | |  | | --- | --- | --- | --- | --- | --- | --- | | District (Name)Lab TechDate of the visit  Initials _________ | | | | | | (*DD-MM-YYYY)* | | |__|__|-|__|__|-|__|__|__|__| |  |  |  | Sector (Name) |  |  | | Village (Name) |  | |  |  | | | | | | | |  |
| --- | --- | --- | --- | --- | --- | --- | --- | --- | --- | --- | --- | --- | --- | --- | --- | --- | --- | --- | --- | --- | --- | --- | --- | --- | --- | --- | --- | --- | --- | --- | --- | --- |
| | **Results of Kato-Katz** | | | | | | | | | --- | --- | --- | --- | --- | --- | --- | --- | |  | Individual’s Code (2 digits) | Number of eggs counted on KK slide^1^ | | | | | | | Slides | Ankylostoma  **(max. in 20mins)** | S. mansoni | Ascaris | Trichuris | Other parasites | |  | Part|__|__|/  HH|__|__|__| | **S1** | |___|___|___| | |___|___|___| | |___|___|___| | |___|___|___| |  | | **S2** | |___|___|___| | |___|___|___| | |___|___|___| | |___|___|___| |  | |  | Part|__|__|/  HH|__|__|__| | **S1** | |___|___|___| | |___|___|___| | |___|___|___| | |___|___|___| |  | | **S2** | |___|___|___| | |___|___|___| | |___|___|___| | |___|___|___| |  | |  | Part|__|__|/  HH|__|__|__| | **S1** | |___|___|___| | |___|___|___| | |___|___|___| | |___|___|___| |  | | **S2** | |___|___|___| | |___|___|___| | |___|___|___| | |___|___|___| |  | |  | Part|__|__|/  HH|__|__|__| | **S1** | |___|___|___| | |___|___|___| | |___|___|___| | |___|___|___| |  | | **S2** | |___|___|___| | |___|___|___| | |___|___|___| | |___|___|___| |  | |  | Part|__|__|/  HH|__|__|__| | **S1** | |___|___|___| | |___|___|___| | |___|___|___| | |___|___|___| |  | | **S2** | |___|___|___| | |___|___|___| | |___|___|___| | |___|___|___| |  | |  | Part|__|__|/  HH|__|__|__| | **S1** | |___|___|___| | |___|___|___| | |___|___|___| | |___|___|___| |  | | **S2** | |___|___|___| | |___|___|___| | |___|___|___| | |___|___|___| |  | |  | Part|__|__|/  HH|__|__|__| | **S1** | |___|___|___| | |___|___|___| | |___|___|___| | |___|___|___| |  | | **S2** | |___|___|___| | |___|___|___| | |___|___|___| | |___|___|___| |  | |  | Part|__|__|/  HH|__|__|__| | **S1** | |___|___|___| | |___|___|___| | |___|___|___| | |___|___|___| |  | | **S2** | |___|___|___| | |___|___|___| | |___|___|___| | |___|___|___| |  | |  | Part|__|__|/  HH|__|__|__| | **S1** | |___|___|___| | |___|___|___| | |___|___|___| | |___|___|___| |  | | **S2** | |___|___|___| | |___|___|___| | |___|___|___| | |___|___|___| |  | |  | Part|__|__|/  HH|__|__|__| | **S1** | |___|___|___| | |___|___|___| | |___|___|___| | |___|___|___| |  | | **S2** | |___|___|___| | |___|___|___| | |___|___|___| | |___|___|___| |  |   *^1^* *The calculation of number of eggs per gram of stool and average of 2 slides will be computed during statistical analysis* |  |  |  |  |  |  | |
|  |  |
|  |  |

## **Annex 8: Quality control report form for Kato Katz – Form 5**

| Date of visit | (*DD-MM-YYYY)* | |___|___|-|___|___-|___|___|___|___| | Q. Controller’s Signature | __________ |
| --- | --- | --- | --- | --- |
| Quality Controller’s names | |  | | |

| **Results of Kato-Katz Quality Control** | | | | | | | |
| --- | --- | --- | --- | --- | --- | --- | --- |
| No^1^ | Individual’s Code (2 digits) | Number of eggs counted on KK slide | | | | | |
|  |  | Slides | Ankylostoma  **(max. 40mins)** | S. mansoni | Ascaris | Trichuris | Feedback: **C=** Correspondancy  **D =** Discrepancy –then specify : Ex. D/counting or species) |
| 1 | Part|__|__|/HH|__|__|__| |  | |___|__|___| | |___|___|___| | |___|___|___| | |___|___|___| |  |
| 2 | Part|__|__|/HH|__|__|__| |  | |___|___|___| | |___|___|___| | |___|___|___| | |___|___|___| |  |
| 3 | Part|__|__|/HH|__|__|__| |  | |___|___|___| | |___|___|___| | |___|___|___| | |___|___|___| |  |
| 4 | Part|__|__|/HH|__|__|__| |  | |___|___|___| | |___|___|___| | |___|___|___| | |___|___|___| |  |
| 5 | Part|__|__|/HH|__|__|__| |  | |___|___|___| | |___|___|___| | |___|___|___| | |___|___|___| |  |
| 6 | Part|__|__|/HH|__|__|__| |  | |___|___|___| | |___|___|___| | |___|___|___| | |___|___|___| |  |
| 7 | Part|__|__|/HH|__|__|__| |  | |___|___|___| | |___|___|___| | |___|___|___| | |___|___|___| |  |
| 8 | Part|__|__|/HH|__|__|__| |  | |___|___|___| | |___|___|___| | |___|___|___| | |___|___|___| |  |
| 9 | Part|__|__|/HH|__|__|__| |  | |___|___|___| | |___|___|___| | |___|___|___| | |___|___|___| |  |
| 10 | Part|__|__|/HH|__|__|__| |  | |___|___|___| | |___|___|___| | |___|___|___| | |___|___|___| |  |
| 11 | Part|__|__|/HH|__|__|__| |  | |___|___|___| | |___|___|___| | |___|___|___| | |___|___|___| |  |
| 12 | Part|__|__|/HH|__|__|__| |  | |___|___|___| | |___|___|___| | |___|___|___| | |___|___|___| |  |
| 13 | Part|__|__|/HH|__|__|__| |  | |___|___|___| | |___|___|___| | |___|___|___| | |___|___|___| |  |
| 14 | Part|__|__|/HH|__|__|__| |  | |___|___|___| | |___|___|___| | |___|___|___| | |___|___|___| |  |
| 15 | Part|__|__|/HH|__|__|__| |  | |___|___|___| | |___|___|___| | |___|___|___| | |___|___|___| |  |
| 16 | Part|__|__|/HH|__|__|__| |  | |___|___|___| | |___|___|___| | |___|___|___| | |___|___|___| |  |
| 17 | Part|__|__|/HH|__|__|__| |  | |___|___|___| | |___|___|___| | |___|___|___| | |___|___|___| |  |
| 18 | Part|__|__|/HH|__|__|__| |  | |___|___|___| | |___|___|___| | |___|___|___| | |___|___|___| |  |
| 19 | Part|__|__|/HH|__|__|__| |  | |___|___|___| | |___|___|___| | |___|___|___| | |___|___|___| |  |
| 20 | Part|__|__|/HH|__|__|__| |  | |___|___|___| | |___|___|___| | |___|___|___| | |___|___|___| |  |
| 21 | Part|__|__|/HH|__|__|__| |  | |___|___|___| | |___|___|___| | |___|___|___| | |___|___|___| |  |
| 22 | Part|__|__|/HH|__|__|__| |  | |___|___|___| | |___|___|___| | |___|___|___| | |___|___|___| |  |
| 23 | Part|__|__|/HH|__|__|__| |  | |___|___|___| | |___|___|___| | |___|___|___| | |___|___|___| |  |
| 24 | Part|__|__|/HH|__|__|__| |  | |___|___|___| | |___|___|___| | |___|___|___| | |___|___|___| |  |
| Proportion of discrepancy among KK slides re-read^2^ | | | | | | | |__________% |
| Quality of slides prepared (Good, Mild, Bad and explain):  Respect of reading time (Good, Mild, Bad and explain):  General comment if any: | | | | | | | |

**^1^** Do 12 slides (6 of A and 6 of B) in the first 10 villages, when discrepancy was always less than 10%, do QC for only 12 slides (3 of A and 3 of B)

**^2^**If more than 10%, please inform the Study coordinators at NTD Program

## **Quality checking report form for POC-CCA**

| Date of visit | (*DD-MM-YYYY)* | |___|___|-|___|___-|___|___|___|___| | Q. Controller’s Initial | __________ |
| --- | --- | --- | --- | --- |
| Names of QC Reader^1^ | |  | | |

| **Results of POC-CCA Quality Checking** | | | | | | | | |
| --- | --- | --- | --- | --- | --- | --- | --- | --- |
| No^2^ | Individual’s Code (2 digits) | Results of POC-CCA QC | | | | | | |
|  |  |  | QC Reader1 | QC Reader2 | QC Reader3 | Feedback: **C=** Correspondancy ;   **D =** Discrepancy | | Final result for discordant results |
|  |  |  | Initial reader | Initial reader | Initial reader |  | |  |
| 1 | Part|__|__|/HH|__|__|__| |  |  |  |  |  | |  |
| 2 | Part|__|__|/HH|__|__|__| |  |  |  |  |  | |  |
| 3 | Part|__|__|/HH|__|__|__| |  |  |  |  |  | |  |
| 4 | Part|__|__|/HH|__|__|__| |  |  |  |  |  | |  |
| 5 | Part|__|__|/HH|__|__|__| |  |  |  |  |  | |  |
| 6 | Part|__|__|/HH|__|__|__| |  |  |  |  |  | |  |
| 7 | Part|__|__|/HH|__|__|__| |  |  |  |  |  | |  |
| 8 | Part|__|__|/HH|__|__|__| |  |  |  |  |  | |  |
| 9 | Part|__|__|/HH|__|__|__| |  |  |  |  |  | |  |
| 10 | Part|__|__|/HH|__|__|__| |  |  |  |  |  | |  |
| 11 | Part|__|__|/HH|__|__|__| |  |  |  |  |  | |  |
| 12 | Part|__|__|/HH|__|__|__| |  |  |  |  |  | |  |
| 13 | Part|__|__|/HH|__|__|__| |  |  |  |  |  | |  |
| 14 | Part|__|__|/HH|__|__|__| |  |  |  |  |  | |  |
| 15 | Part|__|__|/HH|__|__|__| |  |  |  |  |  | |  |
| 16 | Part|__|__|/HH|__|__|__| |  |  |  |  |  | |  |
| 17 | Part|__|__|/HH|__|__|__| |  |  |  |  |  | |  |
| 18 | Part|__|__|/HH|__|__|__| |  |  |  |  |  | |  |
| Proportion of discrepancy among CCA re-read^3^ | | | | | | | |__________% |  |
| Order at reading bench and samples in order (Good, Mild, Bad and explain):  Respect of reading time (Good, Mild, Bad and explain):  General comment if any: | | | | | | | | |

**^1^** Three (3) experienced laboratory technicians including Q. Controller will re-read immediately the CCA cassettes after the technician have read and record their results on separate form (this template). The QC will compile and compare results of 3 QC readers and the initial reader.

**^2^** 10% of all CCA NEGATIVE results, 10% of all CCA TRACE results, and 10% of all CCA 1+ results

**^3^** Discrepancy of less than 20%: discordant result will be discussed by both readers (#4) to reach an agreement on result to record.

If more than 20%: Q Controller will document it on this form and report to survey coordination (NTD Program)

## **Individual questionnaire for risk factors of SCH and intestinal worms**

| **No** | **QUESTION/VARIABLE** | **RESPONSE** | | | **Skip** |
| --- | --- | --- | --- | --- | --- |
| **Section A Demographic and Socio-economic Information** | | | | | |
| A.1 | Day/Month/Year of Interview | |___|___|.|___|___|.|___|___|___|___| | | |  |
| A.2 | Interviewer | Names:________________________ Sign:___________________________ | | |  |
| A.3 | Team leader | Names:_________________________ | | |  |
| A.4 | Respondent code | District/ Sector/ Cell/ Village/Participant | | |  |
|  |  | **Name of** | Codes –automatic after district’s name is entered | |  |
|  |  | District: | Code 2 digits | \|__\|__\| |  |
|  |  | Sector: | Code 4 digits | \|__\|__\|__\|__\| |  |
|  |  | Cell: | Code 6 digits | \|__\|__\|__\|__\|__\|__\| |  |
|  |  | Village : | Code 8 digits | \|__\|__\|__\|__\|__\|__\|__\|__\| |  |
|  |  | Participant code | | Part\|__\|__\|/HH\|__\|__\|__\| |  |
| A5 | Health Centre attached to | **/_____________________/ (automatic)** | | |  |
|  | Interviewee’s role in household | 1. = Father 2. = Mother 3. = Adult child 4. = Grand father 5. = Grand mother   = Other (specify) | | |  |
| A6 | How old are you?   - In completed years when 5 years+ - In completed months when 1-4 years | /____/____/ | | |  |
| A7 | What is your gender? | 1.Male  2. Female | | |  |
| A8 | Religion | 1 = Catholic church  2 = Pentecost churches  3 = Anglican church  4 = Adventist church  5 = Muslim  6 = Jehovah’s witness  7 = Other, specify | | |  |
| A9 | What is your current marital status | 1. Married.  2. Single/Never  been in union  3. Widowed  4. Divorced  5. Separated | | |  |
| A10 | Are you able to read and write in any language? | 0. No  1. Yes | | |  |
| A11 | Educational attainment) | 1. None  2. Nursery  3. Primary  4. Secondary  5. University/  College  6. Vocational training  7.Literacy classes only | | |  |
| A12 | How long you lived in the current village?   - In completed years when 1 year+ - In completed months when below 1year | /____/____/ | | |  |
| A13 | What is your major occupation currently?  (Whatever you do to earn money or your subsistence)? | 1 = Farmer  2 = Daily labourer (Casual job)  3 = Student  4 = Have no Job (still young or disabled)  5 = Employed (Government or private agencies)  6 = Businessman/women  7 = Housewife  8 = Retired  9 = Other, specify | | |  |
| A14 | Are you head of household? | 0 = No  1 = Yes  (Continue with section B) | | | Sect C |
| **Section B Households Information (Only for Head of Household)** | | | | | |
|  | **Household information on socio-economic, STH & SCH exposure** | | | |  |
| B1 | **Record GPS Location of the HH** | | | |  |
| B2 | How many are you in your households aged from 1 year old? | /____/____/ | | |  |
| B3 | What is your main source of water for domestic use? | 1. Tape 2. Ground water (Iriba) 3. River 4. Lake 5. Pond/ well water (yo mu byobo) 6. Rain water 7. Other Specify: | | | Q. B5 |
| B4 | How much time it takes you to get water from a close safe water source by easy walk? (round trip) | *1=* 0 to 30 min  *2= 31-60 min*  *3= 60 -120 min*  *4= More than 120 min (2 hrs)* | | |  |
| B5 | Do you treat water for drinking in your household? | 0 = No  (Continue with the following Q.)  1 = Yes | | | Q. B7 |
| B6 | If no, why not? | 1. No money to buy products (chemicals, charcoal/wood)  2. No time for water treatment  3. Tape water is already treated by WASAC  4. Ground water (Iriba) is safe  5. Rain water is safe  6. Other Specify: | | |  |
| B7 | If yes, what kind of treatment? | 1. Boiling water 2. Filtration 3. Chemical disinfection (e.g Sur-eau) 4. Storage (Long standing after fetching) 5. Other Specify: __________ | | |  |
| B8 | What means you use to store treated water? | 1. Small Jerican (<6L) tightly covered  2.Closed bucket  3. Open tool  4. Any other tool tightly covered | | |  |
| B9 | Does your household own a latrine? | 0. No  1. Yes | | | Q. B16 |
| B10 | At which age does your child uses the toilet? | /____/____/ | | |  |
| B11 | Before the age of using the toilet, where does he/she defecate and where you dispose feces? | 1. Pot and we put faeces into toilet  2. On the soil and we put faeces into toilet  3. On the soil and faeces are buried  4. Other, specify (disposal) | | |  |
| B12 | Do the household always have water and soap for hand washing at toilet? | 0. No  1. Yes | | |  |
| B13 | Does your household use or ever-used human excreta as fertilizer in farming? (feces or urine) | 0 = No  1 = Yes  (Continue with the following Q.) | | | Q. B16 |
| B14 | Do you treat human excreta before use in farming? | 0 = No  1 = Yes  (Continue with the following Q.) | | | Q. B16 |
| B15 | What do you think is the period of feces treatment (stabilization) before use as fertilizers? | 1.less than 1 month  2. Between 1 and less than 5 months  3. Between 5 and less than 9 months  4. Between 9 and less than 1 year  5. For 1 year and more | | |  |
|  | Household information on SCH Exposure | | | |  |
| B16 | What types of water bodies are close to your household? (***Many options are possible***) | 1. Lake  2. Marshalands for rice plantations  3. Marshalands for other plantations  4. Marsh/ swamp  5. River (Umugezi/Uruzi)  6. Pond/dam  7. Other Specify: | | |  |
|  | How much minutes it takes you from your household to arrive to the close and the far water bodies (lakes, marshlands) by easy walk? | *1= 0-20 min (0-1.6km)*  *2= 21-40 min (1.7-3.2km)*  *3= 41-62 min (3.3-5km)*  *4= 1h03+ (5.1km+)* | | |  |
| B17 | Impact of STH&SCH at Household level | | | |  |
| B18 | Has any of your child ever been absent to school because of intestinal worms related disease? | 0. No  1. Yes | | |  |
| B19 | Have you ever seen or heard any household member passing a worm in stool or vomits? | 0 = No  1 = Yes  (Continue with the following Q.) | | | Q. C1 |
| B20 | If S/he was a child, how old was s/he? (try to recall age in years) | /____/____/ | | |  |
| **Section C Individual Information on practice, MDA and impact of Schistosomiasis and Intestinal Worms** | | | | | |
|  | Individual exposure to SCH | | | |  |
| C1 | Among the following types of water bodies, tell us to which types you have been in contact personally? (***Many options are possible***) | Lake  Marshlands for rice plantations  Marshlands for other plantations  Marsh/ swamp  River (Umugezi/Uruzi)  Pond/dam  Other Specify: | | |  |
| C2 | Among the following activities done in water bodies you mentioned, list all those which put you in contact with water bodies and wetlands such as lakes, river, marshlands, etc.? | Rice cultivation  Other cultivations in marhslands  Expelling birds from rice (Kwamagana)  Fishing  Swimming/ baignade (kwidumbaguza)  Fetching (kuvoma)  Washing clothes  If other, please tell us | | |  |
| C3 | How often do you wear BOOTS when working in rice field? | 0. Never  1. Sometimes  2. Always | | |  |
| C4 | Do you know if any child under-10 years from your household has been taken around lakes or marshlands during cultivation, expelling birds from rice, fetching, etc. | 0 = No  1 = Yes, during cultivation  2 = Yes, during expelling birds  3 = Yes, during fetching  4 = yes, during other activities, specify….. | | |  |
| C5 | Which of the following symptoms did you experience during the last month? | 1= Blood in Urine  2= Blood in stool  3= Itching  4= fever  5= Coughing  6= Abdominal pain  7 = Diarrhoea | | |  |
|  | Information on Individual STH Exposure | | | |  |
| C6 | Is your home have a toilet? | 0. No  1. Yes | | |  |
| C7 | If it happened to you, in which circumstances did you do open defecation among the following circumstances? | 1. Not at all  2. When cultivating where no toilet  3. When going/leaving school  4. When travelling and no toilet around  5. Other specify | | |  |
| C8 | Can you tell us the frequency of all moments where you wash your hands? | 1. When dirty (after farming, etc.)  2. After toilet –even after passing urine?  3. After defecation of baby  4. Before preparing food  5. Before eating food  6. Immediately when coming from travel  7. Other, specify  0. Never  1. Sometimes  2. Always | | |  |
| C9 | How often do you wash fruits before eating them? | 0. Never  1. Sometimes  2. Always | | |  |
|  | How often do drink untreated water? | 0. Never  1. Sometimes  2. Always | | |  |
| C10 | How often do you wear shoes when leaving your household? | 0. Never  1. Sometimes  2. Always | | |  |
|  | Information on MDA Treatment and adverse events | | | |  |
| C11 | Have you been given tablets for bilharzia during the last 12 months? | 0 = No  1 = Yes  (Continue with the following Q.) | | |  |
| C12 | Have you been given tablets for intestinal worms during the last 6 months? | 0 = No  1 = Yes  (Continue with the following Q.) | | |  |
| C13 | Were you given tablets during mass distribution (MCH Week) or when you were sick? | 1. During mass distribution (sites: school, community or at health centre)  2. At Health centre/hospital/clinic when I was sick  3. At pharmacy when I was sick  4. In the community by CHW when I was sick (*Verify with the present CHW*) | | |  |
| C14 | Have you experienced any side effects after taking those medicines during the campaign? | 0 = No  1 = Yes  (Continue with the following Q.) | | | Q. C17 |
| C15 | Among the following side effects, what do you recall well? | 1. Headache  2. Vomiting  3. Skin rash  4. Bloody diarrhoea  5. Diarrhoea without blood  6. Dizziness | | |  |
| C16 | Were you taken to the health facility to treat you because of side effects? | 0. No  1. Yes | | |  |
|  | School status on STH related exposure | | | |  |
| C17 | Are you a pupil/student? | 0 = No  1 = Yes  (Continue with the following Q.) | | | Section D |
| C18 | How often do you have water and soap at school toilet for hand washing after using toilet? | 0. Never  1. Sometimes  2. Always  3. water without soap | | |  |
| C19 | How often do you have treated water for drinking at school? | 0. Never  1. Sometimes  2. Always | | |  |
| C20 | Are school toilet clean enough (no faeces on the floor) to motivate its use? | 0. No  1. Yes | | |  |
| C21 | Have you been absent to school because of intestinal worms related disease? | 0. No  1. Yes | | |  |
| C22 | If you have been feeling bad in your intestines (suspecting intestinal disease), where did you go for treatment? | 0. Nowhere (cured itself)  1. At health facility  2. At pharmacy  3. At traditional healer  4. Other, specify | | |  |
| **Section D Individual information on Knowledge and Attitudes** | | | | | |
|  | **Knowledge and Attitudes towards Bilharzia** | | | |  |
| D1 | Have you ever heard about bilharzia? | 0 = No  1 = Yes  (Continue with the following Q.) | | | Q. D10 |
| D2 | Where did you receive information about bilharzia? | 1.School  2. Community health workers (CHW)  3. Media  4. Health facility (medical professionals)  5. Old parents  6. Churches  7.Other, specify | | |  |
| D3 | Have you ever heard also about its transmission mode, prevention, signs & symptoms, and treatment | 0. No, only the name bilharzia  1. Transmission mode  2. Prevention  3. Signs & symptoms  4. treatment  5. All or some of them (1-4)  6. Other, specify | | | D6 |
| D4 | If yes, what is the frequency of information on bilharzia? | 1. Weekly  2. Monthly  3. Quarterly  4. Yearly  5. Rarely | | |  |
| D5 | How does bilharzia infection transmitted to a person? | 1. Contact with contaminated water of lakes, marshlands by swimming, cultivating, etc.  2. Drink contaminated water with cercaria  3. Don’t know  4. Other, specify | | |  |
| D6 | How human can spread bilharzia? | 1. Open defecation  2. Don’t know  3. Other, specify | | |  |
| D7 | Bilharzia is a disease that can NOT cause severe morbidity or death | 1. Strongly Agree  2. Agree  3. Disagree  4. Strongly disagree | | |  |
| D8 | It is important to periodically screen for bilharzia | 1. Strongly Agree  2. Agree  3. Disagree  4. Strongly disagree | | |  |
| D9 | In our community it is important to take periodically tablets for bilharzia | 1. Strongly Agree  2. Agree  3. Disagree  4. Strongly Disagree | | |  |
| D10 | When I pass blood in stool or feel bad in my intestines I should go to health facility | 1. Strongly Agree  2. Agree  3. Disagree  4. Strongly Disagree | | |  |
|  | **Knowledge and Attitudes towards Intestinal worms** | | | |  |
| D111 | Have you ever heard about intestinal worms? | 0 = No  1 = Yes  (Continue with the following Q.) | | | End interv |
| D12 | Have you ever heard also about its transmission mode, prevention, signs & symptoms, and treatment | 0. No, only the name bilharzia  1. Transmission mode  2. Prevention  3. Signs & symptoms  4. Treatment  5. Other, specify | | | D14 |
| D13 | Where did you receive information about **intestinal worms?** | 1.School  2. Community health workers (CHW)  3. Media  4. Health facility (medical professionals)  5. Old parents  6. Churches  7.Other, specify | | |  |
| D14 | How do **intestinal worms** infection transmitted to a person? | 1. Eating without washing hands  2. Drinking untreated/unsafe water  3. Eating food served on unclean dishes  4. All persons live with intestinal worms in their body  5. I don’t know  6. Other, specify | | |  |
| D15 | How human can spread **intestinal worms**? | 1. Open defecation  2. Not washing hands regularly (after toilet)  3. Not having adequate toilets preventing flies  4. I don’t know  5. Other, specify | | |  |
| D16 | Who reminds you the hygiene practice? (Q for children 10-15 years) | 1. Parents  2. My bother/sister  3. Teacher  4. Health professionals  5. Other, specify | | |  |
| D17 | What are the signs of someone infected by intestinal worms? | 1. Abdominal distension 2. Vomiting/ nausea 3. Loss of appetite 4. Abdominal pain 5. Worms in stool 6. Diarrhoea 7. Body weakness 8. Others, specify | | |  |
| D18 | What do you think is the treatment of intestinal worms? | 1. Albendazole (=Zentel)/ Mebendazole (=Vermox) tablets  2.Some tablets provided at health facility/ pharmacy  3. Traditional medicines  4. Don’t know  5. Other, specify | | |  |
| D19 | Intestinal worms cannot be prevented | 1. Strongly Agree  2. Agree  3. Disagree  4. Strongly Disagree | | |  |
| D20 | You also get intestinal worms when you take sweets foods/ drinks | 1. Strongly Agree  2. Agree  3. Disagree  4. Strongly Disagree | | |  |
| D21 | You can live with intestinal worms without any harm | 1. Strongly Agree  2. Agree  3. Disagree  4. Strongly Disagree | | |  |
| D22 | It doesn’t matter to use untreated human excreta as fertilizer in farming | 1. Strongly Agree  2. Agree  3. Disagree  4. Strongly Disagree | | |  |
| D23 | Herbs for traditional medicines treat well intestinal worms than modern medicine | 1. Strongly Agree  2. Agree  3. Disagree  4. Strongly Disagree | | |  |
| D24 | Herbs used to treat intestinal worms (if any) | 1. I don’t know  2. If you know, specify: …………………… | | |  |

The end!
