# Supplementary material for: Remapping parasite landscapes: Nationwide prevalence, intensity and risk factors of schistosomiasis and soil-transmitted helminthiasis in Rwanda
Source: PLoS Negl Trop Dis. 2025 Aug 25;19(8):e0013328. doi: 10.1371/journal.pntd.0013328 (PMC12377619; doi:10.1371/journal.pntd.0013328)
Supplement: Attachment — Submitted filename: PNTD-D-24-01920_reviewer.pdf [file pntd.0013328.s009.pdf]

PLOS Neglected Tropical Diseases

Remapping parasite landscapes: a nationwide precision mapping of Schistosomiasis and Soil-Transmitted Helminthiasis and associated risk factors in Rwanda

--Manuscript Draft--

**Manuscript Number:**	PNTD-D-24-01920
**Full Title:**	Remapping parasite landscapes: a nationwide precision mapping of Schistosomiasis and Soil-Transmitted Helminthiasis and associated risk factors in Rwanda
**Short Title:**	Prevalence and risk Factors of schistosomiasis and helminthiasis in Rwanda
**Article Type:**	Research Article
**Keywords:**	Schistosomiasis, Soil-Transmitted Helminthiasis, survey, risk factors, parasite prevalence, Rwanda.
**Abstract:**	Background:<br>Soil-transmitted helminthiasis (STH) and schistosomiasis (SCH) remain significant public health challenges in Rwanda, affecting individuals across all age groups. Despite ongoing mass drug administration (MDA) efforts, updated data on prevalence and risk factors are crucial for effective control and elimination strategies. This study reassessed the prevalence of STH and SCH in both children and adults, along with their associated risk factors, to guide control efforts.<br>Methodology:<br>A nationwide survey was conducted across Rwanda's 30 districts, testing 17,360 individuals for STH and 17,342 for Schistosoma mansoni using Kato-Katz (KK) and Point-Of-Care Circulating Cathodic Antigen (POC-CCA) tests. Mixed-effects logistic regression models were used to identify risk factors while accounting for district-level variability.<br>Findings:<br>The overall prevalence of STH was 38.7% (95% CI: 37.9–39.4), with the highest prevalence among adults (46.1%) and the lowest among preschool-aged children (30.2%). District-level prevalence ranged from 8.6% to 85.1%. Prevalence by species included Ascaris lumbricoides (27.0%), Trichuris trichiura (11.6%), and hookworm (10.7%). Moderate-to-heavy intensity (MHI) infections were rare, with A. lumbricoides (8.1%), hookworm (0.1%), and T. trichiura (0.8%). Fourteen districts had villages with an MHI prevalence of ≥2% for at least one STH species among SAC. SCH prevalence ranged from 0% to 38.3% based on KK testing and 0% to 74.6% when including POC-CCA trace positives. S. mansoni MHI was low (0.4%), and five districts had sites with ≥1% MHI for SCH. However, all testing sites had <1% prevalence of heavy-intensity SCH infections.<br>For STH, a higher likelihood of infection was associated with being single (AOR: 1.74), having no education (AOR: 1.56), using human excreta as manure (AOR: 1.43), relying on unimproved water sources (AOR: 1.17), and living near marshlands (AOR: 1.17). A lower likelihood of infection was observed among individuals with higher education (AOR: 0.55), those who were unemployed (AOR: 0.34), self-employed or retired (AOR: 0.53), and students (AOR: 0.54) compared to those in agriculture or farming. Additionally, a lower likelihood was noted among individuals with deep toilet facilities (AOR: 0.78) and those practicing consistent water treatment (AOR: 0.79).<br>For SCH, a higher likelihood of infection was associated with being single (AOR: 1.61), having no education (AOR: 1.41), living near lakes (AOR: 1.76) or rice-farming marshlands (AOR: 1.31), using human excreta as manure whether treated (AOR: 1.32) or untreated (AOR: 1.60), and living more than an hour from a water source (AOR: 1.42).<br>Conclusion:<br>STH and SCH remain significant public health challenges in Rwanda, with certain regions and population groups still exceeding the thresholds for elimination as public health problems. Expanding MDA programs to include adults, improving sanitation and hygiene, ensuring universal access to clean water, and promoting community education on safe practices are essential for achieving sustainable control and elimination of these infections.
**Additional Information:**	

Powered by Editorial Manager® and ProduXion Manager® from Aries Systems Corporation

Question	Response
**Government Employee**<br><br>Are you or any of the contributing authors an employee of the United States government?<br><br>Manuscripts authored by one or more US Government employees are not copyrighted, but are licensed under a CC0 Public Domain Dedication, which allows unlimited distribution and reuse of the article for any lawful purpose. This is a legal requirement for US Government employees.<br><br>This will be typeset if the manuscript is accepted for publication.	No - No authors are employees of the U.S. government.
**Financial Disclosure**<br><br>Enter a financial disclosure statement that describes the sources of funding for the work included in this submission. Review the submission guidelines for detailed requirements. View published research articles from *PLOS Neglected Tropical Diseases* for specific examples.<br><br>This statement is required for submission and **will appear in the published article** if the submission is accepted. Please make sure it is accurate.<br><br>**Funded studies**<br>Enter a statement with the following details:<br>• Initials of the authors who received each award<br>• Grant numbers awarded to each author<br>• The full name of each funder<br>• URL of each funder website<br>• Did the sponsors or funders play any role in the study design, data collection and analysis, decision to publish, or preparation of the manuscript?	Yes

Did you receive funding for this work?	
Please add funding details.<br>as follow-up to "**Financial Disclosure**<br><br>Enter a financial disclosure statement that describes the sources of funding for the work included in this submission. Review the submission guidelines for detailed requirements. View published research articles from *PLOS Neglected Tropical Diseases* for specific examples.<br><br>This statement is required for submission and **will appear in the published article** if the submission is accepted. Please make sure it is accurate.<br><br>**Funded studies**<br>Enter a statement with the following details:<br>• Initials of the authors who received each award<br>• Grant numbers awarded to each author<br>• The full name of each funder<br>• URL of each funder website<br>• Did the sponsors or funders play any role in the study design, data collection and analysis, decision to publish, or preparation of the manuscript?<br><br>Did you receive funding for this work?"	This study was funded by The END Fund (https://end.org/) through a grant to the Ministry of Health, Rwanda Biomedical Centre – The Malaria, Neglected Tropical Diseases, and Other Parasitic Diseases Division (MOPDD). The study was independently implemented by the Rwanda Biomedical Centre – MOPDD. The END Fund provided technical support in designing the study and reviewing the study results and manuscript but had no involvement in the data analysis.
Please select the country of your main research funder (please select carefully as in some cases this is used in fee calculation).<br>as follow-up to "**Financial Disclosure**<br><br>Enter a financial disclosure statement that describes the sources of funding for the work included in this submission. Review the submission guidelines for detailed requirements. View published research articles from *PLOS Neglected Tropical Diseases* for specific examples.<br><br>This statement is required for submission and **will appear in the published article** if the submission is accepted. Please make	UNITED STATES - US

Powered by Editorial Manager® and ProduXion Manager® from Aries Systems Corporation

sure it is accurate.<br><br>**Funded studies**<br>Enter a statement with the following details:<br>• Initials of the authors who received each award<br>• Grant numbers awarded to each author<br>• The full name of each funder<br>• URL of each funder website<br>• Did the sponsors or funders play any role in the study design, data collection and analysis, decision to publish, or preparation of the manuscript?<br><br>Did you receive funding for this work?"	
**Competing Interests**<br><br>On behalf of all authors, disclose any competing interests that could be perceived to bias this work.<br><br>This statement will be typeset if the manuscript is accepted for publication.<br><br>Review the instructions link below and PLOS NTDs' competing interests policy to determine what information must be disclosed at submission.	AO, KP, JT, WL, and ER are affiliated with The END Fund, which funded the study and provided technical support. They did not conduct the data analysis but reviewed the results and contributed to the manuscript. All other authors declare no competing interests.
**Data Availability**<br><br>Provide a **Data Availability Statement** in the box below. This statement should detail where the data used in this submission can be accessed. This statement will be typeset if the manuscript is accepted for publication.<br><br>Before publication, authors are required to make all data underlying their findings fully available, without restriction. Review our PLOS Data Policy page for detailed information on this policy. Instructions for writing your Data Availability statement can be accessed via the Instructions link	All data supporting the findings of this study are included in the article. The dataset is held by the Rwanda Biomedical Center and cannot be publicly shared due to restrictions imposed by the Rwanda Ethics Committee. Participant consent did not permit sharing data beyond the research team, and public disclosure would violate these terms. However, access to the data may be granted upon request, pending approval from the Rwanda Biomedical Center and the Rwanda Ethics Committee. To request access, please contact the Rwanda Biomedical Center at info@rbc.gov.rw, providing a detailed rationale for your request.

below.	

Powered by Editorial Manager® and ProduXion Manager® from Aries Systems Corporation

Full title: Remapping parasite landscapes: a nationwide precision mapping of Schistosomiasis and Soil-Transmitted Helminthiasis and associated risk factors in Rwanda

Short title: Prevalence and risk Factors of schistosomiasis and helminthiasis in Rwanda

Authors:

Ladislas Nshimiyimana[1*], Aimable Mbituyumuremyi[1], Alison Ower[2], Dieudonne Hakizimana[3*], Jean Bosco Mbonigaba[1], Elias Nyandwi[4], Karen Palacio[2], Alphonse Mutabazi[1], Jeanne Uwizeyimana[5], Leonard Uwayezu[6], Michee Kabera[1], Emmanuel Hakizimana[1], Phocas Mazimpaka[1], Emmanuel Ruzindana[7], Eliah Shema[4], Tharcisse Munyaneza[7], Jean Bosco Mucaca[7], Maurice Twahirwa[7], Esperance Umumararungu[7], Joseph Kagabo[8], Richard Habimana[8], Elias Niyituma[1], Tonya Huston[5], Jamie Tallant[2], Warren Lancaster[2], Nadine Rujeni[8], Eugene Ruberanziza[2]

Affiliations

- [1]Division of Malaria and Other Parasitic Diseases , Rwanda Biomedical Centre, Ministry of Health, Kigali, Rwanda
- [2]The END Fund, New York, USA
- [3]Department of Global Health, University of Washington, Seattle, Washington, USA
- [4]Centre for Geographic Information Systems and Remote Sensing (CGIS - UR), College of Sciences and Technology, University of Rwanda, Kigali, Rwanda
- [5]Heart and Sole Africa, Kigali, Rwanda
- [6]Byumba Level Two Teaching Hospital, Gicumbi, Rwanda
- [7]National Reference Laboratory (NRL) Division, Rwanda Biomedical Center, Ministry of Health, Kigali, Rwanda
- [8]School of Health Sciences, College of Medicine and Health Sciences, University of Rwanda, Kigali, Rwanda

***Corresponding author:** **ddhakizimana@gmail.com** **(DH);** **ladi8n@gmail.com** **(LN)**

Key words: Schistosomiasis, Soil-Transmitted Helminthiasis, survey, risk factors, parasite prevalence, Rwanda.

Abstract

Background:
Soil-transmitted helminthiasis (STH) and schistosomiasis (SCH) remain significant public health challenges in Rwanda, affecting individuals across all age groups. Despite ongoing mass drug administration (MDA) efforts, updated data on prevalence and risk factors are crucial for effective control and elimination strategies. This study reassessed the prevalence of STH and SCH in both children and adults, along with their associated risk factors, to guide control efforts.

Methodology:
A nationwide survey was conducted across Rwanda's 30 districts, testing 17,360 individuals for STH and 17,342 for *Schistosoma mansoni* using Kato-Katz (KK) and Point-Of-Care Circulating Cathodic Antigen (POC-CCA) tests. Mixed-effects logistic regression models were used to identify risk factors while accounting for district-level variability.

Findings:
The overall prevalence of STH was 38.7% (95% CI: 37.9–39.4), with the highest prevalence among adults (46.1%) and the lowest among preschool-aged children (30.2%). District-level prevalence ranged from 8.6% to 85.1%. Prevalence by species included *Ascaris lumbricoides* (27.0%), *Trichuris trichiura* (11.6%), and hookworm (10.7%). Moderate-to-heavy intensity (MHI) infections were rare, with *A. lumbricoides* (8.1%), hookworm (0.1%), and *T. trichiura* (0.8%). Fourteen districts had villages with an MHI prevalence of ≥2% for at least one STH species among SAC. SCH prevalence ranged from 0% to 38.3% based on KK testing and 0% to 74.6% when including POC-CCA trace positives. *S. mansoni* MHI was low (0.4%), and five districts had sites with ≥1% MHI for SCH. However, all testing sites had <1% prevalence of heavy-intensity SCH infections.

For STH, a higher likelihood of infection was associated with being single (AOR: 1.74), having no education (AOR: 1.56), using human excreta as manure (AOR: 1.43), relying on unimproved water sources (AOR: 1.17), and living near marshlands (AOR: 1.17). A lower likelihood of infection was observed among individuals with higher education (AOR: 0.55), those who were unemployed (AOR: 0.34), self-employed or retired (AOR: 0.53), and students (AOR: 0.54) compared to those in agriculture or farming. Additionally, a lower likelihood was noted among individuals with deep toilet facilities (AOR: 0.78) and those practicing consistent water treatment (AOR: 0.79).

For SCH, a higher likelihood of infection was associated with being single (AOR: 1.61), having no education (AOR: 1.41), living near lakes (AOR: 1.76) or rice-farming marshlands (AOR: 1.31), using human excreta as manure whether treated (AOR: 1.32) or untreated (AOR: 1.60), and living more than an hour from a water source (AOR: 1.42).

Conclusion:
STH and SCH remain significant public health challenges in Rwanda, with certain regions and population groups still exceeding the thresholds for elimination as public health problems. Expanding MDA programs to include adults, improving sanitation and hygiene, ensuring universal access to clean water, and promoting community education on safe practices are essential for achieving sustainable control and elimination of these infections.

Author summary

Worm infections, like soil-transmitted helminthiasis (STH) and schistosomiasis (SCH), continue to affect people of all ages in Rwanda. Despite efforts to control these infections through regular treatment programs, more up-to-date information on how common they are and what increases the risk of getting them is needed to improve control measures. This study looked at the current situation across people aged 1 year and above in Rwanda.

We carried out a nationwide survey in all 30 districts of Rwanda, testing 17,360 people for worm infections. The results showed that 38.7% of people had intestinal worms, with adults being the most affected (46.1%) and preschool-aged children the least affected (30.2%). The percentage of infected people varied widely between districts, ranging from 8.6% to 85.1%. Among the worms, roundworms were the most common (27.0%), followed by whipworms (11.6%) and hookworms (10.7%). Most infections were mild, with fewer cases of moderate-to-heavy infections, which are of greater concern due to their severe health consequences.

For schistosomiasis, the percentage of people infected ranged from 0% to 38.3% using the standard testing method and up to 74.6% when results from a newer method were included. Moderate-to-heavy intensity infections were rare, with only a small percentage (0.4%) of cases showing higher levels of infection.

We identified certain groups that were more likely to have these infections. For intestinal worms, higher likelihood was seen among single people, those without education, those using treated human excreta as manure, relying on unsafe water sources, and living near marshy areas. On the other hand, people with higher education, clean water, better sanitation, and certain job types were less likely to have infections. For schistosomiasis, the likelihood of infection was higher for those who were single, without education, lived near lakes or rice farms, used human excreta as manure, and lived far from water sources.

Intestinal worms and schistosomiasis remain significant public health challenges in Rwanda. This study highlights the need for continued treatment programs, improved water, sanitation, and hygiene services, and targeted interventions to protect high-risk groups and areas. These measures are essential for reducing the burden of these infections and working toward their elimination.

Introduction

The persistent burden of soil-transmitted helminthiases (STH) and schistosomiasis (SCH) in Rwanda, as in many sub-Saharan African countries, highlights the need for updated data to guide targeted strategies. STH, caused by *Ascaris lumbricoides* (roundworm), *Ancylostoma duodenale/Necator americanus* (hookworm), and *Trichuris trichiura* (whipworm), affects over 1.5 billion people, or 24% of the global population, while SCH impacts over 251 million individuals globally who require preventive treatment, with 90% of cases in Africa [1,2]. In Rwanda, a national survey in 2007–2008 reported an STH prevalence of 65.8% and a SCH prevalence of 2.7%. In response, the Rwandan Neglected Tropical Diseases (NTD) Programme launched mass drug administration (MDA) in 2008, targeting preschool-aged children (pre-SAC), school-aged children (SAC), and selected adults. Albendazole (ALB) or mebendazole (MBZ) was administered biannually, while praziquantel (PZQ) was co-administered in districts with SCH prevalence over 10%. These programs achieved high coverage, with over 90% for ALB (or MBZ) and 75% for PZQ [3–5].

By 2014, an impact assessment demonstrated significant progress. STH prevalence among SAC dropped from 65.8% to 45.2%, though district-level variations remained high, ranging from 2.1% to 89.6%. *Ascariasis* was the most prevalent STH infection (37.2%), followed by *Trichuriasis* (22.8%), while hookworm prevalence was 4.5% [6]. For SCH, *Schistosoma mansoni* prevalence ranged from 1.9% based on the Kato-Katz (KK) technique and it was 7.4% using Point-of-Care Circulating Cathodic Antigen (POC-CCA) testing considering traces as negative results. Additionally, 127 sectors were classified as endemic for SCH. Following the 2014 remapping, two rounds of community- and school-based MDA for STH continued annually in all districts, targeting pre-SAC, SAC , pregnant women, and breastfeeding mothers. An annual single-dose MDA with PZQ continued in the 127 endemic sectors (=sub-districts) [4].

Since the 2014 survey, Rwanda has undergone significant environmental and social changes alongside continuous high-coverage MDA, highlighting the need for a reassessment of STH and SCH. Access to improved water sources increased from 73.8% in 2014 to 80.4% in 2020, while the percentage of households with improved sanitation facilities saw a modest increase from 71.2% to 72.2% [7,8]. At the same time, developments such as expanded agricultural water use, hydroelectric projects, and the creation of artificial lakes may have inadvertently fostered conditions that support SCH transmission.

Evidence suggests that STH infections are more prevalent in adults, particularly in areas where school-based MDA programs have reduced infections in children but sustained transmission [9,10]. Additionally, assessing SCH in adults is crucial for informing treatment strategies [11]. However, MDA programs and evaluations in Rwanda have primarily focused on pre-SAC and SAC, with limited data on adults [5,6,12–14]. Moreover, while the national health management information system captures STH and SCH cases diagnosed at facilities, it excludes individuals with low-intensity or asymptomatic infections who are less likely to seek care, underestimating transmission [15]. Additionally, previous studies that included adults were not nationally representative and often excluded SCH [16]. To address these gaps, comprehensive, updated, and representative data were needed to inform progress toward the World Health Organization's (WHO) elimination targets—<2% prevalence of moderate-to-heavy intensity STH infections

and <1% heavy-intensity SCH infections [17]. In response, this study reassessed the prevalence and risk factors of STH and SCH across the population aged 1 year and above in Rwanda to optimize control strategies, target interventions, and guide efforts toward eliminating these infections as public health problems.

Methods

Ethics statement

This study was conducted in accordance with ethical guidelines and received approval from the Rwanda National Ethics Committee (Approval ~~letter~~ No. 652/RNEC/2020). A written informed consent was obtained from all participants before data collection began. For under-18 years old participants, a consent was signed by parents/guardians as well as an assent form for children aged from 12 years old. Participation was voluntary, and participants could withdraw at any time without consequences. For schoolchildren, permissions were sought from parents and schools to allow them to participate briefly, with transportation arranged to ensure their timely return to school.

Following the interview, those aged two years and above received a single dose of Albendazole (400 mg), while younger children received Mebendazole (500 mg). Samples were tested, and participants were informed promptly once the result was available. If participants needed to leave early, they could consent to have their results delivered via community health workers. Those tested positive for schistosomiasis aged five years and above received a single dose of Praziquantel tablet 600mg while those aged below five years were treated at the health center following the existing treatment guidelines.

Study design

This study conducted a nationwide, community-based cross-sectional survey using a precision mapping approach to assess the prevalence and identify risk factors associated with SCH and STH. Precision mapping focuses on sampling at a finer geographic scale, targeting more schools or villages within smaller administrative units to better capture the variability in schistosomiasis prevalence. Following recommended guidelines, SCH and STH were mapped together, ensuring a comprehensive assessment [17–20].

Study setting

Rwanda, a low-income, landlocked African nation, spans approximately 26.3 square kilometers and with a population of around 12.6 million in 2020, estimated to reach 13.8 million in 2024 resulting in one of the highest population densities in Africa at 501 residents per square kilometer [21,22]. The majority of the population, 72.1%, live in rural areas, with 69% engaged in agriculture. Among households, 63% practice crop farming, and 50% own at least one type of livestock [21]. In 2020, the GDP per capita was estimated at $816 and increased to $1,040 in 2023, with agriculture contributing 27% [23,24].

Rwanda is divided into five provinces: North, East, West, South, and the City of Kigali. Each province is further subdivided into districts, with a total of 30 districts nationwide. These districts are further divided into sectors (416 sectors in total), which are responsible for implementing development programs, delivering services, and promoting governance and social welfare. Sectors are subdivided into cells (2,148 cells), and each cell comprises villages, totaling 14,815 villages [25]. The Rwandan health system follows a traditional hierarchical cascade, structured as community, health center, district hospital, and referral and specialized hospitals [3,4].

Participants

The study participants included pre-SAC (1 to 4 years old), SAC (5 to 15 years old), and adults (16 years and above). Children aged from 1 year old were included because they are targeted in deworming programs, and surveys in similar settings to Rwanda have found that these children are also at risk of SCH infection when their parents or caregivers take them to high-risk areas [26].

Variables

Dependent variables

Schistosomiasis status and intensity

SCH was assessed by examining stool samples for schistosome eggs using the KK technique (duplicate slides), following WHO guidelines [31], and by testing urine samples using the POC-CCA method. For the main analysis, SCH status was determined as positive if an individual tested positive by either KK (presence of at least one S. mansoni egg) or POC-CCA including trace results considered as positive. This approach was selected for programmatic reasons, as the program aims to eliminate SCH by targeting all potentially infected individuals, including those with low-level or asymptomatic infections.

Additionally, results for KK alone were presented with SCH intensity levels, classified by eggs per gram (epg) of feces. Light-intensity was defined as 1–99 epg, moderate-intensity as 100–399 epg, and heavy-intensity as ≥400 epg, as per WHO guidelines [31]. Furthermore, to illustrate variations in SCH prevalence based on test interpretation, we also presented SCH status when trace results for POC-CCA were considered negative and SCH status when an individual tested positive by either KK or POC-CCA but with trace results considered negative.

Any STH Status and Intensity

Any STH status indicates the presence of any STH infection, including Ascaris lumbricoides, hookworm, and Trichuris trichiura. Participants were classified as having an STH infection if their stool samples tested positive for at least one egg of any helminth species using the KK technique with duplicate slides . ~~Specific STH infections were classified as follows: *Ascaris* status indicates the presence of *Ascaris lumbricoides*~~

infection, hookworm status indicates the presence of *hookworm infection*, and *Trichuris* status indicates the presence of *Trichuris trichiura* infection.

The intensity of STH infections was classified based on the number of epg of feces per WHO guideline [31]. For *Ascaris lumbricoides*, light-intensity infections were defined as 1–4,999 epg, moderate-intensity as 5,000–49,999 epg, and heavy-intensity as ≥50,000 epg. For *Trichuris trichiura*, light-intensity was 1–999 epg, moderate-intensity was 1,000–9,999 epg, and heavy-intensity was ≥10,000 epg. For hookworms, light-intensity was 1–1,999 epg, moderate-intensity was 2,000–3,999 epg, and heavy-intensity was ≥4,000 epg [31].

Independent variables

Age was recorded as the participant's actual age and categorized into three groups: pre-SAC (1–4 years), SAC (5–15 years), and Adults (16 years and above). Certain variables were collected only for participants aged 10 years and above, including marital status (single, married, divorced, or widowed), education level (no education, primary, secondary, tertiary), and main occupation, defined as the participant's primary source of income or work.

Other independent variables included the main source of household water, classified as improved (e.g., piped water, wells, and rainwater) or non-improved (all other sources). Additionally, the presence of a toilet facility was recorded, along with its depth if present (<3 meters deep or ≥3 meters deep). We also documented whether households used human feces as manure and whether they treated water prior to drinking, as well as the primary method of treatment among those who reported always treating drinking water. Participants' knowledge about SCH and STH, including transmission, symptoms, and prevention, was also evaluated.

Data sources and measurement

Data collection tools

The survey used a questionnaire to collect information on risk factors, knowledge, attitudes, and practices related to STH and SCH among individuals aged 10 years and above, along with household conditions. It covered demographic details such as age, sex, education, occupation, and household attributes, as well as water sources, sanitation practices, and the use of human waste as manure, focusing on factors that increase infection risk. Additional questions explored exposure to water bodies, potential SCH transmission sources, and hygiene behaviors like handwashing and water treatment. Participants were also asked about infection transmission, symptoms, and their attitudes toward STH and SCH.

The questionnaire was initially developed in English and translated into Kinyarwanda, the local language, by bilingual team members. It was reviewed by other fluent researchers to ensure linguistic accuracy and cultural relevance, then digitized using an Open Data Kit (ODK)-based system for administration on

smartphones. Pre-testing was conducted in two districts to validate its content and ensure consistency in data collection.

Recruitment and data collection procedures

Data collection began with village leaders and community health workers contacting participants from randomly selected households. These local representatives coordinated with health centers to schedule convenient dates and locations for data collection. Upon arrival, participants provided consent and were assigned a card with a unique code. They were then given containers for stool and urine sample collection.

When participants returned with their samples, laboratory technicians verified the samples to ensure that the identifiers matched the participant codes and logged the information into a digital system. If participants were unable to provide a sample immediately, they were encouraged to return it later.

After submitting their samples, participants took part in an interview session. Trained data collectors, who were also nurses, conducted structured interviews in Kinyarwanda using a digital questionnaire. All data collected were uploaded daily to a central database after verification by the team leader for the questionnaire and senior laboratory technicians for the laboratory tests.

Laboratory testing and quality assurance:

Laboratory testing for STH and SCH followed standardized procedures. For STH, including *A. lumbricoides*, *T. trichiura*, and hookworm, stool samples from each participant were analyzed using duplicate Kato-Katz slides. These slides were examined under a light microscope, and egg counts were recorded to determine infection intensity. Due to the fragile nature of hookworm eggs, which can disintegrate after 40-60 minutes, the slides were initially read within 20 minutes to capture hookworm eggs, with a second reading later for other parasites [32]. For SCH, stool samples were analyzed using the KK method, while urine samples were tested using POC-CCA and dipsticks for haematuria. Additional urine tests included centrifugation and microscopic examination when haematuria was detected, to confirm *Schistosoma haematobium*.

To ensure accuracy and quality in laboratory testing, qualified technicians from the National Reference Laboratory (NRL) or health facilities underwent refresher training, supported by bench aids displaying parasite images for verification. During field activities, a senior technician from the NRL or health facilities reviewed 20% of the KK slides daily. Any discrepancies exceeding 10% between the senior technician's readings and those of the field microscopists were required to be reported; however, no significant discrepancies were identified.

For POC-CCA tests, intra-reader reliability checks were implemented. After the initial reading, a quality controller and two additional technicians re-examined 10% of the tests. Results were compared, and discrepancies over 10% but less than 20% were discussed to reach consensus. Larger discrepancies (over 20%) triggered external review and refresher training.

Bias and mitigation measures

To minimize potential biases, the study implemented several strategies. Ten data collection teams, each consisting of lab technicians, nurses, and quality controllers, were supported by local health staff, with field team leaders ensuring standardized procedures across all locations. A five-day training workshop and pre-test in two districts refined logistics, validated tools, and ensured consistency in data collection. Selection bias was addressed by recruiting participants from randomly selected households, coordinated by community leaders to ensure diversity. Reporting bias was minimized through structured interviews conducted by trained nurses using a clear questionnaire, reducing ambiguity and recall errors.

Sample handling bias was prevented by assigning unique codes to participants for accurate tracking of stool and urine samples, with staff verifying identifiers. Standardized procedures including duplicate KK slides and specific timing for hookworm readings, helped reduce measurement bias. Quality assurance measures, such as daily checks by team leaders and senior laboratory technicians and intra-reader reliability checks, ensured consistent testing. Data was recorded by aids of an Open Data Kit (ODK)-based system, with daily uploads to a central server for regular quality checks, review, and cleaning, maintaining data accuracy and completeness.

Study size

The sample size determination followed the WHO protocol for mapping neglected tropical diseases amenable to preventive chemotherapy in the African region, which recommends sampling 50 participants per village and at least five villages in each ecological zone [27]. In this study, villages—the smallest administrative units in Rwanda, each with about 150–200 households—served as the survey areas. The survey covered all 30 districts, with five sectors selected per district, resulting in 150 sectors. Within each sector, two villages were chosen, resulting in a total of 10 villages per district, totaling 300 villages. In each village, 60 participants were selected, aiming for 600 participants per district and a nationwide total of 18,000 participants.

Sampling process

A three-stage sampling design was used to select study villages, focusing on areas likely to support schistosomiasis transmission due to their proximity to water bodies or wetlands.

Stage 1: Determination of eligible villages: In the first stage, villages adjacent to water bodies or wetlands were identified, given the focal transmission patterns of schistosomiasis, which typically occur in such environments. The sampling process was informed by both known and suspected transmission areas, utilizing available epidemiological data as stipulated by the WHO, which recommends purposively considering sub-districts instead of random sampling due to the high focality of schistosomiasis transmission [27]. Geographic Information System (GIS) software and data from the Rwanda Environment Management Authority (REMA) and the Ministry of Environment/Water Department were used to identify 6,165 villages located near significant water bodies or wetlands. Villages were excluded if they were near very small wetlands (less than 1 hectare) or isolated wetlands, assuming that these do not retain sufficient water throughout the year to support transmission. Villages located in wetlands permanently converted to agricultural use, such as tea plantations or other cash crops were also excluded. This determination was made using data from the Ministry of Agriculture and Animal Resources (MINAGRI), the National Agricultural Export Development Board (NAEB), and cross-referenced with satellite imagery from Google Earth [28,29]. Additionally, villages within protected areas, such as national parks, natural forests, and RAMSAR sites, were excluded from the sampling.

Stage 2: Selection of villages: During the second stage, villages at high risk of schistosomiasis transmission were identified based on ecological and epidemiological factors. Data were collected from all eligible villages through structured phone interviews with local officials, such as sector agriculture officers and village leaders, who provided extensive local knowledge. The data collection utilized an adapted WHO form designed to improve the use of available prevalence data and support subdistrict-level planning for schistosomiasis control [30]. The collected data were used to rank the villages by risk level, with the criteria presented in Table 1.

Table 1: Grading criteria for villages to determine their risk for schistosomes

Factor	Condition	Grade
Type of Water Body	Villages adjacent to lakes, water dams, ponds, fishponds, marshes, or wetlands	2
	Villages near rivers or water streams	1
	Villages near torrents or surfaces with non-permanent water	0
Type of Human Activity	Swimming, fishing, rice cultivation, water fetching, irrigation, livestock watering	2
	Cultivation of other crops or vegetation, other activities	1
	No water-related activities	0
Estimated Number of Population Exposed	More than 100 individuals exposed	2
	1-100 individuals exposed	1
	No individuals exposed	0
Proximity of Households to Water Bodies	Households located less than 500 meters from a water body	2
	Households located more than 500 meters from a water body	1
	No adjacent water body	0
	Confirmed cases by survey or routine reporting	1

Factor	Condition	Grade
Confirmed Cases of Schistosomiasis	No confirmed cases	0

Villages that scored high were considered high risk. A total of 3,100 villages were ranked as high risk according to these factors. The high-risk villages were grouped by district and sector. Within each district, five sectors were selected. From each selected sector, two villages were chosen from the pool of high-risk villages, resulting in a total of 10 villages per district and 300 villages nationwide (Fig 1). The selection process was conducted visually, considering the spatial distribution and ecosystem characteristics of the high-risk villages to ensure that the selected sectors and villages were well-distributed across each district, capturing the ecological and geographical variations within the districts.

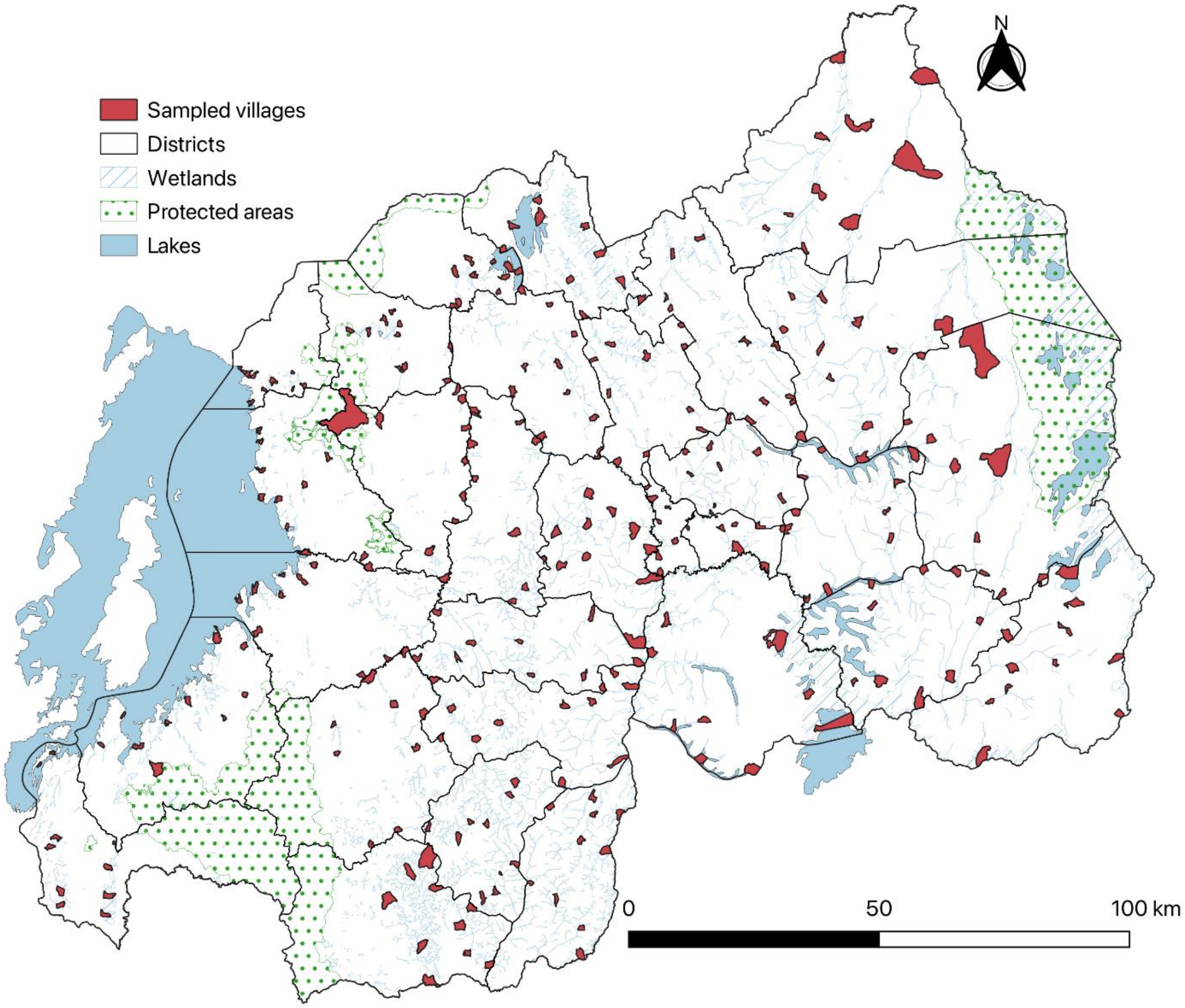

Fig 1: Selected villages across districts highlighted in red.
The map was created by DH using QGIS version 3.40.1-Bratislava. The base layer consists of shapefiles provided by the National Institute of Statistics of Rwanda and publicly available through the Africa GeoPortal, powered by Esri: https://rwanda.africageoportal.com/search?q=rwanda

Stage 3: Selection of households and study participants: For each selected village, health centers conducted a census of all households, recording the name, sex, and age of every individual. Households with members representing all three age groups (1 – 4 years, 5-15 years, and 16+ years) were included in the sampling frame. Systematic sampling was used to select 20 households per village to ensure 60 participants per village. The sampling interval was calculated by dividing the total number of households by the number to be selected. The first household was chosen randomly within this interval, and subsequent households were selected systematically, resulting in a total of 6,000 households across all villages.

Within each of the 20 selected households per village, participants were chosen using simple random sampling to ensure representation across the three age groups. In the first household, one individual from each age group was randomly selected. If there were multiple individuals in an age group, names were written on pieces of paper, mixed, and one name was drawn at random. For subsequent households, participants were selected with consideration to balance the number of males and females per age group in each village [31]. Individuals with mental disabilities preventing them from responding to the survey questions were excluded from the study.

Consideration for STH: It was assumed that STH transmission is relatively homogeneous across a district~~s~~ or a subdistrict~~s.~~ Therefore, the villages and participants selected for SCH were also considered suitable for STH, and no additional sampling was conducted.

Statistical methods

Data were downloaded in Excel format and subsequently exported to R for cleaning and analysis. All data cleaning and analysis were conducted using R version 4.4.1. The dataset was stored on a password-protected computer, ensuring secure access. Only the research team had access to the data. All identifiable information was removed to maintain confidentiality and protect participant privacy.

Descriptive statistics were used to summarize the data. Given the categorical nature of the variables, frequencies and percentages were employed to describe the distribution of the study population, including the prevalence of SCH status, any STH status, and details for each specific STH.

To explore associations between independent variables and outcomes, chi-square tests were conducted to identify variables significantly associated with each outcome at a significance level of $p < 0.05$. Variables showing significant associations, along with potential confounders identified through literature and expert knowledge, were included in mixed-effects logistic regression models with districts as random intercept to determine risk factors for STH and SCH in separate models. To avoid multicollinearity, correlations between variables were tested beforehand, ensuring only non-correlated variables were included. The significance level for the models was set at $p < 0.05$. Adjusted odds ratios (ORs), 95% confidence intervals (CIs), and p-values were reported for each variable included in the final models. Additionally, we conducted a sensitivity analysis by adjusting prevalence for age; the method used to calculate age-adjusted prevalence is detailed in S1 Appendix.

Results

Sociodemographic characteristics

The study included 17,765 participants across five provinces: East (23.2%), Kigali City (10%), North (16.8%), South (26.7%), and West (23.4%). The sample consisted of 53.9% female and 46.1% male participants. Participants were grouped into pre-school-aged children (1–4 years) (31.4%), school-aged children (5–15 years) (34.7%), and adults (16 years and above) (33.9%).

Of the 8,559 participants reporting marital status, 56.7% were married, 36.8% were single, and 6.5% were divorced, separated, or widowed. Among 8,563 respondents, 54% had no formal education, 41.9% had primary education, and 4.1% had secondary or higher education. Most (58.4%) of 8,564 participants who reported their main occupation were engaged in agriculture or farming, while 31.3% were students.

At the household level, 87.4% of the 4,906 surveyed households had a toilet facility with a depth of at least 3 meters, and 57.8% of the 4,841 respondents reported always having soap and water available. In terms of water treatment practices, 45% of respondents indicated they never treated water before drinking, with boiling being the most common method (92.9%) among those who did treat their water. Regarding the use of human excreta as manure, 73.1% of households did not use these, while 22% reported treating them before use.

In relation to proximity to water bodies, 53.3% of households were located near a river, and 64.3% were near marshlands used for activities other than rice farming. Detailed sociodemographic characteristics can be found in Table 2

Table 2: Individuals' and household sociodemographic characteristics

Variables	Categories	n	%
Individual social demographics			
Province (N=17765)	East	4115	23.2
	Kigali City	1768	10
	North	2981	16.8
	South	4750	26.7
	West	4151	23.4
Sex of the participant (N=17765)	Female	9582	53.9
	Male	8183	46.1
Age categories (N=17765)	pre-SAC (1 - 4)	5573	31.4
	SAC (5 - 15)	6166	34.7
	Adults (16 and above)	6026	33.9
Marital status (N=8559)	Single	3151	36.8
	Married	4850	56.7
	Divorced/Separated/Widowed	558	6.5
Education level (N=8563)	No education	4624	54
	Primary	3591	41.9
	Vocational or literacy training	15	0.2
	Secondary and higher	333	3.9

Variables	Categories		n	%
Main occupation (N=8564)	Agriculture/Farmer		5002	58.4
	Employed (formal/informal)		399	4.7
	Self-employed/Retired		135	1.6
	Student		2682	31.3
	Unemployed/Other		346	4
Household level variables				
Has toilet facility (N=4906)	No		65	1.3
	Yes, < 3 meters deep		555	11.3
	Yes, 3+ meters deep		4286	87.4
Always have soap and water (N=4841)*	No		2045	42.2
	Yes		2796	57.8
Main water source (N=4906)	Improved source		1933	39.4
	Non-improved source		2973	60.6
Treat water prior to drinking (N=4906)	Never		2210	45
	Yes, sometimes		1108	22.6
	Yes, always		1588	32.4
	Main water treatment methods *(among those who always treat water)* (N=1588)	Boiling	1475	92.9
		Chemical	34	2.1
		Filtering	62	3.9
		Others	17	1.1
Time to closest water source (round trip) (N=3143)*	On premises or <30 min		1690	53.8
	30 - 60 min		884	28.1
	More than 1 hour		569	18.1
Use human excreta as fertilizer (N=4841) *	No		3539	73.1
	Yes, and treat the fertilizer used		1066	22
	Yes, does not treat fertilizer used		236	4.9
Nearest water bodies (N=4906) *(Participants could have more than one type.)*	Lake		1202	24.5
	River		2616	53.3
	Pond/Dam		1165	23.7
	Marshlands for rice farming		1070	21.8
	Marshlands for other activities		3153	64.3
	Other water bodies		222	4.5
Walk time to closest water bodies (N=4906)	0-20 min		2871	58.5
	21-40 min		1387	28.3
	41+ min		648	13.2

**There were missing values for some participants.*

The prevalence and intensity of STH and *S. mansoni*

Table 3 shows the variation in the prevalence of *STH* and *S. mansoni* across different age groups. The overall prevalence of any STH infection was 38.7% (95% CI: 37.9–39.4), with the highest prevalence observed among adults (46.1%, 95% CI: 44.8–47.3) and the lowest among pre-SAC (30.2%, 95% CI: 29.0–31.5). The prevalence of *A. lumbricoides*, *T. trichiura*, and hookworm was 27.0%, 11.6%, and 10.7%, respectively. SAC exhibited the highest prevalence of *A. lumbricoides* (30.5%, 95% CI: 29.4–31.7) and *T. trichiura* (15.0%, 95% CI: 14.1–15.9), while adults had the highest ~~estimated~~ prevalence of hookworm (21.3%, 95% CI: 20.2–22.3).

The overall prevalence of *S. mansoni* using the KK method was 1.7% (95% CI: 1.5–1.9). When including positives identified by either KK or POC-CCA (with trace results considered positive), the prevalence increased to 27.2% (95% CI: 26.5–27.9), with the highest prevalence observed among pre-SAC (35.4%, 95% CI: 34.1–36.7). No *Schistosoma haematobium* was detected in any tests. Adjusted prevalence estimates for STH and *S. mansoni* are provided in S1 Appendix.

Table 4 presents the intensity of infections with *STH* and *S. mansoni*. Heavy infections were rare, with *A. lumbricoides* highest at 0.5% (95% CI: 0.4–0.7), followed by *Hookworm at 0.1% (95%CI: 0 - 0.1]* and *T. trichiura* at 0.02 % (95% CI: 0.0–0.1), and *S. mansoni* at 0.1% (95% CI: 0.0–0.1). Moderate infections were highest for *A. lumbricoides* at 7.5% (95% CI: 7.1–7.9), while light infections dominated, with *A. lumbricoides* at 18.9% (95% CI: 18.3–19.5), *T. trichiura* at 10.9% (95% CI: 10.4–11.3), and *Hookworm* at 10.6% (95% CI: 10.1–11.1). Combined moderate and heavy infections were highest for *A. lumbricoides* at 8.1% (95% CI: 7.7–8.5).

Table 3: Prevalence of STH and *S. mansoni*

Species	Overall			pre-SAC (1 - 4 years)			SAC (5 - 15 years)			Adults (16 and above)		
	%	95% CI	Tested	%	95% CI	Tested	%	95% CI	Tested	%	95% CI	Tested
**Soil-Transmitted Helminths**												
Any STH	38.7	[37.9 - 39.4]	17360	30.2	[29 - 31.5]	5309	38.8	[37.6 - 40.1]	6104	46.1	[44.8 - 47.3]	5947
A. lumbricoides	27	[26.3 - 27.6]	17348	24.5	[23.3 - 25.6]	5307	30.5	[29.4 - 31.7]	6098	25.6	[24.5 - 26.7]	5943
Hookworm	10.7	[10.3 - 11.2]	17338	4.3	[3.8 - 4.9]	5303	6.1	[5.5 - 6.7]	6096	21.3	[20.2 - 22.3]	5939
T. trichiura	11.6	[11.2 - 12.1]	17352	8.4	[7.7 - 9.2]	5308	15.0	[14.1 - 15.9]	6101	11.0	[10.3 - 11.9]	5943
Schistosoma mansoni												
S. mansoni (KK)	1.7	[1.5 - 1.9]	17342	0.6	[0.4 - 0.9]	5304	2.4	[2 - 2.8]	6100	1.8	[1.5 - 2.2]	5938
S. mansoni (CCA - Trace positive)	27.0	[26.3 - 27.7]	17645	35.6	[34.4 - 36.9]	5485	25.6	[24.5 - 26.7]	6151	20.5	[19.5 - 21.6]	6009
S. mansoni (KK or POC-CCA - Trace positive)	27.2	[26.5 - 27.9]	17719	35.4	[34.1 - 36.7]	5542	25.9	[24.8 - 27]	6159	20.9	[19.9 - 22]	6018
S. mansoni (POC-CCA - Trace negative)	10.9	[10.5 - 11.4]	17645	15.5	[14.6 - 16.5]	5485	10.1	[9.3 - 10.9]	6151	7.6	[6.9 - 8.3]	6009
S. mansoni (KK or POC-CCA - Trace negative)	11.4	[11 - 11.9]	17719	15.6	[14.7 - 16.6]	5542	10.7	[9.9 - 11.5]	6159	8.3	[7.6 - 9]	6018

Table 4: Intensity of STH and *S. mansoni*

Species	Light			Moderate			Heavy			Moderate & Heavy			Mean EPG	95% CI mean EPG
	n	%	95% CI	n	%	95% CI	n	%	95% CI	n	%	95% CI		
A. lumbricoides	3283	18.9	[18.3 - 19.5]	1302	7.5	[7.1 - 7.9]	95	0.5	[0.4 - 0.7]	1397	8.1	[7.7 - 8.5]	6589.1	[6203.1 - 6975.2]
Hookworm	1836	10.6	[10.1 - 11.1]	13	0.1	[0 - 0.1]	11	0.1	[0 - 0.1]	24	0.1	[0.1 - 0.2]	210.4	[167.1 - 253.8]
T. trichiura	1885	10.9	[10.4 - 11.3]	129	0.7	[0.6 - 0.9]	4	0.02	[0 - 0.1]	133	0.8	[0.6 - 0.9]	324.1	[283.8 - 364.5]
S. mansoni - KK	225	1.3	[1.1 - 1.5]	54	0.3	[0.2 - 0.4]	9	0.1	[0 - 0.1]	63	0.4	[0.3 - 0.5]	92.2	[74.3 - 110]

Fig 2 presents the site-specific geographical distribution of prevalence for *A. lumbricoides*, Hookworm, and *T. trichiura* across Rwanda. For A. lumbricoides, higher prevalence clusters are observed in localized areas, particularly in the northern and southern regions. Hookworm prevalence is generally lower across the country, with most areas showing prevalence levels below 10% and only a few regions reaching 20–29.9%. T. trichiura exhibits a similar pattern to A. lumbricoides, with high prevalence areas (≥50%) concentrated in the northern regions, although many regions have prevalence levels below 10%. Additionally, regarding progress toward the elimination of STH as a public health problem, a detailed analysis showed that in 2020, fourteen districts had villages with an MHI prevalence of ≥2% for at least one STH species among SAC.

Fig 3 shows the geographical distribution of S. mansoni prevalence. Using the KK method, most sites had low prevalence levels (<10%), with a few showing higher prevalence (10–49.9%) and none exceeding 50%. In contrast, the combined diagnostic approach (KK or POC-CCA) revealed significantly higher prevalence, with many sites reporting levels of 10–49.9% and several exceeding 50% across the country. Additionally, ~~Additionally,~~ regarding progress toward the elimination of SCH as a public health problem, a detailed analysis showed that in 2020, five districts had sites with ≥1% MHI for SCH. However, all testing sites had <1% prevalence of heavy-intensity SCH infections by KK.

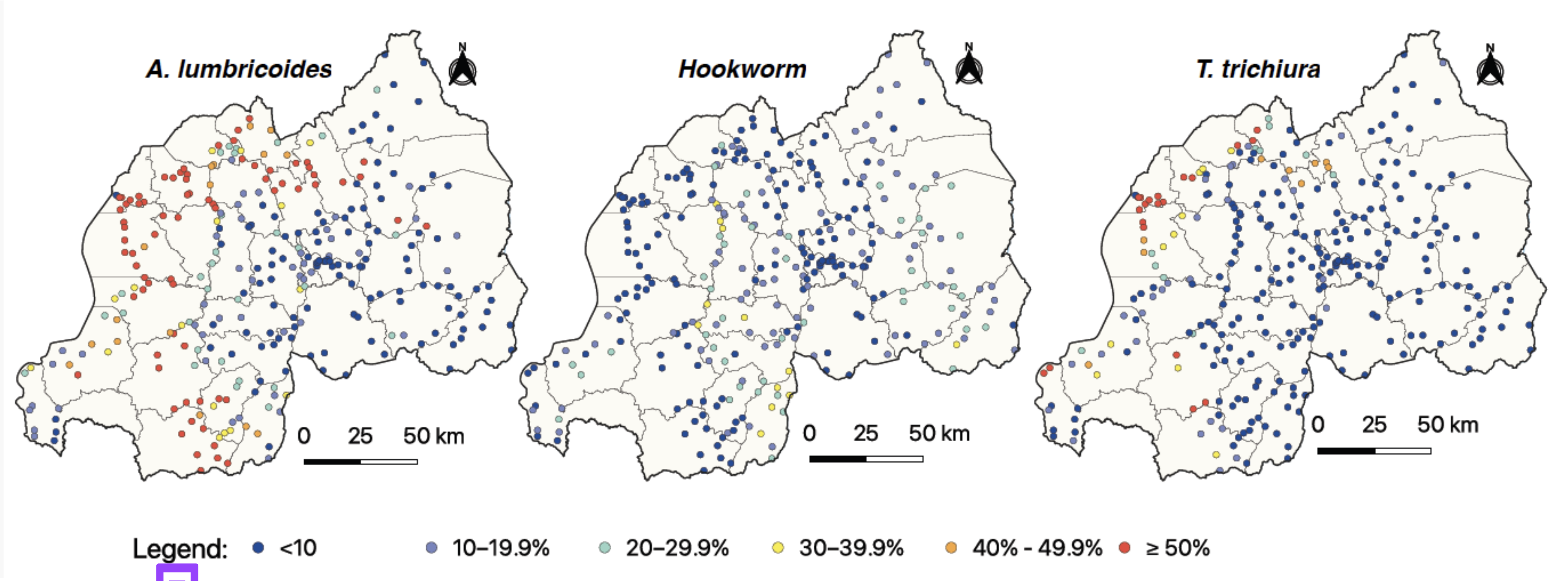

Fig 2: Site-specific geographical distribution of STH prevalence across Rwanda.
The map was created by DH using QGIS version 3.40.1-Bratislava. The base layer consists of shapefiles provided by the National Institute of Statistics of Rwanda and publicly available through the Africa GeoPortal, powered by Esri: https://rwanda.africageoportal.com/search?q=rwanda

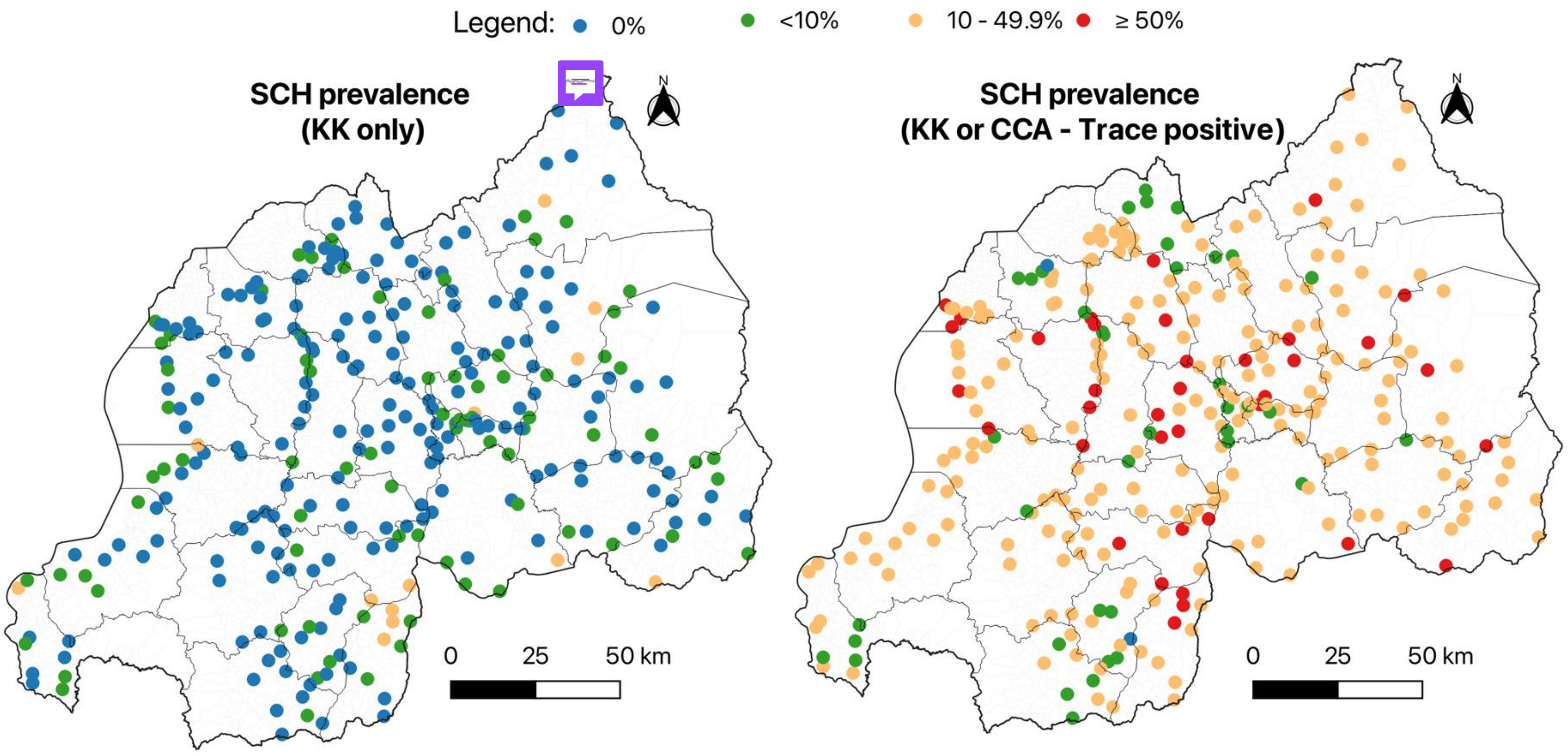

Fig 3: Geographical distribution of S. mansoni prevalence in Rwanda (KK and POC-CCA, traces considered as positive)
The map was created by DH using QGIS version 3.40.1-Bratislava. The base layer consists of shapefiles provided by the National Institute of Statistics of Rwanda and publicly available through the Africa GeoPortal, powered by Esri: [*https://rwanda.africageoportal.com/search?q=rwanda*](https://rwanda.africageoportal.com/search?q=rwanda)

Fig 4 highlights the overall prevalence of STH by district. Prevalence varied significantly across districts, with seven districts reporting a prevalence of any STH ≥50%. The highest prevalence was observed in Rubavu (85.1%), followed by Rutsiro (71.0%) and Nyabihu (69.5%). The lowest prevalence of any STH was reported in Kicukiro (8.6%) and Gasabo (15.3%), both located in Kigali City. Detailed district-level prevalence and intensity data for each species are provided in S2 Appendix.

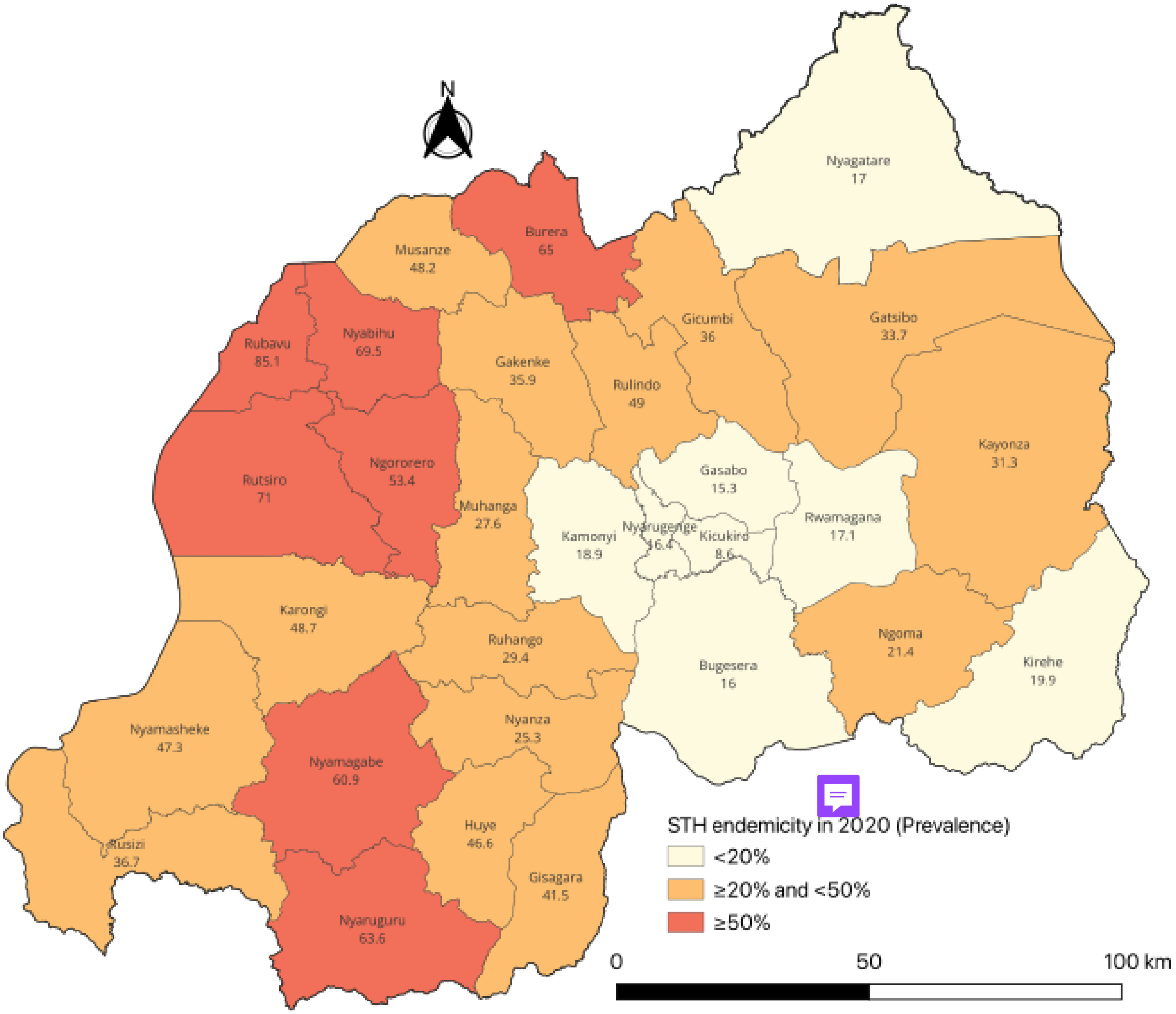

Fig 4: Prevalence of any STH by district for all populations combined

Numbers on the map represent district-level prevalence for any STH. The map was created by DH using QGIS version 3.40.1-Bratislava. The base layer consists of shapefiles provided by the National Institute of Statistics of Rwanda and publicly available through the Africa GeoPortal, powered by Esri: [*https://rwanda.africageoportal.com/search?q=rwanda*](https://rwanda.africageoportal.com/search?q=rwanda)

Prevalence of any STH and *S. mansoni* by individual and household sociodemographic characteristics

Table 5 presents the prevalence of STH and *S. mansoni* (positive on either KK or POC-CCA) by individual and household sociodemographic characteristics. The prevalence of any STH varied by province, with higher rates in the West (58.5%) and lower rates in Kigali City (13.5%) ($p < 0.001$). Adults had a higher STH prevalence (46.1%) compared to pre-SAC at 30.2% and SAC at 38.8% ($p < 0.001$). The prevalence was also higher among divorced, separated, or widowed individuals (51.8%, $p < 0.001$). Individuals aged 10 years and above without formal education had the highest prevalence of STH (50.1%, $p < 0.001$). Individuals engaged in agriculture/farming had higher rates of infection (48.4%), as well as those who reported being employed (formal/informal) (48.3%), compared to other occupational groups ($p < 0.001$).

At the household level, STH prevalence was higher in homes lacking proper toilet facilities (61.9%) or with toilets less than 3 meters deep (62%) ($p < 0.001$), as well as in households where soap and water were not consistently available (51.5%, $p < 0.001$). Additionally, households relying on non-improved water sources or not treating water prior to drinking exhibited higher STH prevalence (50.6% and 51.5%, respectively, $p < 0.001$).

For *S. mansoni*, in addition to higher prevalence among pre-SAC (35.4%) and lower among adults (20.9%) ($p < 0.001$), differences were also seen by sex, with males showing higher prevalence (28.3%) compared to females (26.2%) ($p = 0.0017$). Geographical factors played a role; prevalence was higher among those living near lakes (25.4%, $p < 0.001$) and near rivers (18.6%, $p = 0.0315$). Additionally, proximity to marshlands used for rice farming was associated with a higher prevalence (22.5%, $p = 0.0132$).

Details on prevalences and intensity of Hookworm, *A. lumbricoides*, *T. trichiura*, and *S. mansoni* (KK, POC-CCA Trace Positive/Negative) by sociodemographic characteristics are presented in S3 Appendix.

Table 5: Prevalence of any STH and any *S. mansoni* (positive on either KK or POC-CCA) by individual and household sociodemographic characteristics

Variables	Categories	Any STH			*S. mansoni* (KK or POC-CCA - Trace positive)		
		n (%)	Total (N)	P value	n (%)	Total (N)	P value
**Social demographics**							
Province	East	908 (22.3)	4068	<0.001	1227 (29.9)	4109	<0.001
	Kigali City	223 (13.5)	1649		520 (29.6)	1757	
	North	1375 (46.9)	2933		748 (25.1)	2976	
	South	1826 (39.3)	4645		1195 (25.2)	4733	
	West	2380 (58.5)	4065		1129 (27.2)	4144	
Sex of the participant	Female	3655 (39.1)	9352	0.2265	2505 (26.2)	9553	0.0017
	Male	3057 (38.2)	8008		2314 (28.3)	8166	
Age categories	Adults (16+)	2739 (46.1)	5947	<0.001	1260 (20.9)	6018	<0.001
	pre-SAC (1 - 4)	1603 (30.2)	5309		1962 (35.4)	5542	
	SAC (5 - 15)	2370 (38.8)	6104		1597 (25.9)	6159	
Marital status	Single	1280 (41)	3122	<0.001	824 (26.2)	3143	<0.001
	Married	2164 (45.2)	4790		977 (20.2)	4847	
	Divorced/Separated/Widowed	284 (51.8)	548		108 (19.4)	557	
Education level	No education	2300 (50.1)	4589	<0.001	1068 (23.1)	4619	0.3151
	Primary	1358 (38.4)	3538		767 (21.4)	3585	
	Vocational or Literacy training	5 (33.3)	15		3 (20)	15	
	Secondary and Higher	67 (20.8)	322		73 (22)	332	
Main occupation	Agriculture/Farmer	2398 (48.4)	4959	<0.001	1019 (20.4)	4999	<0.001
	Employed (formal/informal)	188 (48.3)	389		86 (21.7)	397	
	Self Employed/Retired	30 (23.6)	127		38 (28.1)	135	
	Student	1044 (39.3)	2659		695 (26)	2677	
	Unemployed/Other	70 (21.1)	331		73 (21.2)	344	
Household level variables							
Has toilet facility	No	39 (61.9)	63	<0.001	15 (23.1)	65	0.7708
	Yes, < 3 meters deep	342 (62)	552		107 (19.3)	554	
	Yes, 3+ meters deep	1894 (44.7)	4234		846 (19.8)	4281	
Always have soap and water	No	1046 (51.5)	2033	<0.001	426 (20.9)	2042	0.0921
	Yes	1190 (43.2)	2753		527 (18.9)	2793	
Main water source	Improved source	781 (41.2)	1897	<0.001	384 (19.9)	1929	0.8587
	Non-improved source	1494 (50.6)	2952		584 (19.7)	2971	
Water treatment	Never	1129 (51.5)	2192	<0.001	426 (19.3)	2208	0.6443
	Sometimes	527 (47.7)	1104		229 (20.7)	1108	
	Yes, always	619 (39.9)	1553		313 (19.8)	1584	

Variables	Categories		Any STH			S. *mansoni* (KK or POC-CCA - Trace positive)		
			n (%)	Total (N)	P value	n (%)	Total (N)	P value
prior to drinking	Water treatment method (among those who always treat water)	Boiling	584 (40.4)	1444	0.3334	296 (20.1)	1471	0.6165
		Chemical	9 (27.3)	33		5 (14.7)	34	
		Filtering,	21 (34.4)	61		9 (14.5)	62	
		Others	5 (33.3)	15		3 (17.6)	17	
Time to closest water source (round trip)	30 - 60 min		448 (51.1)	876	0.0663	162 (18.3)	884	0.3679
	More than 1 hour		310 (55.1)	563		112 (19.7)	568	
	On premises or <30 min		830 (49.4)	1680		349 (20.7)	1689	
Use human excreta as fertilizer and treat it	No		1516 (43.5)	3486	<0.001	662 (18.7)	3533	0.0083
	Yes, & treat the fertilizer used		597 (56.1)	1064		231 (21.7)	1066	
	Yes, does not treat fertilizer		123 (52.1)	236		60 (25.4)	236	
Nearest water bodies (N=4906) (Participants could have more than one type.)	Lake	No	1709 (46.8)	3652	0.7944	663 (17.9)	3699	<0.001
		Yes	566 (47.3)	1197		305 (25.4)	1201	
	River	No	922 (40.9)	2253	<0.001	482 (21.1)	2286	0.0315
		Yes	1353 (52.1)	2596		486 (18.6)	2614	
	Pond/Dam	No	1729 (46.8)	3697	0.7344	716 (19.2)	3735	0.0719
		Yes	546 (47.4)	1152		252 (21.6)	1165	
	Marshlands for rice farming	No	1809 (47.8)	3788	0.0294	728 (19)	3832	0.0132
		Yes	466 (43.9)	1061		240 (22.5)	1068	
	Marshlands for other	No	765 (44.1)	1733	0.0042	378 (21.6)	1750	0.0173
		Yes	1510 (48.5)	3116		590 (18.7)	3150	
	Other water bodies	No	2181 (47.1)	4631	0.2800	931 (19.9)	4679	0.2869
		Yes	94 (43.1)	218		37 (16.7)	221	
Walk time to closest water bodies	0-20 min		1335 (47)	2841	0.2444	562 (19.6)	2868	0.9454
	21-40 min		656 (48.1)	1364		277 (20)	1385	
	41+ min		284 (44.1)	644		129 (19.9)	647	

Change in prevalence of STH and *S. mansoni* from 2008 to 2020

The Fig 5 shows changes in infection prevalence across surveys from 2008, 2014, and 2020. The prevalence of any STH declined significantly by 20.8% points from 2008 to 2014 (95% CI: -22.2, -19.4) and by 6.2 points from 2014 to 2020 (95% CI: -7.8, -4.6). *A. lumbricoides* prevalence fell by 1.6 points between 2008 and 2014 (95% CI: -3.0, -0.2) and by 6.5 points from 2014 to 2020 (95% CI: -8.0, -5.0). Hookworm dropped sharply by 26.6 points from 2008 to 2014 (95% CI: -27.7, -25.5) but slightly increased by 1.1 points from 2014 to 2020, *T. trichiura* prevalence declined by 4 points from 2008 to 2014 (95% CI: -5.3, -2.7) and by 8 points from 2014 to 2020 (95% CI: -9.2, -6.8). *S. mansoni* prevalence using the CCA method (trace positive) dropped by 10.5 points from 2014 to 2020 (95% CI: -12.0, -9.0). Details of the estimates from previous surveys for each STH and SCH are available in S4 Appendix**.**

Fig 6 shows the spatial distribution of STH endemicity in Rwanda in 2008, 2014, and 2020 among SAC. In 2008, 23 out of 30 districts (76.7%) had high STH prevalence (≥50%), with only 2 districts (6.7%) showing prevalence <20%. By 2014, high-prevalence districts decreased to 12 (40%). In 2020, high-prevalence districts further dropped to 10 (33.3%). Additionally, regarding the progress toward elimination as public health problem, Fourteen of 30 districts had an MHI prevalence of ≥2% for at least one STH species among SAC.

However, some districts experienced an increase in prevalence. Two districts (Kayonza and Ruhango), which had prevalence <20% in earlier years, shifted to the category of prevalence between ≥20% and <50% by 2020. Additionally, one district (Rulindo) showed an increase, shifting from prevalence between ≥20% and <50% to ≥50% in 2020 (Fig 6).

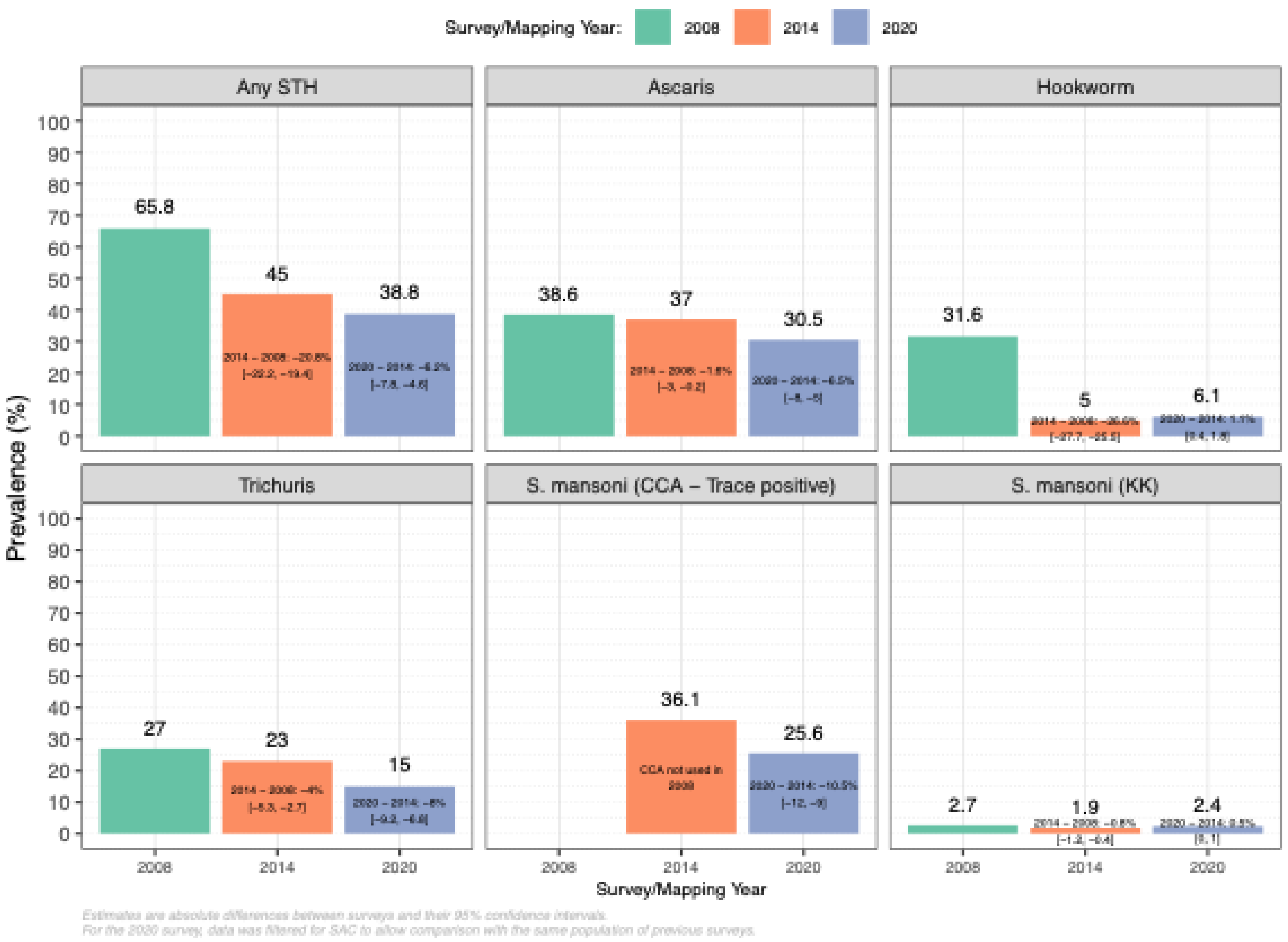

Fig 5: Comparison of SCH and STH survey prevalence among SAC across mapping years

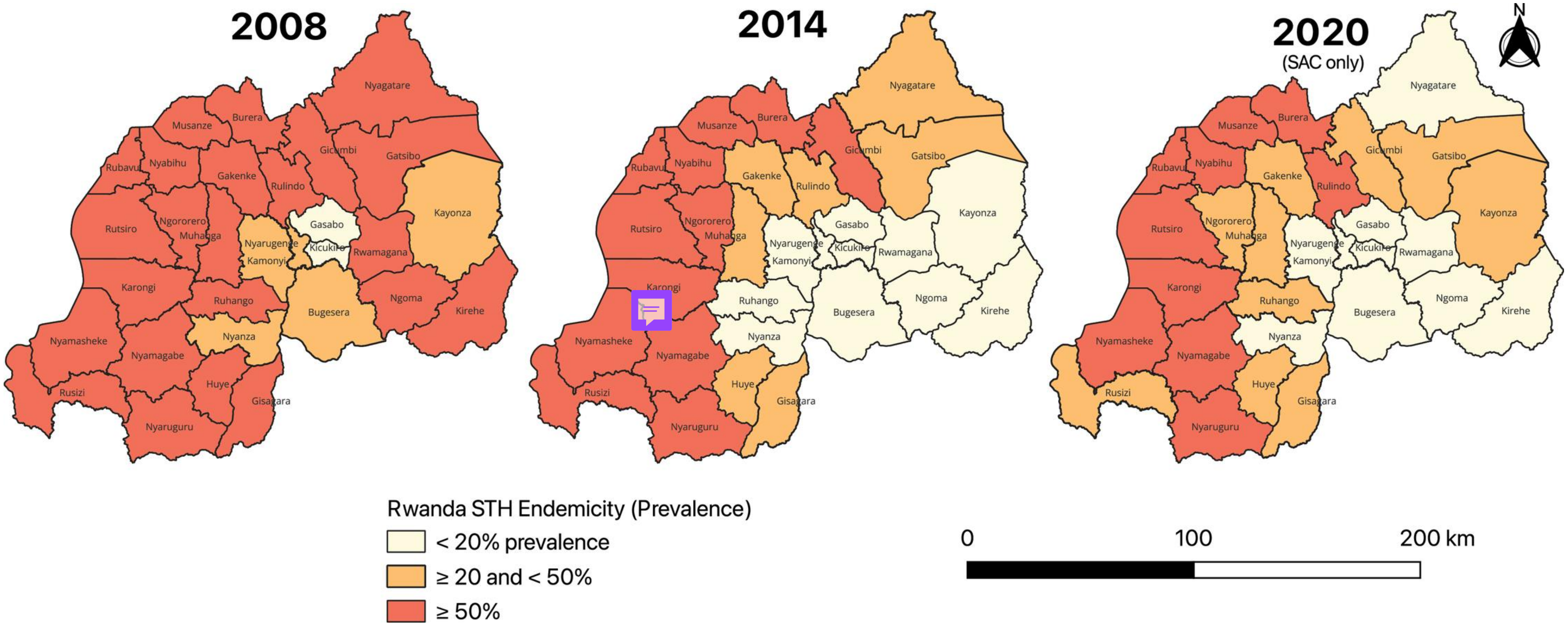

Fig 6: Trends in STH endemicity among SAC across Rwandan districts (2008–2020)

The map was created by DH using QGIS version 3.40.1-Bratislava. The base layer consists of shapefiles provided by the National Institute of Statistics of Rwanda and publicly available through the Africa GeoPortal, powered by Esri: https://rwanda.africageoportal.com/search?q=rwanda

Knowledge, attitudes and practices about STH and SCH

Table 6 summarizes sources of information, knowledge, and attitudes about STH and SCH among SAC and adults. Knowledge was high, with 90.6% of SAC and 96.6% of adults aware of correct transmission modes, and over 90% understanding proper treatment. However, 25% believed prevention was impossible, and 12.7% found using untreated human waste as fertilizer acceptable. For STH, SAC mainly received information from schools (69.1%), while adults relied on health facilities (54.1%) and media (32.9%).

The knowledge about SCH transmission was lower, with 52.6% of SAC and 50.1% of adults aware of correct transmission modes. SAC primarily learned from schools (36.8%) and media (30.3%), while adults accessed information from media (45.7%) and health facilities (38.4%). Still, there was strong support for regular screening (93.2%) and medication (93%), though 36.7% viewed SCH as a mild disease.

Table 6: Source of information, knowledge, and attitudes about SCH and STH

Variables	Categories	About SCH			About STH		
		SAC (N=152)	Adults (N=1382)	Total (N=1534)	SAC (N=1471)	Adults (N=4791)	Total (N=6262)
			n (%)			n (%)	
Source of information	Churches	1 (0.7)	6 (0.4)	7 (0.5)	9 (0.6)	41 (0.9)	50 (0.8)
	Community Health Workers	36 (23.7)	471 (34.1)	507 (33.1)			
	Health facility	44 (28.9)	531 (38.4)	575 (37.5)	369 (25.1)	2594 (54.1)	2963 (47.3)
	Media	46 (30.3)	631 (45.7)	677 (44.1)	260 (17.7)	1575 (32.9)	1835 (29.3)
	School	56 (36.8)	88 (6.4)	144 (9.4)	1017 (69.1)	364 (7.6)	1381 (22.1)
	Old parents	20 (13.2)	67 (4.8)	87 (5.7)	319 (21.7)	324 (6.8)	643 (10.3)
	Other	4 (2.6)	29 (2.1)	33 (2.2)	12 (0.8)	93 (1.9)	105 (1.7)
When last received STH information	1 year or less	61 (40.1)	439 (31.8)	500 (32.6)			
	> 1 year	91 (59.9)	943 (68.2)	1034 (67.4)			
Knows correct transmission mode		80 (52.6)	692 (50.1)	772 (50.3)	1333 (90.6)	4630 (96.6)	5963 (95.2)
Know correct treatment		*			1344 (91.4)	4549 (94.9)	5893 (94.1)
Participant agreed that:	SCH is a mild disease.	70 (46.1)	493 (35.7)	563 (36.7)	*		
	Regular screening for SCH is important.	134 (88.2)	1296 (93.8)	1430 (93.2)	*		
	Taking SCH medication is important.	136 (89.5)	1290 (93.3)	1426 (93)			
	Seeking care for symptoms like blood in stool or abdominal pain is necessary.	143 (94.1)	1358 (98.3)	1501 (97.8)	*		
	Intestinal worms cannot be prevented.	*			363 (24.7)	1205 (25.2)	1568 (25)
	Using untreated human waste as fertilizer is acceptable.	*			181 (12.3)	614 (12.8)	795 (12.7)
	Herbs are more effective than modern medicine for treating intestinal worms.	*			213 (14.5)	486 (10.1)	699 (11.2)

* Not assessed for this condition

Risk factors for any STH and SCH: results from Mixed Effect Logistic Regression Models

Table 7 shows the results from mixed effect logistic regression models identifying factors associated with STH and *S. mansoni* (SCH) infections, adjusted for other variables and accounting for variations among districts.

For STH, single individuals were more likely to be infected compared to married individuals (AOR: 1.74, 95% CI: 1.32–2.28, $p < 0.001$). Those with no education were more likely to have STH compared to those with primary education (AOR: 1.56, 95% CI: 1.37–1.78, $p < 0.001$), while individuals with secondary or higher education were less likely to be infected (AOR: 0.55, 95% CI: 0.37–0.81, $p = 0.003$). Additionally, unemployed individuals (AOR: 0.34, 95% CI: 0.23–0.51, $p < 0.001$) and self-employed/retired individuals (AOR: 0.53, 95% CI: 0.31–0.91, $p = 0.020$) were less likely to have STH compared to those in agriculture/farming. Households with toilet facilities deeper than 3 meters had lower odds of infection compared to those with shallower facilities (AOR: 0.78, 95% CI: 0.64–0.96, $p = 0.018$), and consistently treating drinking water was also linked to lower odds (AOR: 0.79, 95% CI: 0.68–0.92, $p = 0.003$). Individuals using treated human excreta as fertilizer were more likely to have STH compared to those not using it (AOR: 1.43, 95% CI: 1.22–1.66, $p < 0.001$), as were those using non-improved water sources compared to those using improved sources (AOR: 1.17, 95% CI: 1.02–1.34, $p = 0.024$). Living near marshlands used for other activities was also associated with a higher likelihood of STH infection (AOR: 1.17, 95% CI: 1.02–1.35, $p = 0.025$).

For SCH, single individuals were more likely to be infected compared to married individuals (AOR: 1.61, 95% CI: 1.11–2.33, $p = 0.011$), as were those without education compared to those with primary education (AOR: 1.41, 95% CI: 1.16–1.72, $p = 0.001$). Participants living more than 1 hour from a water source were more likely to have SCH compared to those living 30–60 minutes away (AOR: 1.42, 95% CI: 1.06–1.91, $p = 0.019$). Proximity to lakes was associated with a higher likelihood of SCH compared to those not living near lakes (AOR: 1.76, 95% CI: 1.37–2.27, $p < 0.001$). Additionally, individuals using untreated human excreta as manure were more likely to have SCH (AOR: 1.6, 95% CI: 1.06–2.41, $p = 0.027$), and those who treated the human excreta before use as manure also had higher odds of infection (AOR: 1.32, 95% CI: 1.06–1.65, $p = 0.014$). Living near marshlands used for rice farming was associated with a higher likelihood of SCH compared to those not near such marshlands (AOR: 1.31, 95% CI: 1.00–1.72, $p = 0.047$). The standard deviation for the random intercept (districts) 0.73 for SCH, indicating variation across districts.

Table 7: Results from the mixed effect logistic regression for the factors associated having any STH, and ~~factor associated with having SCH~~

Variables	Categories	Any STH			*S. mansoni* (KK or POC-CCA - Trace positive)		
		AOR	95% CI	P-value	AOR	95% CI	P-value
Age categories	Adults (16+)	Ref			Ref		
	SAC (5 - 15)	1.3	(0.53 - 3.16)	0.565	1.79	(0.61 - 5.24)	0.285
Marital status	Married	Ref			Ref		
	Single	1.74	(1.32 - 2.28)	<0.001*	1.61	(1.11 - 2.33)	0.011*
	Divorced/Separated/Widowed	1.42	(1.16 - 1.74)	0.001*	0.83	(0.6 - 1.14)	0.251
Education level	Primary	Ref			Ref		
	No education	1.56	(1.37 - 1.78)	<0.001*	1.41	(1.16 - 1.72)	0.001*
	Vocational or literacy training	0.68	(0.19 - 2.42)	0.554	1.27	(0.14 - 11.34)	0.830
	Secondary and higher	0.55	(0.37 - 0.81)	0.003*	0.8	(0.41 - 1.58)	0.526
Main occupation	Agriculture/Farmer	Ref			Ref		
	Employed (formal/informal)	1.03	(0.78 - 1.37)	0.829	1.1	(0.73 - 1.65)	0.663
	Self Employed/Retired	0.53	(0.31 - 0.91)	0.020*	1.06	(0.48 - 2.37)	0.880
	Student	0.54	(0.34 - 0.85)	0.008*	1.26	(0.68 - 2.35)	0.463
	Unemployed/Other	0.34	(0.23 - 0.51)	<0.001*	0.82	(0.44 - 1.54)	0.540
Has toilet facility	Yes, < 3 meters deep	Ref					
	Yes, 3+ meters deep	0.78	(0.64 - 0.96)	0.018*			
Always have soap and water	No	Ref					
	Yes	0.95	(0.84 - 1.08)	0.460			
Main water source	Improved source	Ref			Ref		
	Non-improved source	1.17	(1.02 - 1.34)	0.024*	0.71	(0.47 - 1.07)	0.099
Treat water prior to drinking	Never	Ref					
	Sometimes	0.84	(0.71 - 0.98)	0.027*			
	Yes, always	0.79	(0.68 - 0.92)	0.003*			
Time to closest water source (round trip)	30 - 60 min				Ref		
	More than 1 hour				1.42	(1.06 - 1.91)	0.019*
	On premises or <30 min				1.17	(0.91 - 1.49)	0.215
Use human excreta as fertilizer and treat it	No	Ref			Ref		
	Yes, and treat the fertilizer used	1.43	(1.22 - 1.66)	<0.001*	1.32	(1.06 - 1.65)	0.014*
	Yes, does not treat fertilizer used	1.19	(0.9 - 1.59)	0.228	1.6	(1.06 - 2.41)	0.027*
	Lake No				Ref		
	Yes				1.76	(1.37 - 2.27)	<0.001*

Variables	Categories		Any STH			*S. mansoni* (KK or POC-CCA - Trace positive)		
			AOR	95% CI	P-value	AOR	95% CI	P-value
Nearest water bodies	River	No				Ref		
		Yes				0.89	(0.7 - 1.12)	0.305
	Pond/Dam	No				Ref		
		Yes				0.96	(0.76 - 1.22)	0.748
	Marshlands for rice farming	No	Ref			Ref		
		Yes	1.02	(0.87 - 1.21)	0.777	1.31	(1 - 1.72)	0.047*
	Marshlands for other	No	Ref			Ref		
		Yes	1.17	(1.02 - 1.35)	0.025*	0.85	(0.68 - 1.07)	0.168
	Other water bodies	No				Ref		
		Yes				0.98	(0.56 - 1.73)	0.948
Walk time to closest water bodies	0-20 min					Ref		
	21-40 min					1.04	(0.83 - 1.31)	0.728
	41+ min					0.9	(0.67 - 1.23)	0.518
Standard deviation for the random intercept (Districts)			0.6			0.73		

Model limited to age >=10 years. Empty categories were not included in the model for the specific condition.
** Significant variables (p value <0.05)*

Discussion

Key results and interpretation

This nationwide survey provides an updated assessment of the prevalence and risk factors for STH and SCH across all age groups in Rwanda. STH prevalence remains high, with adults showing the highest rates and pre-SAC the lowest. Fourteen districts reported an MHI prevalence of ≥2% for at least one STH species among school-aged children, highlighting persistent challenges in achieving the threshold for elimination as a public health problem. Similarly, SCH prevalence remains significant, particularly when assessed using POC-CCA diagnostics, which reveal prevalence exceeding 10% at several sites, necessitating preventive chemotherapy. Despite this, heavy intensity SCH infections remain below 1% across all endemic units, suggesting progress toward elimination targets.

A higher likelihood of STH infection was associated with being single, having no education, using treated human excreta as manure, relying on unimproved water sources, and living near marshlands. Lower likelihoods were observed among those with higher education, unemployed or self-employed/retired individuals, households with deep toilet facilities, and those practicing consistent water treatment. Similarly, SCH infection risk was higher among individuals who were single, lacked education, lived near lakes or rice-farming marshlands, used untreated or treated human excreta as fertilizer, or resided over an hour from a water source. These results underscore persistent vulnerabilities and highlight areas for targeted interventions to reduce the burden of STH and SCH in Rwanda.

Persistence of high prevalence despite consistent MDA

Despite multiple rounds of high-coverage MDA, STH prevalence remains substantial in Rwanda. This persistence can be attributed to several key factors including limited access to clean water and sanitation, the exclusion of adults from MDA programs, and the limited efficacy of MDA drugs against certain helminths, particularly *T. trichiura*.

A major barrier to effective control of STH and SCH is the limited implementation of comprehensive Water, Sanitation, and Hygiene (WASH) measures to support MDA. Despite Rwanda's investments in hygiene and sanitation infrastructure, progress has been slow. From 2014 to 2020, the population using improved water sources increased only modestly, from 72.4% to 79.6%, while households with sanitation facilities declined slightly, from 71.2% to 70.2% [7,8]. Additionally, data on the quality, maintenance, and consistent use of WASH facilities remain limited. This stagnation suggests that high MDA coverage in SAC alone may be insufficient to interrupt transmission, especially in communities with inconsistent access to clean water and sanitation [33] Findings from this study underscore the need for WASH interventions that extend beyond MDA, emphasizing not only the availability of facilities but also their quality, regular maintenance, and reliable usage. Prioritizing these factors is essential for sustained control of STH and SCH transmission. Though requiring substantial investment and stakeholder collaboration, the long-term benefits are likely sustainable and impactful for infection control [34,35].

In addition to WASH and infrastructure gaps, the inclusion of adults in our survey revealed that adults have a higher STH prevalence (46.1%) compared to pre-SAC (30.2%). This finding aligns with studies from Benin, Malawi, Kenya, and India, highlighting that untreated adults may act as reservoirs for reinfection, undermining MDA efforts focused on children [9,10,36,37]. The higher prevalence in adults appears to be driven by elevated hookworm infections in this group and a notable prevalence of *A. lumbricoides* across all age groups. Historically, MDA in Rwanda has targeted pre-SAC and SAC as they are the most accessible and vulnerable populations. However, excluding adults leaves a substantial portion of the population untreated, enabling infections to persist and reinfect younger populations [38–40]. Expanding MDA to include adults could more effectively disrupt transmission and accelerate progress toward eliminating STH and SCH as public health problems [41].

However, including adults in MDA efforts presents logistical and financial challenges. The health benefits of switching to community-wide treatment depend on the STH species and baseline endemicity [42,43]. More importantly, WHO does not currently support drug donations for adult STH treatment in MDA programs, so expansion would require additional funding—a significant cost for the government given the scale needed [42,44,45]. While advocating for donor support to include adults, a phased approach may be effective, focusing on high-prevalence districts (e.g., ≥50% prevalence) where adult treatment could have the greatest impact. Targeting these areas first would help the government optimize resources while addressing the most urgent needs.

Alongside gaps in MDA coverage, the limited effectiveness of albendazole, the current MDA drug against *T. trichiura*, poses an additional challenge to STH control. Evidence shows that albendazole has low cure rates for *T. trichiura*, while combining albendazole with ivermectin achieves more promising results through higher egg reduction and cure rates [46]. Results in S2 Appendix and S3 Appendix indicate persistently high STH prevalence in areas with substantial *T. trichiura* burdens, likely due to the low efficacy of albendazole against this species. Our findings suggest that adopting combination therapies, such as albendazole with ivermectin, in *T. trichiura* high-prevalence areas could significantly improve MDA outcomes.

Factors associated with STH and SCH infection

Our findings highlight how socioeconomic, occupational, and environmental factors significantly influence the likelihood of STH and SCH infections, underscoring the need for targeted interventions to reduce transmission.

Socioeconomic factors such as education, water source quality, sanitation practices, and employment status emerged as key determinants of infection likelihood, similar to findings in Benin, India, Ghana, and Ethiopia, where lower-income groups face higher infection likelihood [36,37,47–49]. Generally, studies show that education correlates with economic status and better access to sanitation resources, helping to limit contact with STH and SCH pathogens and reduce exposure to contaminated materials. Individuals without formal education are more susceptible to infection, likely due to limited awareness of preventive practices like hand-washing and safe food handling, along with restricted access to clean water and

sanitation facilities. In contrast, those with higher education are more likely to adopt preventive behaviors, such as regular hand-washing and water treatment, which reduce infection risk [50].

Furthermore, reliance on unimproved water sources and the use of human excreta as manure were associated with a higher likelihood of infection. In lower-income communities, these practices often result from a lack of affordable, safer alternatives, increasing exposure to contaminants [47,51,52]. Households using treated human waste as manure may mistakenly assume it is safe, often relying on limited or inadequate protective measures and equipment. In some cases, they may adopt certain protective practices but wrongly believe these are sufficient, even when they are not effective. Expanding access to improved sanitation facilities, clean water sources, and modern fertilizers, along with raising awareness about the health risks associated with using human excreta, can promote safer waste disposal and hygiene practices, reducing contact with infectious agents and lowering infection rates.

Beyond socioeconomic factors, occupational and environmental exposures significantly influenced infection risk. Specifically, residing near marshlands, lakes, or rice-farming areas was strongly associated with higher infection rates, as these environments facilitate SCH transmission through snail vectors and support the survival of STH eggs and larvae [34]. Proximity to such water bodies increases exposure to contaminated water and soil, heightening the risk of infection. Evidence shows that adequate sanitation is associated with a lower likelihood of *S. mansoni* infection [53]. In this study, participants living farther from clean water sources had higher SCH prevalence, likely relying on untreated local water bodies for daily needs, which prolongs exposure to contamination [33]. These findings underscore the importance of context-specific interventions, including improving access to clean water and sanitation in high-risk areas.

In addition to environmental risks, occupational exposures play a critical role, particularly for individuals in jobs involving frequent contact with soil or water, such as farming and manual labor. These occupations lead to ongoing exposure to environments where STH eggs and SCH larvae thrive, making infection difficult to avoid without protective equipment or adequate sanitation facilities [34]. For example, rice farming, which requires prolonged water exposure, substantially increases infection risk [54–56]. Some studies have also demonstrated a correlation between helminth egg counts in wastewater and soil samples and infections in farmers[57]. Our study suggests that the persistence of hookworm among adults (21% prevalence compared to 4.3% and 6.1% among pre-SAC and SAC) may be linked to occupational and rural lifestyle factors, compounded by the exclusion of adults from MDA programs. These findings support the need for control strategies that extend beyond MDA, including promoting consistent shoe-wearing, improving WASH infrastructure, and providing targeted hygiene education to reduce infection risks and support efforts to lower morbidity in high-risk communities [1,2,53].

Importance of using precision mapping

This study highlights the critical role of precision mapping in improving the control of SCH and ~~soil-~~ STH. By integrating ecological, epidemiological, and exposure factors, precision mapping facilitates the efficient testing of individuals for both SCH and STH, offering valuable insights for program implementation [17–

20]. The 2020 remapping exercise demonstrated how targeted sampling across diverse ecological settings can accurately identify transmission patterns at the community level. This granularity supports the program's efforts to "shrink the map," focusing MDA efforts at the "cell" level (below the sector) rather than the broader sector level, thereby enhancing both efficiency and accuracy. This targeted approach ensures resources are allocated to high-risk communities, reducing unnecessary treatment in low-risk areas, cutting costs, and improving program effectiveness. By directing treatment to areas with confirmed need, this strategy aligns closely with SCH elimination goals [19,20,58].

While precision mapping reduces the number of individuals needing treatment by targeting high-risk areas, the use of more sensitive diagnostic tools, such as POC-CCA with trace-positive counting, can expand detection and increase the treatment population. This approach raises the number of areas meeting the prevalence threshold for treatment eligibility (≥10%), designating regions for intervention that might otherwise be classified as low-endemic if trace results were excluded. Additionally, as Rwanda transitions from control to transmission interruption goals, MDA efforts will need to include additional age groups, further increasing the treatment population and adding implementation complexity.

Despite these challenges, this study provides strong evidence supporting the scalability and utility of precision mapping for SCH elimination. By emphasizing efficiency, accuracy, and adaptability, precision mapping offers a robust, data-driven model for targeted interventions, enabling countries to optimize resources and advance toward SCH elimination goals effectively [19,20,58].

Strengths and limitations

This study is the first national, community-based survey of STH and SCH in Rwanda, providing a broader perspective compared to traditional school-based assessments. By including adults alongside the traditionally assessed pre-SAC and SAC participants, it offers a comprehensive view of infection prevalence across all age groups. A key strength of the study was the use of precision mapping, which provided a granular understanding of SCH, a focally distributed disease, resulting in more accurate prevalence estimates. Furthermore, the combined use of KK and POC-CCA methods enhanced SCH detection, identifying cases that might have been missed by KK stool testing alone. Rigorous quality assurance measures, including training, pre-testing, and daily checks, ensured the collection of reliable and high-quality data.

Despite its strengths, the study has some limitations. The reliance on self-reported data for some variables, such as sanitation practices, could introduce recall bias. Additionally, while the POC-CCA test offers greater sensitivity, particularly in low-endemic settings, it has batch-to-batch variations and may overestimate SCH prevalence due to false positives from trace readings, warranting further research to improve the accuracy of this diagnostic [59,60]. Future research should consider the WHO Target Product Profiles for schistosomiasis point-of-care products to address these challenges, focusing on developing reliable tools for low-prevalence areas. These TPPs aim to guide the creation of diagnostics that can accurately measure whether prevalence is above or below the 10% threshold, which is critical for determining MDA frequency [61].

Conclusion

This nationwide survey highlights that, despite ongoing MDA programs, STH and SCH remain significant public health challenges in Rwanda, particularly among adults and in high-risk regions. Structural and behavioral factors, such as limited education, the use of untreated human waste as manure, and proximity to lakes and marshlands, sustain transmission and reveal the limitations of traditional, school-based approaches. To effectively address these infections, MDA programs must be extended to include adults and integrated with efforts to improve sanitation, access to clean water, and community engagement in safe hygiene practices. Tackling these drivers and social determinants will enable more equitable and sustainable control, advancing toward the elimination of these infections.

Acknowledgments

We would like to express our sincere gratitude to all the participants who took part in this study, as well as the dedicated data collectors and field teams whose hard work made this research possible. We also appreciate the support of community health workers, local leaders, and health center staff who facilitated the smooth coordination of data collection across the districts.

References

1. World Health Organization (WHO). Soil-transmitted helminth infections [Internet]. Vol. 391, World Health Organization (WHO). 2023 [cited 2024 Jul 13]. p. 252–65. Available from: https://www.who.int/news-room/fact-sheets/detail/soil-transmitted-helminth-infections
2. World Health Organization (WHO). Schistosomiasis. Organisation, World Health. 2023.
3. Ministry of Health Rwanda - Rwanda Biomedical Center. Rwanda Malaria and Neglected Tropical Diseases Annual Report 2018-2019. 2019.
4. Ministry of Health Rwanda - Rwanda Biomedical Center (RBC). Neglected Tropical Diseases Strategic Plan 2019-2024 [Internet]. Kigali, Rwanda; 2019 [cited 2020 Jul 5]. Available from: https://rbc.gov.rw/fileadmin/user_upload/guide2019/guide2019/RWANDA NTD STRATEGIC PLAN 2019-2024.pdf
5. Kabatende J, Mugisha M, Ntirenganya L, Barry A, Ruberanziza E, Mbonigaba JB, et al. Prevalence, Intensity, and Correlates of Soil-Transmitted Helminth Infections among School Children after a Decade of Preventive Chemotherapy in Western Rwanda. Pathogens. 2020 Dec;9(12).
6. Ruberanziza E, Owada K, Clark NJ, Umulisa I, Ortu G, Lancaster W, et al. Mapping Soil-Transmitted Helminth Parasite Infection in Rwanda: Estimating Endemicity and Identifying At-Risk Populations. Trop Med Infect Dis. 2019 Jun;4(2).
7. National Institute of Statistics of Rwanda (NISR), Ministry of Health (MOH) [Rwanda] and II. Rwanda Demographic and Health Survey 2014-15 Final Report. Rockville, Maryland, USA: NISR, MOH, and ICF International; 2015.
8. National Institute of Statistics of Rwanda (NISR), Ministry of Health (MOH) [Rwanda] and II. Rwanda Demographic and Health Survey 2019-20 Final Report. Vol. 53, National Institute of Statistics of Rwanda (NISR),. 2020.
9. DeWorm3 Trials Team. Baseline patterns of infection in regions of Benin, Malawi and India seeking to interrupt transmission of soil transmitted helminths (STH) in the DeWorm3 trial. PLoS Negl Trop Dis. 2020 Nov;14(11):e0008771.

10. Njenga SM, Mwandawiro CS, Muniu E, Mwanje MT, Haji FM, Bockarie MJ. Adult population as potential reservoir of NTD infections in rural villages of Kwale district, Coastal Kenya: implications for preventive chemotherapy interventions policy. Parasit Vectors [Internet]. 2011;4(1):175. Available from: https://doi.org/10.1186/1756-3305-4-175
11. Toor J, Turner HC, Truscott JE, Werkman M, Phillips AE, Alsallaq R, et al. The design of schistosomiasis monitoring and evaluation programmes: The importance of collecting adult data to inform treatment strategies for Schistosoma mansoni. PLoS Negl Trop Dis. 2018 Oct;12(10):e0006717.
12. Clark NJ, Umulisa I, Ruberanziza E, Owada K, Colley DG, Ortu G, et al. Mapping Schistosoma mansoni endemicity in Rwanda: a critical assessment of geographical disparities arising from circulating cathodic antigen versus Kato-Katz diagnostics. PLoS Negl Trop Dis [Internet]. 2019 Sep 30;13(9):e0007723. Available from: https://doi.org/10.1371/journal.pntd.0007723
13. Ruberanziza E, Wittmann U, Mbituyumuremyi A, Mutabazi A, Campbell CH, Colley DG, et al. Nationwide Remapping of Schistosoma mansoni Infection in Rwanda Using Circulating Cathodic Antigen Rapid Test: Taking Steps toward Elimination. Am J Trop Med Hyg. 2020 Jul;103(1):315–24.
14. Rujeni N, Morona D, Ruberanziza E, Mazigo HD. Schistosomiasis and soil-transmitted helminthiasis in Rwanda: an update on their epidemiology and control. Infect Dis Poverty. 2017 Mar;6(1):8.
15. Nyandwi E, Osei FB, Veldkamp T, Amer S. Modeling schistosomiasis spatial risk dynamics over time in Rwanda using zero-inflated Poisson regression. Sci Rep [Internet]. 2020;10(1):19276. Available from: https://doi.org/10.1038/s41598-020-76288-8
16. Mather W, Hutchings P, Budge S, Jeffrey P. Association between water and sanitation service levels and soil-transmitted helminth infection risk factors: a cross-sectional study in rural Rwanda. Trans R Soc Trop Med Hyg. 2020 May;114(5):332–8.
17. World Health Organization (WHO). Assessing schistosomiasis and soil-transmitted helminthiases control programmes Monitoring and evaluation framework. Geneva: World Health Organization; 2024.
18. Baker MC, Mathieu E, Fleming FM, Deming M, King JD, Garba A, et al. Mapping, monitoring, and surveillance of neglected tropical diseases: towards a policy framework. The Lancet [Internet]. 2010 Jan 16;375(9710):231–8. Available from: https://doi.org/10.1016/S0140-6736(09)61458-6
19. Tchuem Tchuenté LA, Stothard JR, Rollinson D, Reinhard-Rupp J. Precision mapping: An innovative tool and way forward to shrink the map, better target interventions, and accelerate toward the elimination of schistosomiasis. PLoS Negl Trop Dis. 2018 Aug;12(8):e0006563.
20. Kepha S, Ochol D, Wakesho F, Omondi W, Njenga SM, Njaanake K, et al. Precision mapping of schistosomiasis and soil-transmitted helminthiasis among school age children at the coastal region, Kenya. PLoS Negl Trop Dis. 2023 Jan;17(1):e0011043.
21. National Institute of Statistics of Rwanda (NISR). The Fifth Rwanda Population and Housing Census, Main Indicators Report [Internet]. Kigali- Rwanda; 2023. Available from: https://www.statistics.gov.rw/publication/main_indicators_2022
22. Ministry of Finance and Economic Planning and National Institute of Statistics of Rwanda. Fifth Rwanda Population and Housing Census, 2022 - Thematic Report - POPULATION PROJECTIONS [Internet]. Kigali; 2023. Available from: https://www.statistics.gov.rw/publication/rphc5-thematic-report-population-projection
23. National Institute of Statistics of Rwanda. GDP National Accounts, 2020 [Internet]. Kigali-Rwanda; 2021 [cited 2021 Aug 23]. Available from: https://www.statistics.gov.rw/file/9561/download?token=MRWeHiMu

24. National Institute of Statistics of Rwanda. GDP National Accounts, 2023 [Internet]. Kigali-Rwanda; 2024. Available from: https://www.statistics.gov.rw/publication/2114
25. Government of Rwanda. Government of Rwanda: Administrative structure [Internet]. Government of Rwanda. 2021 [cited 2021 Jun 9]. Available from: https://www.gov.rw/government/administrative-structure
26. Garba A, Barkiré N, Djibo A, Lamine MS, Sofo B, Gouvras AN, et al. Schistosomiasis in infants and preschool-aged children: Infection in a single Schistosoma haematobium and a mixed S. haematobium-S. mansoni foci of Niger. Acta Trop. 2010 Sep;115(3):212–9.
27. World Health Organization (WHO). Guide for Mapping Neglected Tropical Diseases Amenable to Preventive Chemotherapy in the African Region [Internet]. Brazaville: WHO | Regional Office for Africa; 2018. p. 1–56. Available from: https://www.eliminateschisto.org/sites/gsa/files/content/attachments/2020-02-13/AFRO NTD Mapping Guide vFeb2014_Revised March 2018.pdf
28. The Ministry of Agriculture and Animal Resources. AboutThe Ministry of Agriculture and Animal Resources (MINAGRI) [Internet]. Government of Rwanda. Available from: https://www.minagri.gov.rw/about
29. National Agricultural Export Development Board. About the National Agricultural Export Development Board [Internet]. Government of Rwanda. Available from: https://www.naeb.gov.rw/about-naeb/overview
30. World Health Organization (WHO. Workshop on schistosomiasis sub-district level data review for shrinking the map; better utilization of available prevalenece data and sub-district level planning for selected countries - Data Analysis Summary Report [Internet]. 2019. Available from: https://espen.afro.who.int/system/files/content/resources/Data Analysis Summary Report.pdf
31. World Health Organization (WHO). Helminth control in school-age children: a guide for managers of control programmes. 2nd edition [Internet]. 2011 [cited 2022 Sep 9]. Available from: https://www.who.int/publications/i/item/9789241548267
32. Bosch F, Palmeirim MS, Ali SM, Ame SM, Hattendorf J, Keiser J. Diagnosis of soil-transmitted helminths using the Kato-Katz technique: What is the influence of stirring, storage time and storage temperature on stool sample egg counts? PLoS Negl Trop Dis. 2021 Jan;15(1):e0009032.
33. Vaz Nery S, Pickering AJ, Abate E, Asmare A, Barrett L, Benjamin-Chung J, et al. The role of water, sanitation and hygiene interventions in reducing soil-transmitted helminths: interpreting the evidence and identifying next steps. Parasit Vectors [Internet]. 2019;12(1):273. Available from: https://doi.org/10.1186/s13071-019-3532-6
34. Hotez P, Bundy D, Beegle K, Brooker S, Drake L. Helminth infections: soil-transmitted helminth infections and schistosomiasis. World Bank; 2006.
35. Oswald WE, Halliday KE, Mcharo C, Witek-McManus S, Kepha S, Gichuki PM, et al. Domains of transmission and association of community, school, and household sanitation with soil-transmitted helminth infections among children in coastal Kenya. PLoS Negl Trop Dis. 2019 Nov;13(11):e0007488.
36. Avokpaho EFGA, Houngbégnon P, Accrombessi M, Atindégla E, Yard E, Rubin Means A, et al. Factors associated with soil-transmitted helminths infection in Benin: Findings from the DeWorm3 study. PLoS Negl Trop Dis. 2021 Aug;15(8):e0009646.
37. Ajjampur SSR, Kaliappan SP, Halliday KE, Palanisamy G, Farzana J, Manuel M, et al. Epidemiology of soil transmitted helminths and risk analysis of hookworm infections in the community: Results from the DeWorm3 Trial in southern India. PLoS Negl Trop Dis [Internet]. 2021 Apr 30;15(4):e0009338. Available from: https://doi.org/10.1371/journal.pntd.0009338

38. Assefa G, Alemu M, Ayehu A. High Prevalence of Hookworm Species and Associated Factors among Soil-Transmitted Helminth-Infected Household Contacts in Burie Zuria District, Northwest Ethiopia: A Community-Based Cross-Sectional Study. Biomed Res Int. 2023;2023:6553913.
39. Okoyo C, Nikolay B, Kihara J, Simiyu E, Garn J V, Freeman MC, et al. Monitoring the impact of a national school based deworming programme on soil-transmitted helminths in Kenya: the first three years, 2012 - 2014. Parasit Vectors. 2016 Jul;9(1):408.
40. Ajjampur SSR, Kaliappan SP, Halliday KE, Palanisamy G, Farzana J, Manuel M, et al. Epidemiology of soil transmitted helminths and risk analysis of hookworm infections in the community: Results from the deworm3 trial in southern india. PLoS Negl Trop Dis. 2021;15(4):1–24.
41. Pullan RL, Halliday KE, Oswald WE, Mcharo C, Beaumont E, Kepha S, et al. Effects, equity, and cost of school-based and community-wide treatment strategies for soil-transmitted helminths in Kenya: a cluster-randomised controlled trial. The Lancet [Internet]. 2019 May 18;393(10185):2039–50. Available from: https://doi.org/10.1016/S0140-6736(18)32591-1
42. Turner HC, Bundy DAP. Programmatic implications of the TUMIKIA trial on community-wide treatment for soil-transmitted helminths: further health economic analyses needed before a change in policy. Vol. 13, Parasites & vectors. England; 2020. p. 102.
43. Truscott JE, Hardwick RJ, Werkman M, Saravanakumar PK, Manuel M, Ajjampur SSR, et al. Forecasting the effectiveness of the DeWorm3 trial in interrupting the transmission of soil-transmitted helminths in three study sites in Benin, India and Malawi. Parasit Vectors [Internet]. 2021;14(1):1–13. Available from: https://doi.org/10.1186/s13071-020-04572-7
44. World Health Organization (WHO. Frequently asked questions on the joint application package for preventive chemotherapy Planning, requesting medicines and reporting [Internet]. Geneva; 2023. Available from: https://www.who.int/publications/m/item/faqs-the-joint-application-package-for-preventive-chemotherapy-planning-requesting-medicines-and-reporting
45. World Health Organization (WHO). Guideline: preventive chemotherapy to control soil-transmitted helminth infections in at-risk population groups [Internet]. World Health Organization (WHO). Geneva: World Health Organization; 2017. Available from: https://www.who.int/publications/i/item/9789241550116
46. Palmeirim MS, Hürlimann E, Knopp S, Speich B, Belizario VJ, Joseph SA, et al. Efficacy and safety of co-administered ivermectin plus albendazole for treating soil-transmitted helminths: A systematic review, meta-analysis and individual patient data analysis. PLoS Negl Trop Dis. 2018 Apr;12(4):e0006458.
47. Gurmassa BK, Gari SR, Solomon ET, Goodson ML, Walsh CL, Dessie BK, et al. Prevalence and risk factors of soil transmitted helminths among vegetable farmers of Akaki river bank, Addis Ababa, Ethiopia. BMC Infect Dis. 2024 Sep;24(1):961.
48. Zerdo Z, Bastiaens H, Anthierens S, Massebo F, Masne M, Biresaw G, et al. Prevalence, intensity and endemicity of intestinal schistosomiasis and soil-transmitted helminthiasis and its associated factors among school-aged children in Southern Ethiopia. Sci Rep [Internet]. 2022;12(1):4586. Available from: https://doi.org/10.1038/s41598-022-08333-7
49. Cunningham LJ, Campbell SJ, Armoo S, Koukounari A, Watson V, Selormey P, et al. Assessing expanded community wide treatment for schistosomiasis: Baseline infection status and self-reported risk factors in three communities from the Greater Accra region, Ghana. PLoS Negl Trop Dis [Internet]. 2020 Apr 27;14(4):e0007973. Available from: https://doi.org/10.1371/journal.pntd.0007973
50. Witek-McManus S, Simwanza J, Chisambi AB, Kepha S, Kamwendo Z, Mbwinja A, et al. Epidemiology of soil-transmitted helminths following sustained implementation of routine preventive chemotherapy: Demographics and baseline results of a cluster randomised trial in southern Malawi. PLoS Negl Trop Dis. 2021 May;15(5):e0009292.

51. Humphries DL, Stephenson LS, Pearce EJ, The PH, Dan HT, Khanh LT. The use of human faeces for fertilizer is associated with increased intensity of hookworm infection in Vietnamese women. Trans R Soc Trop Med Hyg. 1997;91(5):518–20.
52. Moya B, Parker A, Sakrabani R. Challenges to the use of fertilisers derived from human excreta: The case of vegetable exports from Kenya to Europe and influence of certification systems. Food Policy [Internet]. 2019;85:72–8. Available from: https://www.sciencedirect.com/science/article/pii/S0306919218301520
53. Grimes JET, Croll D, Harrison WE, Utzinger J, Freeman MC, Templeton MR. The relationship between water, sanitation and schistosomiasis: a systematic review and meta-analysis. PLoS Negl Trop Dis. 2014 Dec;8(12):e3296.
54. Mutombo N, Landouré A, Man WY, Fenwick A, Dembélé R, Sacko M, et al. The association between child Schistosoma spp. infections and morbidity in an irrigated rice region in Mali: A localized study. Acta Trop. 2019 Nov;199:105115.
55. Matthys B, Tschannen AB, Tian-Bi NT, Comoé H, Diabaté S, Traoré M, et al. Risk factors for Schistosoma mansoni and hookworm in urban farming communities in western Côte d'Ivoire. Trop Med Int Health. 2007 Jun;12(6):709–23.
56. Kazura JW, Neill M, Peters PA, Dennis E. Swamp rice farming: possible effects on endemicity of schistosomiasis mansoni and haematobia in a population in Liberia. Am J Trop Med Hyg. 1985 Jan;34(1):107–11.
57. Gurmassa BK, Gari SR, Solomon ET, Goodson ML, Walsh CL, Dessie BK, et al. Distribution of helminth eggs in environmental and stool samples of farming households along Akaki River in Addis Ababa, Ethiopia. Trop Med Health. 2023 Nov;51(1):67.
58. Mazigo HD, Zinga MM, Kepha S, Yard E, McRee-Mckee K, Kabona G, et al. Precision and geographical prevalence mapping of schistosomiasis and soil-transmitted helminthiasis among school-aged children in selected districts of north-western Tanzania. Parasit Vectors [Internet]. 2022;15(1):492. Available from: https://doi.org/10.1186/s13071-022-05547-6
59. Colley DG, King CH, Kittur N, Ramzy RMR, Secor WE, Fredericks-James M, et al. Evaluation, Validation, and Recognition of the Point-of-Care Circulating Cathodic Antigen, Urine-Based Assay for Mapping Schistosoma mansoni Infections. Am J Trop Med Hyg. 2020 Jul;103(1_Suppl):42–9.
60. Graeff-Teixeira C, Favero V, Pascoal VF, de Souza RP, Rigo F de V, Agnese LHD, et al. Low specificity of point-of-care circulating cathodic antigen (POCCCA) diagnostic test in a non-endemic area for schistosomiasis mansoni in Brazil. Acta Trop [Internet]. 2021;217:105863. Available from: https://www.sciencedirect.com/science/article/pii/S0001706X21000425
61. World Health Organization. Diagnostic target product profiles for monitoring, evaluation and surveillance of schistosomiasis control programmes [Internet]. Geneva: World Health Organization; Available from: https://www.who.int/publications/i/item/9789240031104

Legends for supporting Information files

S1 Appendix. Age-Adjusted Prevalence of Soil-Transmitted Helminths and Schistosoma mansoni

S2 Appendix. District level Prevalence and intensity by species

S3 Appendix. Prevalence of Hookworm, *A. lumbricoides*, *T. trichiura*, and *S. mansoni* (KK, CCA Trace Positive/Negative) by sociodemographic characteristics.

S4 Appendix. Summary results of 2008, 2014, and 2020 surveys used for comparison

Authors contributions

The study was conceived by LN, AM, JBM, EN, AM, LU, MK, EH, NR, ER, RH, and PM. Field data collection, including laboratory procedures and quality assurance, was overseen by LN, JBM, EN, ER, SE, TM, JBM, MT, EU, JK, RH, and PM. Technical support was provided by AO, KP, ER, JU, TH, JT, and WL. Data cleaning, analysis, and drafting of the manuscript were conducted by DH, with detailed review and revision by LN, ER, and AO. All authors reviewed and approved the final version of the manuscript.

Availability of data and materials

All data supporting the findings of this study are included in the article. The dataset is held by the Rwanda Biomedical Center and cannot be publicly shared due to restrictions imposed by the Rwanda Ethics Committee. Participant consent did not permit sharing data beyond the research team, and public disclosure would violate these terms. However, access to the data may be granted upon request, pending approval from the Rwanda Biomedical Center and the Rwanda Ethics Committee. To request access, please contact the Rwanda Biomedical Center at info@rbc.gov.rw, providing a detailed rationale for your request.

Competing interests

AO, KP, JT, WL, and ER are affiliated with The END Fund, which funded the study and provided technical support. They did not conduct the data analysis but reviewed the results and contributed to the manuscript. All other authors declare no competing interests.

Funding

This study was funded by The END Fund (https://end.org/) through a grant to the Ministry of Health, Rwanda Biomedical Center – The Malaria, Neglected Tropical Diseases, and Other Parasitic Diseases Division (MOPDD). The study was independently implemented by the Rwanda Biomedical Center – MOPDD. The END Fund provided technical support in designing the study and reviewing the study results and manuscript but had no involvement in the data analysis.

Fig 1 Selected Villages

Click here to access/download;Figure;Fig 1 Selected villages across districts highlighted in red.tif

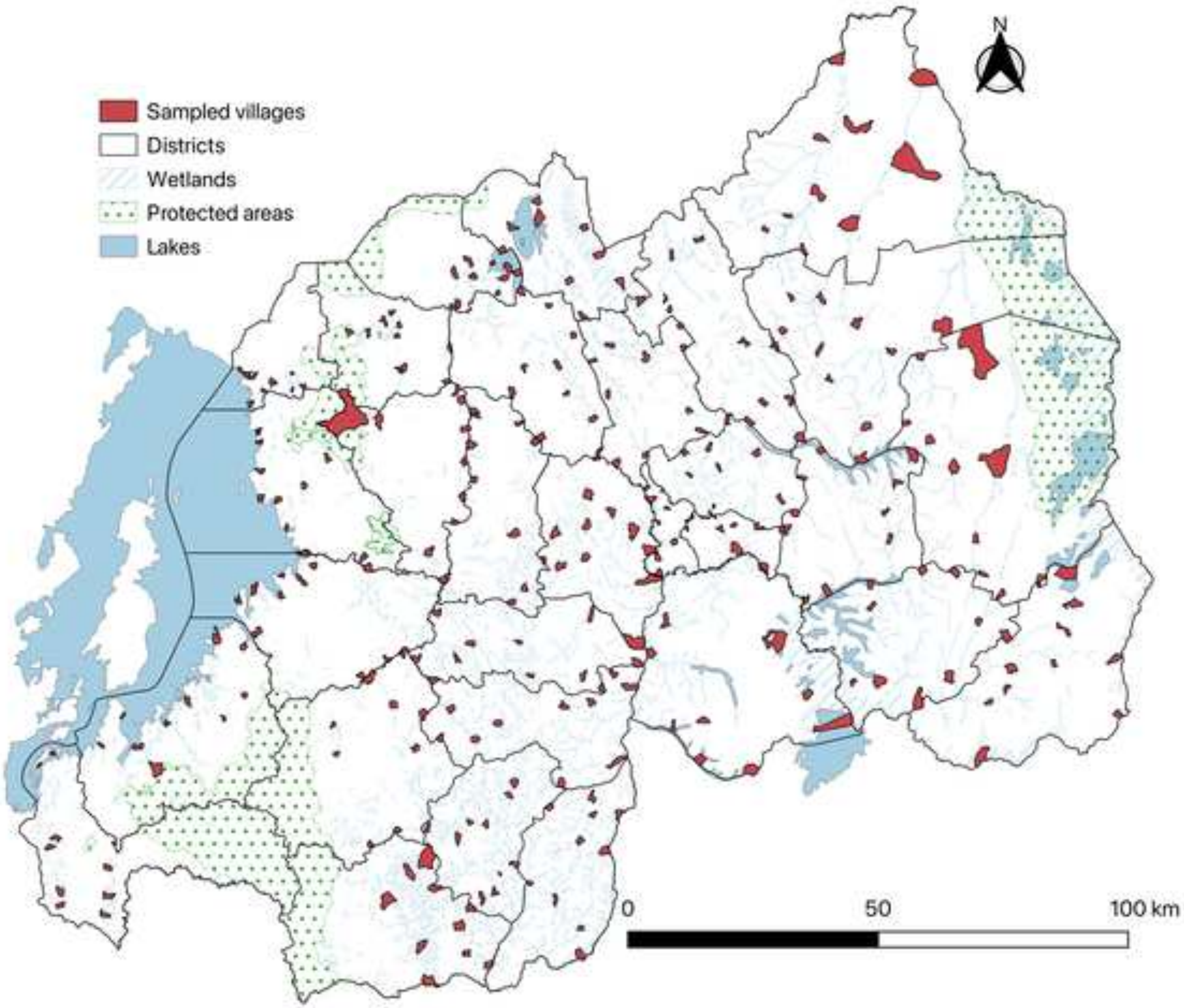

Fig 2 Site-Prevalences

Click here to access/download;Figure;Fig 2 Site-specific prevalence across Rwanda..tif

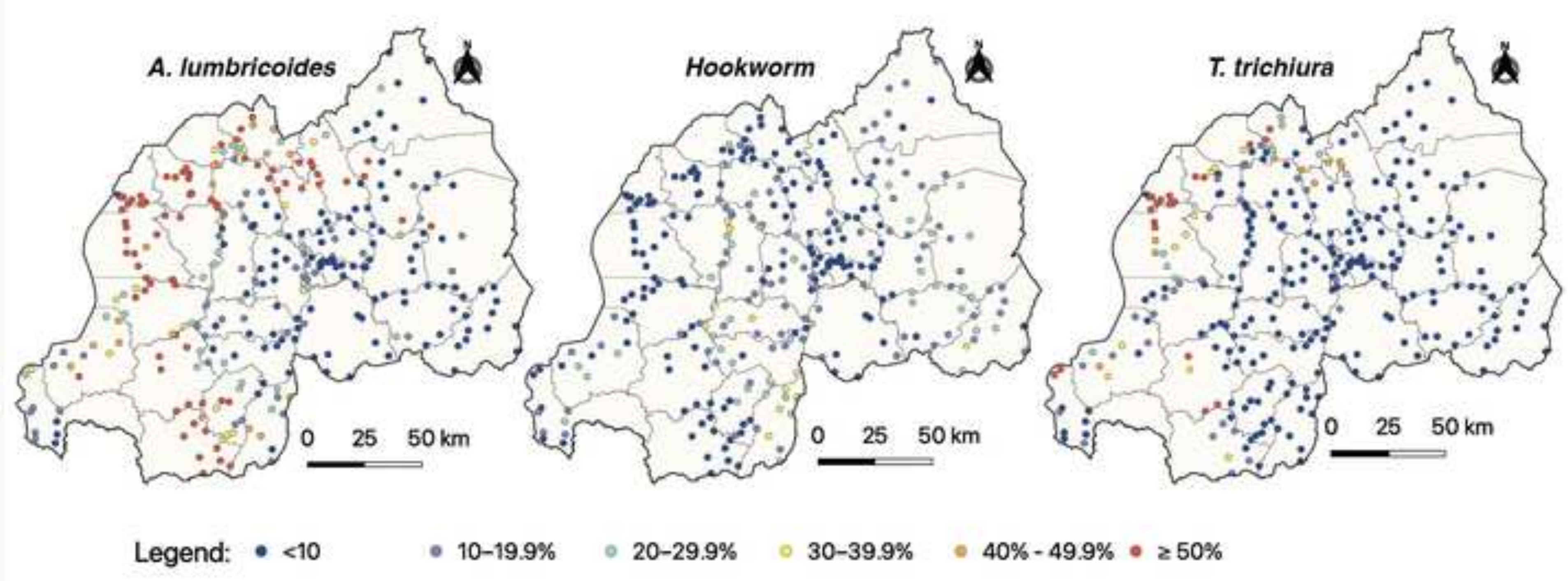

Fig 3 Sites specific S. mansoni prevalence

Click here to access/download;Figure;Fig 3 Sites specific S. mansoni prevalence.tif

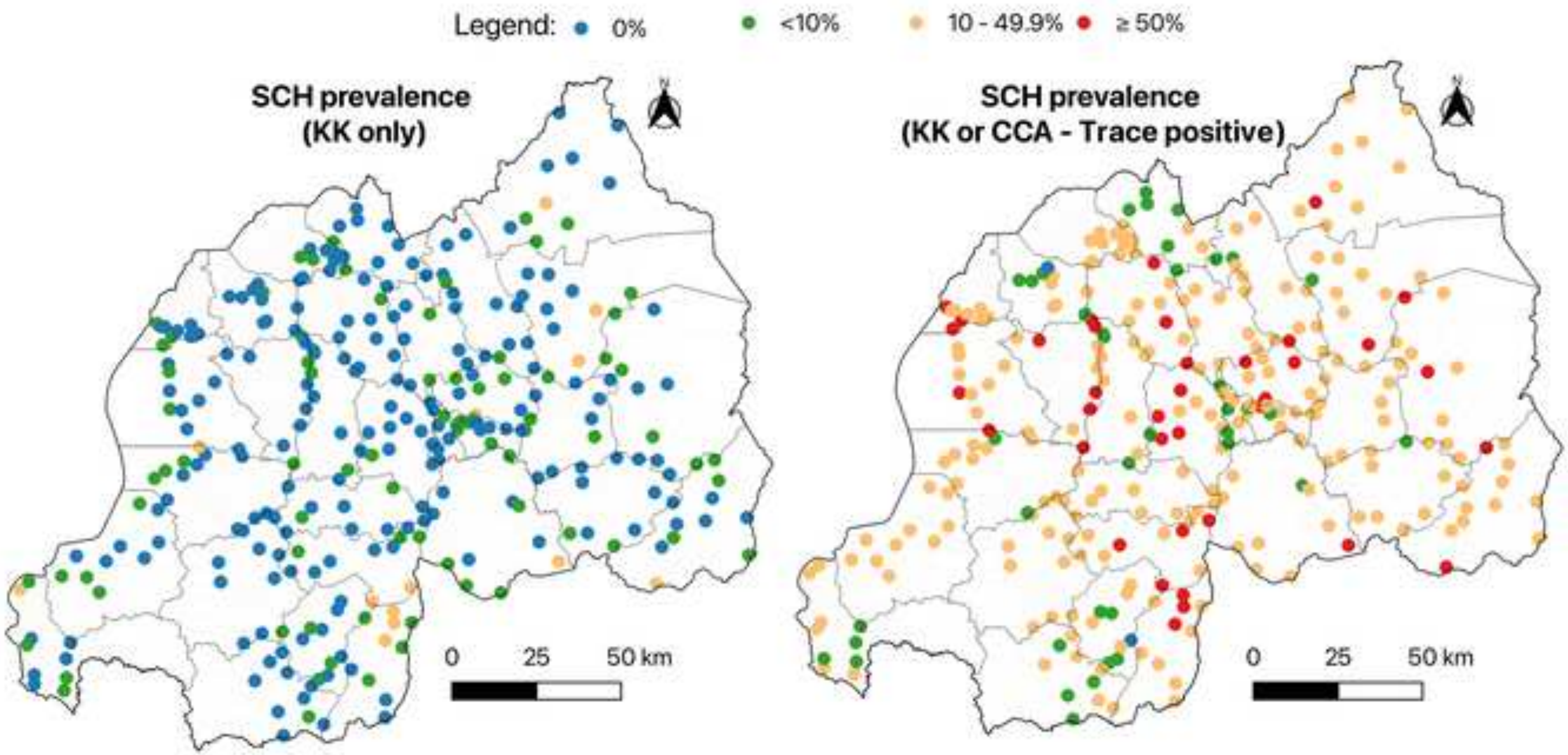

Fig 4 Prevalence of any STH by district

Click here to access/download;Figure;Fig 4 Prevalence of any STH by district.tif

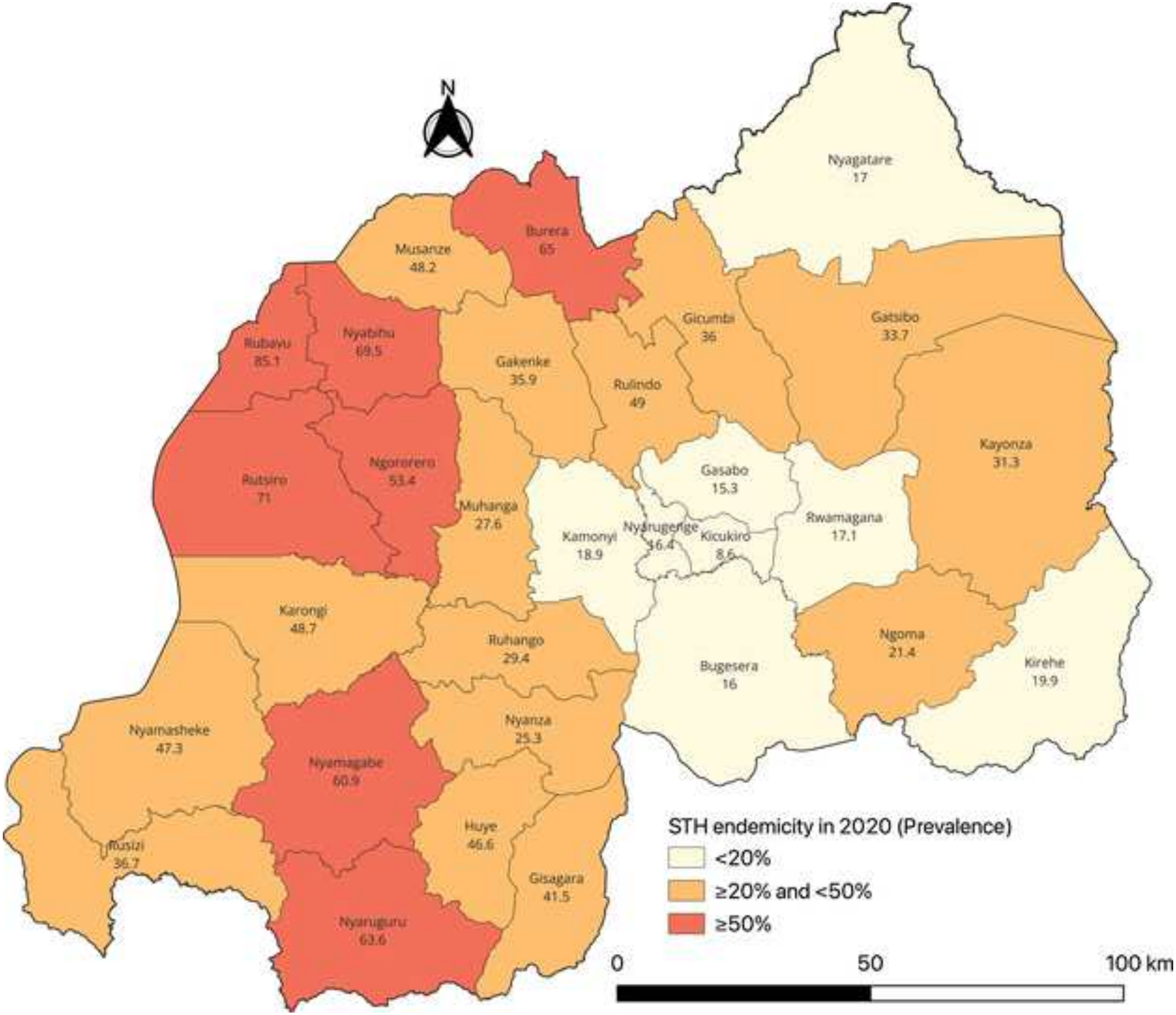

Fig 5 SCH and STH surveys -years
Click here to access/download;Figure;Fig 5 SCH and STH surveys -years.tif

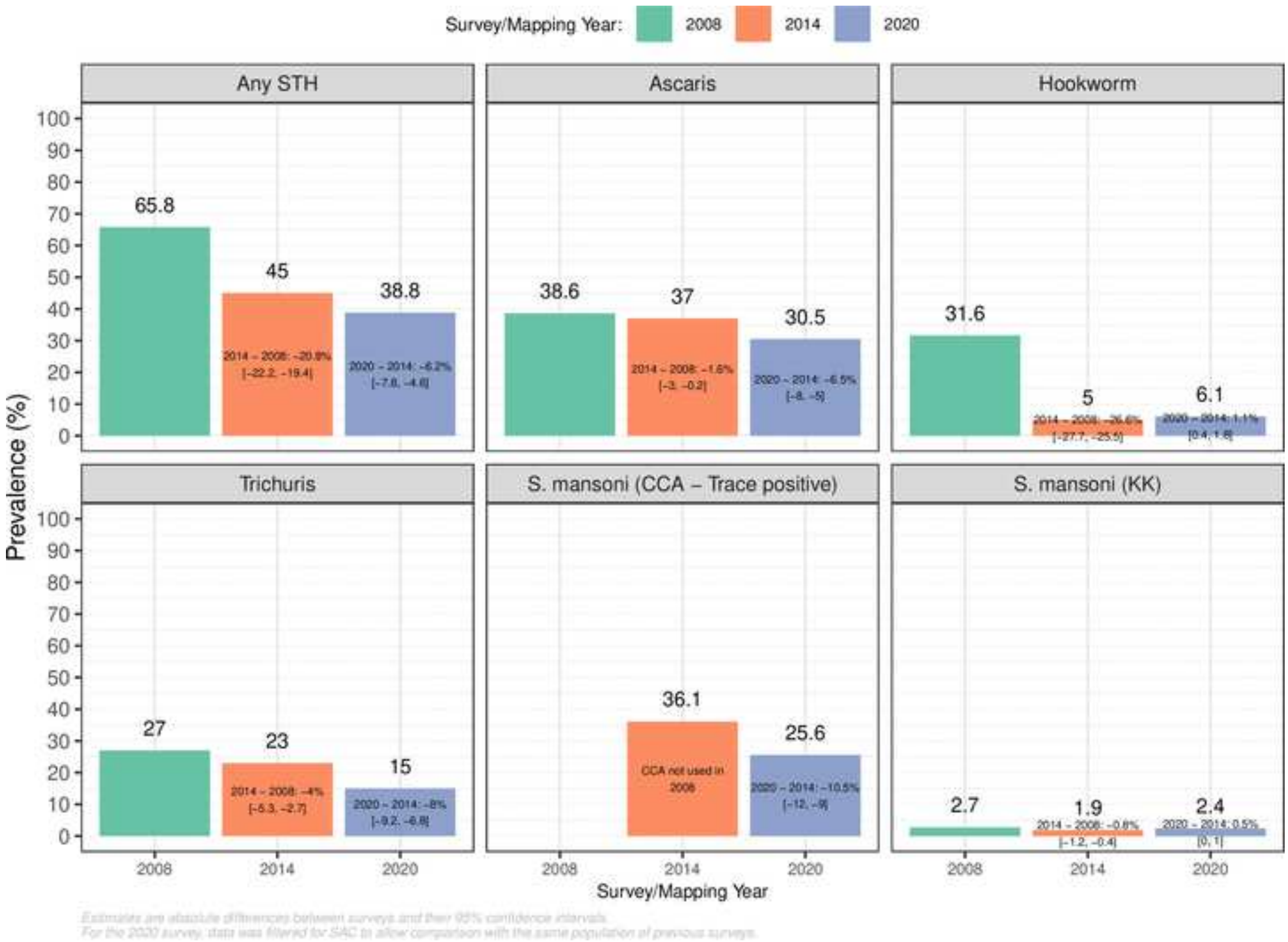

Fig 6 Trends in STH endemicity (2008–2020)

Click here to access/download;Figure;renamed_0dbef.tif

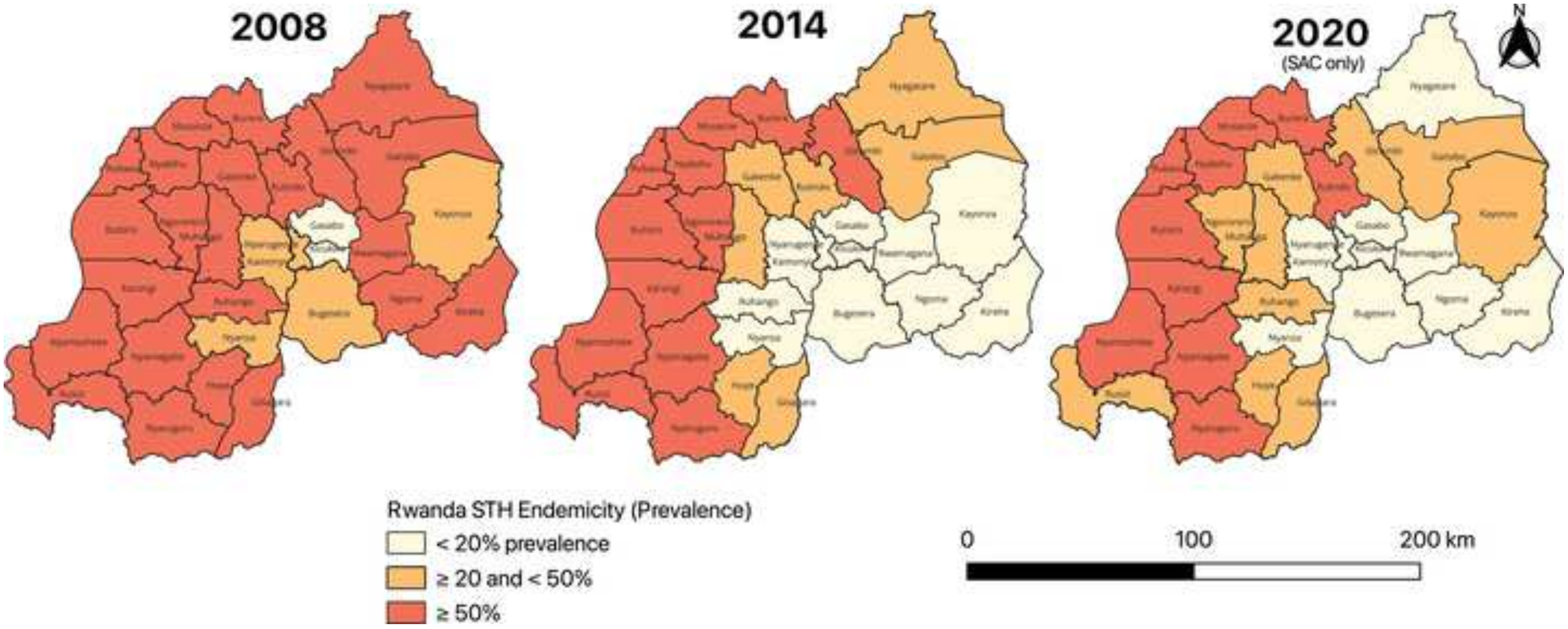

Click here to access/download
Supporting Information - Compressed/ZIP File Archive
Appendix- STH_SCH paper.zip
