# Supplementary material for: Remapping parasite landscapes: Nationwide prevalence, intensity and risk factors of schistosomiasis and soil-transmitted helminthiasis in Rwanda
Source: PLoS Negl Trop Dis. 2025 Aug 25;19(8):e0013328. doi: 10.1371/journal.pntd.0013328 (PMC12377619; doi:10.1371/journal.pntd.0013328)
Supplement: Attachment — Submitted filename: Review Comments.docx [file pntd.0013328.s008.docx]

**Remapping Parasite Landscapes: A Nationwide Precision Mapping Of Schistosomiasis and Soil-Transmitted Helminthiasis and Associated Risk Factors in Rwanda**

This STH/SCH remapping paper provides a good overview of the burden of these parasites in Rwanda. The paper explains why it is necessary to expand MDA to include the adult population. I have the following general, minor, and major comments for the authors. Detailed comments are embedded within the manuscript.pdf file.

**General Comments**

The findings section of the abstract is long and needs to be summarized into key findings only.

The manuscript needs proofreading to identify and correct sentence construction and grammatical errors

All comments in track changes are given in the pdf document.

**Minor Comments**

Lines 151-152: What does the existing national treatment guideline say on SCH treatment for individuals below 5 years?

Line 185: Indicate which species of SCH was tested using POC-CCA

Lines 189-190: This is a classification of *S. mansoni*; how was the intensity of *S. haematobium* classified?

Write the species' scientific names in italics throughout the text

Line 210: Why was the questionnaire administered to individuals aged 10 years and above only? Couldn’t this be administered to children below 10 years as well through their guardians?

Line 282: Where was the server located? How was compliance with local data protection laws ensured?

Lines 349-350: Is the assumption that STH is homogeneous across districts supported by past studies? Include the references supporting this assumption.

Table 5: Report test statistic results as: test-statistic used, test statistic result and p-value (e.g., X^2^ = 12.435, p<0.001).

Lines 506-507: Were the previous *S. mansoni* surveys (2008, 2014, and 2020) assessed using CCA as well? Provide citations to those previous surveys. It is important to compare infection results diagnosed using similar diagnostic technique

Line 550-563, and 565-575: Describe all risk factors first, then protective factors.

Line 702: I have not seen how this mapping has targeted ecological or environmental factors, but only geographical boundaries.

Line 982: In line with open science, properly de-identified and anonymized datasets need to be deposited in an open, freely accessible public repository.

**Major Comments**

Lines 155-157: Clearly describe what precision mapping entails. Any reference on the methodology of precision mapping? Include the dates when the survey was carried out.

Include the data collection tools, e.g., questionnaires and lab reporting forms, as supplementary materials. Were the tools used validated?

Line 181: Was *S. haematobium* assessed in this study? Which diagnostic technique was used to assess it?

Line 359: Indicate which statistical model was used to calculate the infection prevalence.

Clearly describe in the statistical methods how infection intensity was modelled.

Figs 2, 3 and 4: When plotting the point estimates and district prevalence on the map, use prevalence classification categories that align with WHO classification. This will aid programmatic decisions and comparability. See the M&E framework (Assessing schistosomiasis and soil-transmitted helminthiases control programmes: monitoring and evaluation framework. Geneva: World Health Organization; 2024. Licence: CC BY-NC-SA 3.0 IGO.).

Lines 500-502: Clearly describe in the statistical methods how the change in prevalence was assessed.

Fig 5: Provide a clear image

Fig 6: It seems this Fig 6 is comparing infection among different populations. Please indicate the age ranges being compared at each survey year 2008, 2014, and 2020.

Line 530: The methods section does not currently describe in sufficient detail how KAP survey was conducted. Include these details plus detailed findings on the KAP survey or consider removing these since the survey objectives do not currently mention the KAP survey.

Line 546: Provide as a supplementary table the results from the univariable regression model.

Describe in the results section the number of MDA rounds the country has received for both STH and SCH. Indicate the coverage achieved during each round among each age group.

Line 725: A key limitation that needs to be appreciated was that *S. haematobium* was not targeted using an appropriate diagnostic approach like urine filtration. Was this because of the cost of constraint?

Line 751: Following this survey, what is the frequency of delivering MDA to each of the surveyed age groups? Is this uniform for all the group?
