# Supplementary material for: Remapping parasite landscapes: Nationwide prevalence, intensity and risk factors of schistosomiasis and soil-transmitted helminthiasis in Rwanda
Source: PLoS Negl Trop Dis. 2025 Aug 25;19(8):e0013328. doi: 10.1371/journal.pntd.0013328 (PMC12377619; doi:10.1371/journal.pntd.0013328)
Supplement: Attachment — Submitted filename: Response to Reviewers_STH_Rwanda_paper_Final-25 May 2025.docx [file pntd.0013328.s010.docx]

**Response to reviewers PNTD-D-24-01920**

**Full title: Remapping parasite landscapes: a nationwide focused mapping of Schistosomiasis and Soil-Transmitted Helminthiasis and associated risk factors in Rwanda**

**Short title: Prevalence and risk Factors of schistosomiasis and helminthiasis in Rwanda**

**Authors:**

Ladislas Nshimiyimana**^1**^**, Jean Bosco Mbonigaba^1**^, Aimable Mbituyumuremyi**^1^**, Alison Ower**^2^**, Dieudonne Hakizimana**^3*^**, Elias Nyandwi**^4^**, Karen Palacio**^2^**, Alphonse Mutabazi**^1^**, Jeanne Uwizeyimana^5^, Leonard Uwayezu^6^, Michee Kabera**^1^**, Emmanuel Hakizimana**^1^**, Phocas Mazimpaka**^1^**, Emmanuel Ruzindana^7^, Eliah Shema**^4^**, Tharcisse Munyaneza^7^, Jean Bosco Mucaca^7^, Maurice Twahirwa^7^, Esperance Umumararungu^7^, Joseph Kagabo^8^, Richard Habimana^8^, Elias Niyituma**^1^**, Tonya Huston^5^, Jamie Tallant**^2^**, Warren Lancaster**^2^**, Nadine Rujeni**^8^**, Eugene Ruberanziza**^2^**

**Ladislas Nshimiyimana and Jean Bosco Mbonigaba contributed equally as the first authors

**Affiliations**

- **^1^**Division of Malaria and Other Parasitic Diseases, Rwanda Biomedical Centre, Ministry of Health, Kigali, Rwanda
- ^2^The END Fund, New York, USA
- ^3^Department of Global Health, University of Washington, Seattle, Washington, USA
- ^4^Centre for Geographic Information Systems and Remote Sensing (CGIS - UR), College of Sciences and Technology, University of Rwanda, Kigali, Rwanda
- ^5^Heart and Sole Africa, Kigali, Rwanda
- ^6^Byumba Level Two Teaching Hospital, Gicumbi, Rwanda
- ^7^National Reference Laboratory (NRL) Division, Rwanda Biomedical Center, Ministry of Health, Kigali, Rwanda
- ^8^School of Health Sciences, College of Medicine and Health Sciences, University of Rwanda, Kigali, Rwanda

***Corresponding author:** [**ddhakizimana@gmail.com**](mailto:ddhakizimana@gmail.com) **(DH);** [**ladi8n@gmail.com**](mailto:ladi8n@gmail.com) **(LN)**

**Key words:** Schistosomiasis, Soil-Transmitted Helminthiasis, survey, risk factors, parasite prevalence, Rwanda.

**Comments from Journal Requirements:**

1) Please ensure that the CRediT author contributions listed for every co-author are completed accurately and in full.
At this stage, the following Authors/Authors require contributions: Ladislas Nshimiyimana, Jean Bosco Mbonigaba, Aimable Mbituyumuremyi, Alison Ower, Dieudonne Hakizimana, , Elias Nyandwi, Karen Palacio, Alphonse Mutabazi, Jeanne Uwizeyimana, Leonard Uwayezu, Michee Kabera, Emmanuel Hakizimana, Phocas Mazimpaka, Emmanuel Ruzindana, Eliah Shema, Tharcisse Munyaneza, Jean Bosco Mucaca, Maurice Twahirwa, Esperance Umumararungu, Joseph Kagabo, Richard Habimana, Elias Niyituma, Tonya Huston, Jamie Tallant, Warren Lancaster, Nadine Rujeni, and Eugene Ruberanziza.

Please ensure that the full contributions of each author are acknowledged in the "Add/Edit/Remove Authors" section of our submission form.
The list of CRediT author contributions may be found here: <https://journals.plos.org/plosntds/s/authorship#loc-author-contributions>

Response: The full contributions of each author are acknowledged, aligned to the journal’s guideline. page 38, line: 833 to 856

2) Please ensure that the funders and grant numbers match between the Financial Disclosure field and the Funding Information tab in your submission form. Note that the funders must be provided in the same order in both places as well.
- State the initials, alongside each funding source, of each author to receive each grant. For example: "This work was supported by the National Institutes of Health (####### to AM; ###### to CJ) and the National Science Foundation (###### to AM)."
- State what role the funders took in the study. If the funders had no role in your study, please state: "The funders had no role in study design, data collection and analysis, decision to publish, or preparation of the manuscript.".
If you did not receive any funding for this study, please simply state: u201cThe authors received no specific funding for this work. u201d

**Response:** The funding section has been included and updated to mention that there was no specific grant number assigned. It now reads as follows on page 44, line: 1114

***Funding***

*This study was funded by The END Fund (*[*https://end.org/*](https://end.org/)*) through a grant to the Ministry of Health, Rwanda Biomedical Center – The Malaria, Neglected Tropical Diseases, and Other Parasitic Diseases Division (MOPDD). There was no specific grant number assigned. The study was independently implemented by the Rwanda Biomedical Center – MOPDD. The END Fund provided technical support in designing the study and reviewing the study results and manuscript but had no involvement in the data analysis.*

**Comments Reviewer #1:**

**Reviewer #1: This paper reports on a national scale helminth survey in Rwanda, following several years of treatment. It’s timely as it compares infection with earlier surveys in 2008 and 2020. The sample size is large and involved the collections of duplicate Kato – Katz slides and a large data set examining risk factors for infection. There are a few areas where it could be strengthened and / or additional information provided before publication.

Comments
My biggest questions are around study design: in particular the geographical level at which results are calculated, and the sampling approach**

**Geographical level of mapping / implementation. The WHO has moved towards recommending sub-district level mapping and implementation. In the Study design section (and similar in lines 699-723) the authors describe: ‘This study conducted a nationwide, community-based cross-sectional survey using a precision mapping approach to assess the prevalence and identify risk factors associated with SCH and STH.” And in lines 699-723 the authors talk about “A key strength of the study was the use of precision mapping, which provided a granular understanding of SCH, a focally distributed disease, resulting in more accurate prevalence estimates” and “The 2020 remapping exercise demonstrated how targeted sampling across diverse ecological settings can accurately identify transmission patterns at the community level”. But I only see results presented at the sector (district) level. Is that the level of implementation? To truly shrink the map then sub-district mapping and implementation is likely required. I do not think I consider the current approach as precision mapping, certainly not as most recently defined by WHO in the SPPA protocols. Was the mapping powered to give sub-district level results? If not, then I think much of section 699-723 needs amending.**

We appreciate the reviewer’s observation regarding the evolving definition of precision mapping as outlined in the WHO SPPA protocols. Our study employed a stratified, risk-informed sampling approach that involved ranking villages and purposively selecting areas considered to be at highest risk of schistosomiasis—an approach aligned with WHO’s SPPA recommendation to focus assessments in high-risk sites.

Specifically, we selected five sectors per district, and within each sector, two villages were chosen, resulting in 10 villages per district. While the village served as the unit of sampling and data collection, the sector was the primary sampling unit (PSU). Therefore, our survey was powered to produce **district-level** prevalence estimates rather than **sub-district** (e.g., sector or cell) estimates, as required by the most recent WHO definition. It is important to note that in Rwanda, the **cell** is the actual implementation unit for schistosomiasis control, which is at a lower administrative level than the sector.

Although we previously referred to our approach as “precision mapping” based on earlier interpretations, we acknowledge that it does not fully align with the updated WHO SPPA definition, which calls for four PSUs per sub-district and estimates powered at the sub-district level. Nevertheless, we believe our approach retains key strengths that are consistent with the core objectives of precision assessments. By working closely with local teams to identify and prioritize ecologically diverse and high-risk areas, our study generated data that more accurately reflect true SCH transmission patterns than previous traditional national mapping approaches, thereby supporting more tailored implementation planning.

This maintains the intent of our work—targeted, risk-informed sampling—without implying full alignment with the strict WHO SPPA definition. To avoid confusion, we have removed the section in the discussion that previously emphasized the importance of precision mapping and instead highlighted the use of focused mapping as a strength of the study. Additionally, we have noted the lack of sub-district-level estimates as a limitation. page 5, line: 167-171

**In Strengths and limitations**

***Page 37, line 795 -799 reads as*** *A key strength of the study was the use of focused mapping, which, while not fully aligned with the most recent WHO definition of precision mapping [1], emphasized targeting high-risk areas informed by ecological and local programmatic insights. This approach improved the accuracy of district-level estimates and ensured relevance for program planning by concentrating data collection where transmission was most likely.*

***And page 37, line 815-818 reads*** *as Finally, another key limitation is that the study was powered to provide estimates only at the district level, not at the sub-district (e.g., cell or sector) level, which limits its direct utility for micro-targeting of MDA interventions. Future research should adopt precision assessment approaches and employ sampling designs that generate sub-district-level estimates to better inform localized treatment decisions.*

**Sampling approach: In lines 294-295 the authors describe: “A three-stage sampling design was used to select study villages, focusing on areas likely to support schistosomiasis transmission due to their proximity to water bodies or wetlands.” (my emphasis). I think this is a reasonable programmatic approach when trying to (understandably) focus resources on the highest infection areas. But, by definition, it will lead to a biased estimate of sector / province / national level infection. And will inflate the current estimates and therefore underestimate any reductions since previous surveys (did they follow the same approach). I think this is worth the authors’ noting and discussing.**

We thank the reviewer for the insightful comment. Our purposive, risk-based sampling approach was designed to enhance programmatic relevance by targeting high-risk areas near water bodies. We acknowledge that this may lead to higher prevalence estimates at broader geographic levels, which could inadvertently undermine the perceived impact of past treatment efforts. For example, this may help explain why, in Fig 5, the SCH prevalence by Kato-Katz was 2.4% in our study (for SAC), slightly higher than the 1.9% reported in 2014. We have added this clarification to the Discussion to better contextualize the estimates and highlight their intended use in guiding targeted interventions.

The added paragraph is on page 35, line 716- 727 it reads as

*Additionally, we acknowledge that our purposive, risk-based sampling strategy—designed to identify high-transmission areas near water bodies—may have resulted in higher prevalence estimates than would be obtained through representative sampling. While this targeted approach enhances programmatic relevance by focusing on areas most likely to require intervention, it may also overestimate the true burden of disease at broader geographic levels and thereby underestimate the impact of previous treatment efforts. For example, the SCH prevalence based on KK in this survey was 2.4%, compared to 1.9% reported in the 2014 national mapping. However, this comparison should be interpreted with caution, as the 2014 survey employed school-based sampling with wider geographic coverage and did not specifically target high-risk areas, limiting direct comparability. Importantly, as Rwanda moves toward interruption of transmission, focusing on the highest-risk areas ensures that control efforts are directed where they are most needed, and provides a conservative upper bound for prevalence—offering assurance that transmission levels in lower-risk areas are likely to be even lower.*

**The authors describe MHI infections as rare. MHI in Ascaris lumbricoides was 8.1%. This seems quite substantial to me, and more than would be expected from an overall Ascaris prevalence of 27.0%.**

Thank you for this thoughtful observation. We have rechecked and validated our analysis. The 8.1% prevalence of moderate-to-heavy intensity (MHI) Ascaris lumbricoides infections is accurate and reflects the intensity distribution among individuals who tested positive by Kato-Katz. Among these positive cases, the mean eggs per gram (EPG) was 6,589 (SD = 13,471), with a range from 12 to 236,520. This wide distribution supports the presence of a notable proportion of moderate and heavy infections, despite an overall Ascaris prevalence of 27.0%. While heavy infections were uncommon, the majority of MHI cases were moderate. We agree that an 8.1% MHI prevalence is not negligible and have revised the manuscript to more accurately reflect this point. Specifically, the abstract now states (Page 2, Line 52-53):

*Moderate-to-heavy intensity (MHI) infections were observed in 8.1% for* A. lumbricoides*, 0.1% for hookworm, and 0.8% for* T. trichiura*.*

**How do the authors interpret the seeming conflicts in the SCH results: on one hand all sites had MHI < 1% so technically they have achieved elimination as a public health problem. But on the other hand, prevalence is still at 27% so continued MDA is required. Does this point to the need to update WHO guidance?**

Thank you for this thoughtful and important observation. The apparent discrepancy arises from differences in diagnostic methods and corresponding thresholds used for programmatic decision-making.

The national prevalence of Schistosoma mansoni at 27% is based on the Point-of-Care Circulating Cathodic Antigen (POC-CCA) test, where trace results are considered positive. In contrast, the mean intensity of heavy infections (MHI) of <1% is derived from the Kato-Katz (KK) method. The overall SCH prevalence measured by KK was 1.7%—specifically, 0.6% among 1–4-year-olds and 2.4% among 5–15-year-olds (Table 5). The MHI calculation, which forms the basis for determining elimination as a public health problem (EPHP), is based on KK data.

We would like to emphasize that EPHP targets, per current WHO guidelines, rely on the prevalence of **heavy-intensity infections using the Kato-Katz method**, not on total prevalence or results obtained from POC-CCA tests. Therefore, Rwanda’s 27% national prevalence by POC-CCA (with trace considered positive) should not be interpreted against the EPHP benchmark. Instead, it reflects a more sensitive measure aligned with Rwanda’s programmatic choice to err on the side of inclusivity in identifying infections.

Regarding the threshold for EPHP—defined as <1% prevalence of heavy-intensity infections in all implementation units (IUs)—our study was not powered to produce sub-IU level estimates. However, exploratory analysis in IUs (cells) where KK testing was conducted revealed that heavy-intensity infection prevalence ranged from 1.1% to 2.2%. This suggests that while Rwanda is close to meeting EPHP thresholds, a small number of areas may still exceed the target. The authors believe this gap could be closed in the upcoming reassessment, particularly as Rwanda continues to expand and refine its program.

Finally, it is important to clarify that achieving EPHP does not equate to stop mass drug administration (MDA). WHO guidance currently recommends **continuing MDA and strengthening surveillance** until transmission interruption is confirmed. In this context, Rwanda’s decision to consider trace-positive POC-CCA results reflects a strategic choice to increase diagnostic sensitivity and proactively address residual transmission, supported by adequate access to praziquantel for all at-risk populations.

In summary, our findings do not suggest the need to revise WHO guidance. Rather, they underscore the importance of aligning diagnostic strategies with national program goals and interpreting prevalence estimates carefully—especially when comparing across diagnostic tools designed for different use cases.

**There is such a significant difference between SCH prevalence calculated by KK (1.7%) and CCA (27.2%). Even with trace negative it’s a 6-fold difference. What is the authors’ interpretation of this?**

We thank the reviewer for raising this important point. The large discrepancy between Schistosoma mansoni prevalence as measured by Kato-Katz (KK) at 1.7% and by POC-CCA at 27.2% (including trace results) is consistent with findings from other studies in low-intensity settings [2,3]. This difference is primarily attributable to the limited sensitivity of KK, especially in detecting low-intensity infections, which become more common as transmission declines due to successful control interventions. In contrast, POC-CCA is known to be more sensitive in such contexts and can detect light or asymptomatic infections that KK often misses.

While the interpretation of trace results on POC-CCA remains debated, we chose to include them in our primary prevalence estimate for programmatic reasons. Rwanda's national strategy aims not only to eliminate schistosomiasis as a public health problem but also, where feasible, to interrupt transmission. Given that even light infections can sustain transmission, including trace results provides a more sensitive estimate and helps identify residual pockets of infection. This decision is supported by the availability of sufficient praziquantel donations, allowing the country to extend treatment to all identified risk areas and affected populations.

To support transparency and facilitate comparison, we also report alternative estimates that consider trace results as negative, aligning with more conservative interpretations.

In summary, the stark difference between KK and POC-CCA results underscores the limitations of relying on a single diagnostic approach—particularly KK—in low-endemicity settings. Our findings suggest that exclusive reliance on KK would likely underestimate the true burden of schistosomiasis and potentially delay elimination efforts. These points have been clarified in the revised discussion section (Page 35, Line 729-737).

*The POC-CCA test provided a more sensitive measure of* Schistosoma mansoni *prevalence in Rwanda than the KK method, particularly for detecting light and asymptomatic infections in a context of declining transmission due to ongoing mass drug administration. Including trace results raised prevalence to 27.2%, compared to 1.7% with KK, offering a more comprehensive picture of ongoing transmission risks. Although trace results remain debated [2,3], their inclusion aligns with Rwanda’s elimination goals, especially given the availability of praziquantel to support expanded treatment. These findings underscore the value of POC-CCA in low-endemicity settings while highlighting the importance of using both diagnostics during the transition toward elimination, given current WHO guidelines still rely on KK for assessing elimination as a public health problem.*

**The authors describe only 14 districts that had villages with >2% MHI? That seems very low when compared to overall MHI prevalence of Ascaris of 8.1%. Can it be checked?**

Thank you for the comment. We have rechecked and confirmed the results. The finding of 14 districts with villages exceeding 2% moderate-to-heavy intensity (MHI) infections is accurate and applies specifically to school-age children (SAC), which was the focus of this analysis. While the overall MHI prevalence for Ascaris lumbricoides is 8.1%, this is not evenly distributed geographically. The higher MHI burden is concentrated in districts in the north and west of the country, while other districts show considerably lower intensity levels. This uneven distribution explains why only about half of the districts exceed the 2% MHI threshold.

**The blood in urine data are not presented. Was there no S. haematobium found? Was any urine filtration done?**

Thank you for this observation. No Schistosoma haematobium infections were detected in this survey. As noted in the Methods section, on on page 7, line 207-208, haematuria was assessed using dipsticks, and when positive, urine samples were further examined via centrifugation and microscopy. All confirmed schistosomiasis cases were S. mansoni. Given the absence of S. haematobium, we did not include separate results on blood in urine in the main text. We have mentioned that on page 7, line 209 that *No Schistosoma haematobium was detected in any tests.*

**• The survey was conducted in 2020. How much treatment has there been since then?**

Thank you for the comments. The study was conducted after 12 years of MDs implementation, 24 MDA rounds and 12 rounds for SCH with effective MDA treatment coverage (>75%). It is read on page 34, line 658-661

**Abstract – The authors do not mention overall SCH prevalence, as they do with STH. I suggest adding.**
We thank the reviewer for this helpful suggestion. We have now added the SCH prevalence to the abstract. It reads: page 2, line 54-55

SCH prevalence was 1.7% (ranging from 0% to 38.3%) based on Kato-Katz testing, and 27.2% (ranging from 0% to 74.6%) when including POC-CCA trace positives.

**Abstract – The odds ratios reported here, are they univariate or multivariate? What’s the theory behind being single being a risk factor?**
We thank the reviewer for this important observation. The odds ratios reported in the abstract are from the **multivariate mixed-effects logistic regression model,** adjusted for other relevant covariates. We have clarified this in the abstract to avoid confusion.

Regarding the association between being single and increased odds of infection, this likely reflects socio-behavioral and occupational patterns. We have added sentence in the discussion section to reflect this. It reads: page 36, line 747-751

*The association between being single and increased odds of STH infection may reflect socio-behavioral and occupational patterns, as single individuals—particularly adult males—may be more engaged in farming or manual labor that increases exposure to contaminated soil. They may also be less likely to participate in household-based MDA or seek preventive care.*

**STH – Why do you think there is a drop in hookworm from 2008, but not in other STH species?**

Thank you for this insightful question. The marked decline in hookworm prevalence between 2008 and 2014—compared to the more modest reductions for Ascaris lumbricoides and Trichuris trichiura—is likely due to a combination of biological, programmatic, and behavioral factors. Hookworm eggs and larvae are more vulnerable to environmental conditions, making them more responsive to sanitation improvements. Albendazole, the drug used in mass drug administration (MDA), has greater efficacy against hookworm than Trichuris and moderate efficacy against Ascaris, likely contributing to the sharper decline.

Additionally, during this period, national hygiene promotion efforts included mandatory shoe-wearing campaigns in schools, which likely reduced children's exposure to infective hookworm larvae in contaminated soil. Reductions in open defecation and improvements in sanitation infrastructure further contributed to lowering transmission. However, hookworm remains more prevalent among adults, especially those involved in barefoot agricultural work. This highlights the importance of extending behavior change messaging beyond schools to target adult populations, promoting consistent footwear use and other protective behaviors.

We have added the following explanation to the Discussion section on page 35, line 706-714

*Our study observed a more pronounced decline in hookworm prevalence compared to Ascaris lumbricoides and Trichuris trichiura, likely attributable to albendazole’s higher efficacy against hookworm and concurrent behavioral and environmental improvements. National hygiene initiatives—such as mandatory shoe-wearing policies, particularly among school-aged children—and reductions in open defecation may have disproportionately disrupted hookworm transmission. Nevertheless, hookworm infection remains more prevalent among adults, likely due to continued exposure through barefoot agricultural activities. These findings underscore the need to expand behavior change interventions beyond school settings to effectively reach adult populations, with targeted messaging promoting consistent footwear use and other protective practices.*

**Line 614-615: “This stagnation suggests that high MDA coverage in SAC alone may be insufficient to interrupt transmission.” Agreed, this is not a surprise. The option of combining IVM with ALB is well noted and worthy of further study.**

We appreciate the reviewer’s agreement with our interpretation that high MDA coverage in school-aged children (SAC) alone may be insufficient to interrupt transmission. This reinforces the need to expand MDA strategies to include additional at-risk groups and complementary interventions. We also thank the reviewer for acknowledging the potential of combining ivermectin with albendazole. As noted, this option warrants further investigation, particularly in T. trichiura-endemic areas, and we have emphasized this in the discussion.

**Minor / editorial:**

**Lines 195-196, 425, 428 – species names should be in italics. Check throughout.
• Line 436 – repetition of ‘Additionally’.**

Thank you for pointing out these editorial issues. We have reviewed the manuscript and corrected all instances where species names (e.g., Ascaris lumbricoides, Trichuris trichiura) were not properly italicized. We have also removed the repeated use of “Additionally” on line 436 and made similar edits elsewhere to improve readability and flow.

**Comments Reviewer #2:**

**Methods:**

**Reviewer #2: Line 96 (Introduction): No description of the disease aside from the various worms causing the infection. Suggest providing at least a brief description of the mode of transmission and public health burden of SCH and STH, including e.g. the influence of sanitation, proximity to water bodies, any chronic conditions, risk of anaemia.**

We appreciate the reviewer’s suggestion. We have revised the introduction to briefly describe the transmission pathways and public health impact of STH and SCH, as well as the key environmental and behavioral risk factors. It reads on page 4, line 108-114:

*STH, caused by* Ascaris lumbricoides*,* Trichuris trichiura*, and hookworms, is transmitted through ingestion of eggs (in the case of* Ascaris *and* Trichuris*) or skin penetration by larvae (in the case of hookworms), often due to poor sanitation and hygiene. SCH, caused by* Schistosoma *spp., is acquired through skin contact with freshwater containing cercariae released by infected snails, particularly in areas near lakes, rivers, and wetlands [4]. Both infections contribute to substantial public health burden, particularly among school-aged children and agricultural communities. They are associated with anemia, malnutrition, stunting, cognitive impairment, and, in chronic SCH cases, hepatosplenic or urogenital complications [5,6].*

**Line 191-193: What do trace results mean in relation to POC-CCA results? Perhaps a brief description of the test would aid readers in understanding how the test is run and the utility (sensitivity, specificity etc.) of POC-CCA for urine testing, especially in the field, instead of standard diagnosis by microscopic examination/urine filtration (noted in lines 255-256 that additional urine tests were only done when haematuria was detected).**

**Also, how would the POC-CCA results be compared to the previous 2 rounds of mapping? How were the urine samples tested previously? Comparison of POC-CCA results between 2014 and 2020 assessments:**

**Based these 3 lines, the authors presented SCH status when either stool or urine tested positive i.e.
1. POC-CCA trace results were negative
2. KK or POC-CCA tests were positive but trace results were negative**

Thank you for this insightful comment. We have combined the response to this and the related comments above, as they address similar issues. We expanded the manuscript to include a brief description of the POC-CCA test, its procedure, and its utility in field settings, particularly its higher sensitivity compared to standard microscopy. The following text has been added to the Methods section (page 8 & 9, line 269-276):

*For SCH, stool samples were analyzed using the KK technique, while urine samples were tested using the POC-CCA test and dipsticks for haematuria. The POC-CCA is a rapid, urine-based assay that detects* Schistosoma mansoni *antigens and is well suited for field use due to its ease of administration and high sensitivity, particularly for low-intensity infections. One drop of urine was applied to the test cassette, followed by a drop of buffer solution; results were read after 20 minutes and scored as negative, trace, or positive (1+ to 3+), based on band intensity. In cases where haematuria was detected by dipstick, additional urine testing was conducted using centrifugation and microscopic examination to assess for* S. haematobium *infection antigens [2,3,7].*

Regarding the comparison of POC-CCA results between 2014 and 2020 assessments; In Rwanda, both the 2014 and 2020 assessments used the POC-CCA test for *S. mansoni*, but with different interpretations of trace results. In 2014, traces were considered negative, in line with WHO guidance at the time and to avoid overtreatment. However, follow-up analysis showed that ~50% of trace results were true positives (see reference 13). As a result, in 2020, trace results were reclassified as positive to improve sensitivity and detect residual transmission. This contributed to an increase in estimated SCH prevalence (27.2% in 2020 vs. 7.4% in 2014, excluding traces). This shift aligned with updated WHO guidelines, which recommend using trace-inclusive thresholds (<30%, 30–<75%, ≥75%) for MDA decision-making.

Earlier, in 2007/08, *S. haematobium* was assessed using urine reagent strips for micro-haematuria, followed by urine filtration only when haematuria was detected. No cases of *S. haematobium* were found, and the POC-CCA test was not used during that survey.

For the 2014 reassessment, the RBC/MOH used a commercially available POC-CCA cassette test (batch no. 33955; expiration date: June 2015; Rapid Medical Diagnostics, Pretoria, South Africa). The CCA antigen is a genus-specific glycan regurgitated by adult schistosome worms into the bloodstream and excreted in urine. The test procedure involved adding one drop of urine to the sample well of the cassette. After absorption, one drop of buffer solution (provided with the kit) was added, and results were read after 20 minutes. If the control band failed to appear, the test was deemed invalid and repeated with a new cassette. Valid tests were scored as negative or positive, with positive results further categorized as trace, 1+, 2+, or 3+ based on band intensity, following the manufacturer’s instructions. In addition, gross haematuria was assessed as a clinical indicator of S. haematobium infection. If present, urine samples were centrifuged at the nearest health facility to detect S. haematobium ova. As with the 2007/08 mapping, no cases were detected.

**Results:**

**Line 506: S. mansoni prevalence – is there a prevalence map by district available to illustrate this?**

Thank you for this suggestion. We chose not to include a district-level map for S. mansoni because schistosomiasis is a highly focal disease, with significant variation even within a single district. Mapping at the district level would imply uniform prevalence and risk across the district, potentially suggesting that the entire district population should be targeted for treatment. This would be misleading and inconsistent with Rwanda’s control strategy, which uses the **cell** (sub-sector) as the implementation unit for mass drug administration. Our precision mapping approach focused on high-risk villages to inform cell-level planning and accurately identify localized transmission hotspots. As such, we presented site-level prevalence maps (Fig 3) to better guide targeted intervention decisions.

We have added the following sentence to the Fig 3 caption:

“District-level mapping was not performed for S. mansoni as it would imply uniform prevalence and treatment eligibility across entire districts. Given the focal nature of transmission and the use of cells as the implementation unit in Rwanda, site-level data provide more actionable information for targeted intervention.” Page 21, line 511-516

**Line 547: The AORs were adjusted for what other variables, as this was not detailed in the methods?**

Thank you for this helpful comment. We have now clarified in the Methods section which variables were included in the adjusted models. Variables were selected based on bivariate significance (p < 0.05), prior literature, and expert input. We now explicitly list the covariates used in the multivariable logistic regression models for STH and SCH. Now it read on page 13, line 388-397

*Variables with p-values <0.05 in bivariate analyses and additional covariates identified from the literature were included in the multivariable mixed-effects logistic regression models with districts as random intercept to determine risk factors for STH and SCH in separate models. The STH model was adjusted for age category, marital status, education level, main occupation, toilet facility type, soap and water availability, main water source, water treatment behavior, use of human excreta as fertilizer, and proximity to marshlands (for rice and other uses). The SCH model was adjusted for age category, marital status, education level, main occupation, main water source, time to water source, use of human excreta as fertilizer, proximity to various water bodies (lake, river, pond/dam, marshlands, other), and walk time to the nearest water body.*

**Reviewer #2: Public health relevance, strengths and limitations, were clearly discussed.**

Thank you for your positive feedback. We appreciate your acknowledgment of the discussion around the study’s public health relevance, strengths, and limitations.

**Abstract, Lines 48 and 52 – SAC as the abbreviation for school-aged children will need to be written in full at first mention, same for AOR which I assume refers to adjusted odds ratio.**

Thank you for the suggestion. We have now written out **school-aged children (SAC)** and **adjusted odds ratio (AOR)** in full at their first mention in the abstract to ensure clarity for all readers.

**• Line 148 – “(500 mg) Samples were tested...” Suggest having the manuscript checked for punctuation/language.**
Thank you for pointing this out. We have reviewed and corrected the punctuation in this sentence, and have done an additional language check throughout the manuscript for clarity and consistency.

**• Line 157 – “Schistosomiasis” can already be abbreviated here.**
This has been updated. "Schistosomiasis" is now abbreviated as **SCH** at that point, following its first full mention.

It reads now Page 5, line 169-171*: Focused mapping focuses on sampling at a finer geographic scale, targeting more schools or villages within smaller administrative units to better capture the variability in SCH prevalence. Following recommended guidelines, SCH and STH were mapped together, ensuring a comprehensive assessment [8–11].*

**• Line 184–185 – “as positive” was mentioned twice in the same sentence.**
We appreciate your careful reading. The repetition has been removed, and the sentence has been revised for clarity. It reads Page 6, line 197:

For the main analysis, individuals were considered SCH-positive if they tested positive by either Kato-Katz (presence of at least one S. mansoni egg) or POC-CCA, with trace results included as positive.

**Figure 5: Text is not clear especially within the graph, a clearer figure will be useful. The captions in grey font below the x-axis “Estimates are absolute differences between surveys…” should also be in the figure caption (Line 523).**

Thank you for the helpful feedback. We have improved the clarity of Figure 5 by enhancing the resolution and increasing font size for all text elements. Additionally, we have moved the explanatory caption from within the figure to the formal figure caption, as suggested. Please note that while the figure embedded in the Word document may appear less clear due to formatting constraints, the figure submitted separately meets high-resolution standards and has passed PACE requirements in accordance with the journal’s submission guidelines.

**Line 195: “Any STH status indicates the presence of any STH infection, including Ascaris lumbricoides, hookworm, and Trichuris trichiura”. Scientific names were not italicised here.**

Thank you for catching this oversight. We have italicized all scientific names throughout the manuscript, including at Line 195, to ensure consistency with scientific writing standards.

Now it reads on page xxx line xx: Any STH status indicates the presence of any STH infection, including Ascaris lumbricoides, hookworm, and Trichuris trichiura.

**Summary and General Comments:**

**Reviewer #2: The authors presented a nationwide remapping survey as an update to the last two surveys in 2008 and 2014, with the inclusion of adults and sampling of schools/villages in smaller administrative units. Compared to previous surveys focused upon school-aged children, this is the first community-based survey assessing prevalence across three targeted age groups: pre-school age, school age and adults. The findings highlight the need to include adults in MDA programs and improving access to clean water and sanitation for more sustainable disease control, noting the limited effectiveness of albendazole against Trichuriasis. Results were clearly described with maps to illustrate the data, however some methods require clarification.**

We sincerely thank the reviewer for the thoughtful summary and encouraging feedback regarding the significance of our nationwide remapping survey, including its expanded scope to adults and community-level sampling. We appreciate the recognition of the value this provides for future MDA program design and sustainable control efforts.

In response to your note that some methodological aspects require clarification, we have revised the Methods section to provide greater detail. Specifically, we have clarified the criteria for selecting covariates in multivariable models, the structure of the sampling design across administrative units, and the definition and interpretation of trace results in POC-CCA testing. We trust these revisions improve the transparency and reproducibility of our approach.

**Comments Reviewer #3:**

**Remapping Parasite Landscapes: A Nationwide Precision Mapping Of Schistosomiasis and Soil-Transmitted Helminthiasis and Associated Risk Factors in Rwanda**

This STH/SCH remapping paper provides a good overview of the burden of these parasites in Rwanda. The paper explains why it is necessary to expand MDA to include the adult population. I have the following general, minor, and major comments for the authors. Detailed comments are embedded within the manuscript.pdf file.

**General Comments**

**The findings section of the abstract is long and needs to be summarized into key findings only. Summarize these paragraphs of risk factors into one or two sentences (from PDF).**

Thank you for the suggestion. While we recognize the importance of brevity, other reviewers specifically requested that we provide more detail in the abstract. Summarizing the risk factors into one or two sentences would limit important context for readers who rely on the abstract for a comprehensive overview and may not review the full text. We therefore chose to retain the key details to ensure clarity and accessibility of the findings.

**The manuscript needs proofreading to identify and correct sentence construction and grammatical errors**

**All comments in track changes are given in the pdf document.**

Thank you for this valuable feedback. We have revised the Findings section of the abstract to improve clarity and focus on key results. While we have shortened it where possible, we also received suggestions from other reviewers to include additional information in the abstract. As a result, the abstract may still appear relatively long, but we have ensured it remains concise and contains only essential information aligned with journal requirements.

Additionally, we have thoroughly proofread the manuscript to correct sentence construction and grammatical issues. We also reviewed and responded to all comments provided in the PDF version with tracked changes and made the necessary edits and clarifications accordingly.

Regarding the comments in track changes provided in the PDF document, please note that comments from both the Word document and the PDF were reviewed and merged where they addressed similar issues. This was done to provide a consolidated explanation in one place and to avoid repetition.

**Minor Comments**

Lines 151-152:*: Those tested positive for schistosomiasis151 aged five years and above received a single dose of Praziquantel tablet 600mg while those aged below five years were treated at the health center following the existing treatment guidelines.*

*Question:* what does the existing national treatment guideline say on SCH treatment for individuals below 5 years?

Under 5 years old participants tested positive to SCH were treated according to national guideline that consider mg/kg to determine needed dose.

**Line 185: Indicate which species of SCH was tested using POC-CCA**

**Lines 189-190: This is a classification of *S. mansoni*; how was the intensity of *S. haematobium* classified?**

Thank you for your comment. We have clarified in the Methods section that the POC-CCA test was used specifically to detect Schistosoma mansoni. As no S. haematobium ova were detected in examined urine samples, infection intensity classification for S. haematobium was not applicable and was therefore not included. The following text was included on page 7, line 207-210:

Schistosoma haematobium infection was assessed through gross haematuria and dipstick testing. In cases with haematuria, urine samples were centrifuged and examined microscopically for S. haematobium ova. As no ova were detected and haematuria was not a primary focus of this study, infection intensity classification for S. haematobium was not applicable and was therefore not included.

**Write the species' scientific names in italics throughout the text**

Thank you for the observation. We have carefully reviewed the manuscript and ensured that all scientific names of species are now consistently italicized throughout the text, in accordance with journal guidelines.

**Line 210: Why was the questionnaire administered to individuals aged 10 years and above only? Couldn’t this be administered to children below 10 years as well through their guardians?**

Thank you for the question. Variables such as marital status, education level, occupation, as well as knowledge and attitude indicators were collected starting from age 10. This is because children aged 10 and above are typically able to comprehend and respond meaningfully to such questions. In addition, this age group is often engaged in formal education. This age threshold aligns with national survey practices and ensures the accuracy and relevance of the responses.

Page 9 line *266* tests*. Results were compared, and discrepancies over 10% but less than 20% were discussed to reach consensus. Larger discrepancies (over 20%) triggered external review and refresher training.*

*Question:* From the QC, how many samples required consensus and external review?

Thank you for the question, for microscopy: During field activities, two laboratory technicians read duplicate slides (one read A another read B). A qualified quality controller (a senior technician from NRL or referral hospital) in each team conducted quality control of 20% randomly selected slides (+/- 12 slides). This section was revised it is read on page 9, line 277-285.

Line 282: Where was the server located? How was compliance with local data protection laws ensured? Was lab data directly entered into ODK system?

Thank you for these important questions. Laboratory data we recorded on lab sheets and entered into entered ODK system on daily basis. The questionnaire had separate section for each participant with unique study ID, the lab results were added after the interview.

The central server used for storing the ODK data was hosted by the Rwanda Biomedical Centre (RBC), located within Rwanda. This ensured full compliance with national data protection regulations. Access to the data was restricted to authorized personnel, and all identifiers were removed prior to analysis. The study protocol, including data management and protection procedures, was reviewed and approved by the Rwanda National Ethics Committee.

To clarify, we have revised the sentence on page 8, line 258-261 to now read:

*All data were uploaded daily to a central server hosted by the Rwanda Biomedical Centre, following verification by the team leader for questionnaire data and by senior laboratory technicians for laboratory results. All procedures adhered to local data protection regulations.*

**Lines 349-350: Is the assumption that STH is homogeneous across districts supported by past studies? Include the references supporting this assumption.**

Thank you for the comment. We acknowledge the importance of this point. The assumption of homogeneity in STH distribution across districts was based on previous national mapping efforts, which—due to their limited spatial resolution—did not reveal consistent fine-scale heterogeneity. While this assumption may overlook local variability, it was a practical starting point informed by available data and the need for operational efficiency. We also recognize that applying a sampling strategy originally designed for SCH may have influenced the STH estimates, as some low-risk villages had zero probability of selection and were therefore excluded from the sampling frame. This limitation has been noted in the revised manuscript. Nevertheless, we believe the STH estimates remain valid and informative, given the large sample size and the wide geographic distribution of selected villages across all districts, which likely captured diverse environmental and transmission contexts relevant to STH. We have clarified these points in the manuscript and added references to previous national surveys

**See page 13, lines 370–375: Consideration for STH:** It was assumed that STH transmission is relatively homogeneous within districts or subdistricts, based on previous national mapping efforts that did not reveal clear or consistent variation at finer geographic scales [12–15]. Consequently, the villages and participants selected for SCH were also considered suitable for assessing STH. This approach was deemed appropriate given the large sample size, broad geographic coverage of the selected villages, and the need for operational efficiency. Therefore, no additional sampling was conducted specifically for STH.

**Page 37, lines 805–807 in limitations:** The use of a sampling strategy originally designed for SCH may have influenced STH estimates, as some low-risk villages were not included in the sampling frame; however, this limitation was likely mitigated by the large sample size and the broad geographic distribution of selected villages.

**Table 5: Report test statistic results as: test-statistic used, test statistic result and p-value (e.g., X^2^ = 12.435, p<0.001).**

Thank you for the comment. We have added the test statistics along with their global p-values to the univariate results table in the appendix, rather than Table 5, to avoid overcrowding the main table. We believe this keeps Table 5 clear and focused, while still providing complete statistical details in the supplementary materials. This is mention on page 23, line 550-551:

*Univariate regression results, including odds ratios, 95% confidence intervals, and Chi-square test statistics, are presented in S4 Appendix.*

**Lines 506-507: Were the previous *S. mansoni* surveys (2008, 2014, and 2020) assessed using CCA as well? Provide citations to those previous surveys. It is important to compare infection results diagnosed using similar diagnostic technique**

Thank you for the comment. We have updated the Methods section and added the appropriate reference for the survey. Unfortunately, the 2008 survey was not published, so we referenced the program report. Specifically, the POC-CCA test was used in the 2014 and 2020 surveys. We have also included the age range for each survey year. This revision can be found on page 14, lines 403–415.

***To assess changes in prevalence across surveys, we focused on SAC, as previous surveys were conducted in this age group: 10–16 years in schools in 2008, 9–18 years in schools in 2014, and 5–15 years in the community in 2020. Historical prevalence data and the number of individuals tested for each parasite species in 2008 and 2014 were obtained from national program survey reports and related publications [12,13]………For*** S. mansoni***, results from KK and POC-CCA tests were presented separately. For the POC-CCA, trace results were considered positive in both the 2014 and 2020 datasets; the 2008 survey did not include the POC-CCA test.*** *No* S. haematobium *infections were detected in any of the survey years.* ***All results are reported as percentage point differences. Details of the estimates from previous surveys for each STH and SCH are available in S5 Appendix.***

**Page 13, line 373 - Include the actual number of participants as well**

Thank you for the comment. We have revised the text to include the actual number of participants alongside the percentages. It now reads as (page 15, line 440-444 )

*The study included 17,765 participants across five provinces: East (n = 4,115; 23.2%), Kigali City (n = 1,768; 10.0%), North (n = 2,981; 16.8%), South (n = 4,750; 26.7%), and West (n = 4,151; 23.4%). The sample comprised 9,582 (53.9%) females and 8,183 (46.1%) males. Participants were categorized as preschool-aged children (1–4 years; n = 5,573, 31.4%), school-aged children (5–15 years; n = 6,166, 34.7%), and adults (≥16 years; n = 6,026, 33.9%).*

**Line 550-563, and 565-575: Describe all risk factors first, then protective factors.**

Thank you for the comment. We have revised the Results section to clearly separate and present risk factors before protective factors, as requested. This structure has been applied consistently for both STH and SCH results to enhance clarity and alignment with reporting standards. On page 31, line 602-629)

*Table 7 shows the results from mixed effect logistic regression models identifying factors associated with STH and S. mansoni (SCH) infections, adjusted for other variables and accounting for variations among districts.*

***Several sociodemographic, environmental, and behavioral factors were associated with STH infection. Individuals who were single had significantly higher odds of STH infection compared to those who were married (AOR: 1.74, 95% CI: 1.32–2.28, p < 0.001). Those with no formal education were more likely to be infected compared to individuals with primary education (AOR: 1.56, 95% CI: 1.37–1.78, p < 0.001). Use of treated human excreta as fertilizer was associated with increased odds of STH infection (AOR: 1.43, 95% CI: 1.22–1.66, p < 0.001), as was the use of non-improved water sources (AOR: 1.17, 95% CI: 1.02–1.34, p = 0.024). Additionally, residing near marshlands used for other activities was linked to higher odds of infection (AOR: 1.17, 95% CI: 1.02–1.35, p = 0.025).***

***In contrast, several protective factors were identified. Individuals with secondary or higher education had significantly lower odds of infection compared to those with primary education (AOR: 0.55, 95% CI: 0.37–0.81, p = 0.003). Unemployed individuals (AOR: 0.34, 95% CI: 0.23–0.51, p < 0.001) and those who were self-employed or retired (AOR: 0.53, 95% CI: 0.31–0.91, p = 0.020) had lower odds of infection compared to those working in agriculture or farming. Households with toilet facilities deeper than three meters had reduced odds of STH infection (AOR: 0.78, 95% CI: 0.64–0.96, p = 0.018), and consistent treatment of drinking water was also associated with a protective effect (AOR: 0.79, 95% CI: 0.68–0.92, p = 0.003).***

*For SCH,* ***single individuals were more likely to be infected compared to those who were married (AOR: 1.61, 95% CI: 1.11–2.33, p = 0.011). Participants with no formal education had higher odds of infection than those with primary education (AOR: 1.41, 95% CI: 1.16–1.72, p = 0.001). Living more than one hour from a water source was associated with increased odds of SCH infection compared to living 30–60 minutes away (AOR: 1.42, 95% CI: 1.06–1.91, p = 0.019). Proximity to lakes was also a significant risk factor (AOR: 1.76, 95% CI: 1.37–2.27, p < 0.001).***

***Use of human excreta as manure—whether untreated (AOR: 1.60, 95% CI: 1.06–2.41, p = 0.027) or treated prior to use (AOR: 1.32, 95% CI: 1.06–1.65, p = 0.014)—was linked to higher odds of SCH infection. Similarly, residing near marshlands used for rice farming was associated with increased infection risk (AOR: 1.31, 95% CI: 1.00–1.72, p = 0.047).***

**Line 702: I have not seen how this mapping has targeted ecological or environmental factors, but only geographical boundaries.**

Thank you for the comment. We appreciate the opportunity to clarify. In addition to geographical boundaries, our mapping approach incorporated several ecological and environmental factors known to influence schistosomiasis risk. These included proximity to water bodies, type of water sources (e.g., lakes, rivers, wetlands), water-related human activities (e.g., fishing, rice cultivation, swimming), estimated population exposure levels, and the presence of confirmed cases. These factors were used to stratify villages and assess ecological suitability for transmission, as detailed in the Methods section and summarized in Table 1. However, please note that the text related to this has been removed from the manuscript based on suggestions from another reviewer, so the current comment may no longer be applicable to the revised version.

**Line 982: In line with open science, properly de-identified and anonymized datasets need to be deposited in an open, freely accessible public repository.**

Thank you for the comment. While we support the principles of open science, the participants' consent did not include permission for public data sharing, and current national regulations do not permit public release of national datasets. However, the data can be made available upon reasonable request including needed further data analysis. Interested researchers may contact the Ministry of Health through the Rwanda Biomedical Center, providing a brief explanation of the intended use. The contact email for data requests has been included in the manuscript.

**Major Comments**

**Lines 155-157: Clearly describe what precision mapping entails. Any reference on the methodology of precision mapping? Include the dates when the survey was carried out.**

Thank you for the comment. We have now added a clearer description of precision mapping in the Methods section, along with a reference to support the methodology. The document reference is available here (also referenced in the text): <https://www.ntdtoolbox.org/toolbox-search/schistosomiasis-practical-precision-assessment-sppa>

Data collection was carried out from 13 November to 24 December 2020 it took place 5 months after MDA (Page 8, line 236). It is added in the main manuscripts. The tools and the study procedures were piloted and validated before real data collection. Questionnaire and lab forms are added to the supplementary materials.

**Line 181: Was *S. haematobium* assessed in this study? Which diagnostic technique was used to assess it?**

Thank you for your comment. We have clarified in the Methods section that Schistosoma haematobium infection was assessed through gross haematuria and dipstick testing. In cases with haematuria, urine samples were centrifuged and examined microscopically for S. haematobium ova. As no S. haematobium ova were detected in examined urine samples, infection intensity classification for S. haematobium was not applicable and was therefore not included. The following text was included on page 7, line 207-210

Schistosoma haematobium infection was assessed through gross haematuria and dipstick testing. In cases with haematuria, urine samples were centrifuged and examined microscopically for S. haematobium ova. As no ova were detected and haematuria was not a primary focus of this study, infection intensity classification for S. haematobium was not applicable and was therefore not included.

**Line 359: Indicate which statistical model was used to calculate the infection prevalence.**

**Clearly describe in the statistical methods how infection intensity was modelled.**

Thank you for the comment. We have updated the Statistical Methods section to clearly describe how infection intensity was modeled. Specifically, infection intensity for STH was categorized based on WHO thresholds (light, moderate, heavy) using eggs per gram (EPG) values, and summarized using frequencies and percentages. For Schistosoma mansoni, intensity classification was also based on EPG as per WHO guidelines. No modeling was performed beyond descriptive categorization, as the focus was on prevalence and intensity distribution across groups. In addition, we generated age-weighted estimates using the R “survey” package to account for the difference in age distribution. These the methods and results of the age-adjusted analysis were presented in S1 Appendix for reference. The following section on page 13, line 381-386 was updated in the method section

*Descriptive statistics were used to summarize the data. Frequencies and percentages were calculated to describe the study population and the prevalence of SCH and STH infections overall and by species. Infection intensity for SCH and STH was categorized as light, moderate, or heavy according to WHO guidelines, based on EPG values [16,17],. Additionally, a sensitivity analysis was conducted to adjust prevalence estimates for age; the methods and results of the age-adjusted analysis are presented in S1 Appendix.*

**For in mixed-effects logistic regression models, was this a univariable or multivariable regression model?**

Thank you for the comment. The mixed-effects logistic regression models were multivariable. This has been clarified in the Statistical Methods section. As described, variables with p-values <0.05 in bivariate analyses and additional covariates identified from the literature were included in the final models, with districts specified as random intercepts. We have ensured this is clearly stated in the manuscript on page 13 & 14, line 388-391. It reads as:

*Variables with p-values <0.05 in bivariate analyses and additional covariates identified from the literature were included in the multivariable mixed-effects logistic regression models with districts as random intercept to determine risk factors for STH and SCH in separate models.*

**Figs 2, 3 and 4: When plotting the point estimates and district prevalence on the map, use prevalence classification categories that align with WHO classification. This will aid programmatic decisions and comparability. See the M&E framework (Assessing schistosomiasis and soil-transmitted helminthiases control programmes: monitoring and evaluation framework. Geneva: World Health Organization; 2024. Licence: CC BY-NC-SA 3.0 IGO.).**

Thank you for your thoughtful comment and for directing us to the updated WHO M&E framework for schistosomiasis and soil-transmitted helminthiasis (STH). We appreciate the importance of aligning prevalence classifications with current WHO thresholds to enhance programmatic utility and comparability. The prevalence categories used in our maps were chosen to reflect both historical WHO guidance available at the time of the survey and the classifications applied in previous national surveys in Rwanda. As outlined in the 2024 WHO M&E framework (Annex 2, page 49), STH prevalence of 10–<20% warrants annual preventive chemotherapy (PC), and ≥20% requires biannual PC. In our data (see S2 Appendix), no districts had prevalence <2%, and eight districts fell within the 10–<20% range [17]. Rather than grouping all areas ≥20% together, we further divided this into 20–49% and ≥50% to highlight districts with a persistently high burden that would otherwise be masked. This approach is consistent with previous WHO classifications (e.g., 2011 guidelines)[16], which were in use at the time of our survey and recommended annual, biannual, or even triannual PC based on detailed thresholds (10–<20%, 20–<50%, and ≥50%). These same categories were applied in the 2008 and 2014 national surveys, and using them here ensures comparability across time in Fig 6, especially given that the earlier data were only available in aggregated format **[12,13]**.

In Fig 2, we further disaggregated site-level STH prevalence into <10%, 10–19%, 20–29%, 30–49%, and ≥50% to show geographic heterogeneity at a finer scale. This level of detail is especially valuable for program managers seeking to identify localized hotspots and understand which STH species (e.g., Ascaris lumbricoides) are driving the burden in specific areas. Importantly, those who prefer to apply the current WHO categories of 2 to <10%, ≥10% to <20%, and ≥20% can still do so using our data, but the disaggregated bands provide additional insights for decision-making.

Similarly, for Schistosoma mansoni (Figure 3), we used categories aligned with the WHO guidance in effect at the time of the survey, which recommended PC every two years for prevalence 1–<10%, maintaining PC frequency for 10–<50%, and twice-yearly PC for prevalence ≥50%. These thresholds were operationally relevant and used to guide program decisions in Rwanda following the 2020 mapping.

While we acknowledge the value of adopting the most recent WHO framework, we believe that the use of more detailed and historically consistent thresholds in our figures provides a richer and more actionable presentation of the data. This approach allows both national programs and external partners to assess changes over time, understand local variation, and align strategies with either past or current guidance. In future mapping efforts, including the upcoming national impact assessment, we plan to apply the updated WHO categories. For the purposes of this publication, however, we respectfully propose to retain the current prevalence categories and map structure, as they offer greater detail without compromising interpretability or comparability.

**Lines 500-502: Clearly describe in the statistical methods how the change in prevalence was assessed.**

Thank you for the comment. We have updated the Statistical Methods section to clearly describe how changes in prevalence were assessed. Specifically, we calculated absolute differences in prevalence between survey years and estimated 95% confidence intervals using the standard error of the difference between two independent proportions, applying the standard normal approximation (Wald method). We have also clarified when CCA was used. This revision can be found on page 14, lines 403–415.

***To assess changes in prevalence across surveys, we focused on SAC, as previous surveys were conducted in this age group: 10–16 years in schools in 2008, 9–18 years in schools in 2014, and 5–15 years in the community in 2020. Historical prevalence data and the number of individuals tested for each parasite species in 2008 and 2014 were obtained from national program survey reports and related publications [12,13]. For each infection, absolute differences in prevalence between survey years (2008–2014 and 2014–2020) were calculated, along with 95% confidence intervals (CIs). CIs were estimated using the standard error of the difference between two survey proportions, based on the standard normal approximation (Wald method), which assumes independent samples and sufficiently large sample sizes. For*** S. mansoni***, results from KK and POC-CCA tests were presented separately. For the POC-CCA, trace results were considered positive in both the 2014 and 2020 datasets; the 2008 survey did not include the POC-CCA test.*** *No* S. haematobium *infections were detected in any of the survey years.* ***All results are reported as percentage point differences. Details of the estimates from previous surveys for each STH and SCH are available in S5 Appendix.***

**Fig 5: Provide a clear image**

Thank you for the helpful feedback. We have improved the clarity of Figure 5 by enhancing the resolution and increasing font size for all text elements. Please note that while the figure embedded in the Word document may appear less clear due to formatting constraints, the figure submitted separately meets high-resolution standards and has passed PACE requirements in accordance with the journal’s submission guidelines.

**Fig 6: It seems this Fig 6 is comparing infection among different populations. Please indicate the age ranges being compared at each survey year 2008, 2014, and 2020.**

Thank you for the comment. We have updated the figure legend for Fig 6 to clarify the age ranges represented in each survey year. Specifically, the 2008 survey included SAC aged 10–16 years, the 2014 survey included SAC aged 9–18 years, and the 2020 survey included SAC aged 5–15 years.

**Line 530: The methods section does not currently describe in sufficient detail how KAP survey was conducted. Include these details plus detailed findings on the KAP survey or consider removing these since the survey objectives do not currently mention the KAP survey.**

Thank you for the comment. While the KAP survey was not a core objective of the study, we believe it adds important contextual information that enhances the interpretation of the main findings, particularly in relation to community practices and awareness that may influence STH and SCH transmission. We have now expanded the Methods section to clearly describe how the KAP survey was conducted and included a summary of key findings. This ensures consistency with the overall aims of informing control strategies and supports the value of retaining this component in the manuscript. The sections added is on Page 14 & 15, line 419-437 and readds as

***Knowledge and attitudes assessment***

*A structured knowledge and attitudes module was included to assess community awareness and perceptions of SCH and STH among participants aged 10 years and above, who were able to respond to knowledge-related questions. Participants were first asked if they had heard of either condition. Those who responded “yes” were asked about sources of information (e.g., churches, community health workers, health facilities, media, schools, parents/elders, or other), time since last receiving information, knowledge of transmission, and awareness of treatment.*

*Knowledge of transmission was defined as correctly identifying key pathways—contact with contaminated water for SCH, and poor hygiene, open defecation, or unsafe food or water for STH. Treatment awareness was based on identifying albendazole, mebendazole, or similar deworming medications.*

*Attitudes were assessed using Likert-scale questions. Responses of “agree” or “strongly agree” were coded as “Agree,” while all others were classified as “Disagree.” For SCH, attitude items covered perceived severity, importance of screening, treatment uptake, and care-seeking for symptoms. For STH, they included beliefs about preventability, use of untreated human excreta as fertilizer, and traditional medicine.*

*Responses were stratified by age group—school-aged children (SAC: 10–15 years) and adults (≥16 years)—and summarized as frequencies and percentages. “No” responses and missing data were excluded from the denominator for each indicator.*

**Line 546: Provide as a supplementary table the results from the univariable regression model.**

Thank you for the comment. We have provided the full results from the univariable regression models for both **Any STH** and **S. mansoni (KK or CCA positive)** as a supplementary table (see Supplementary Table S5). The table includes odds ratios (ORs), 95% confidence intervals, test statistics, and p-values for each variable. This is mention on page 23, line 550-551:

*Univariate regression results, including odds ratios, 95% confidence intervals, and Chi-square test statistics, are presented in S4 Appendix.*

Describe in the results section the number of MDA rounds the country has received for both STH and SCH. Indicate the coverage achieved during each round among each age group.

Thank you for the comments. The study was conducted after 12 years of MDs implementation, 24 MDA rounds and 12 rounds for SCH with WHO recommended effective MDA treatment coverage (>75%). The paragraph was revised from page 34, line 658-661

**Line 725: A key limitation that needs to be appreciated was that *S. haematobium* was not targeted using an appropriate diagnostic approach like urine filtration. Was this because of the cost of constraint?**

Thank you for the comment. As noted in the Methods section on Page XX line xxx and earlier in responses, S. haematobium infection was assessed through clinical indicators, including gross haematuria and dipstick testing. In cases with haematuria, urine samples were centrifuged and examined microscopically; however, no S. haematobium ova were detected in examined urine samples, infection intensity classification for S. haematobium was not applicable and was therefore not included in this paper.

Line 751: Following this survey, what is the frequency of delivering MDA to each of the surveyed age groups? Is this uniform for all the group?

Thank you for the comment. The MDA strategy in Rwanda, for STH the MDA is two rounds per year using Albendazole or Mebendazole for all targeted age group. For SCH it is one MDA round annually for all targeted age groups.

**References**

1.	World Health Organization (WHO). Practical and Precision Assessments for Schistosomiasis - A manual for impact assessments, Version 1 [Internet]. Geneva: World Health Organization (WHO); 2025. Available from: https://www.ntdtoolbox.org/toolbox-search/schistosomiasis-practical-precision-assessment-sppa

2.	Colley DG, King CH, Kittur N, Ramzy RMR, Secor WE, Fredericks-James M, et al. Evaluation, Validation, and Recognition of the Point-of-Care Circulating Cathodic  Antigen, Urine-Based Assay for Mapping Schistosoma mansoni Infections. Am J Trop Med Hyg. 2020 Jul;103(1_Suppl):42–9.

3.	Graeff-Teixeira C, Favero V, Pascoal VF, de Souza RP, Rigo F de V, Agnese LHD, et al. Low specificity of point-of-care circulating cathodic antigen (POCCCA) diagnostic test in a non-endemic area for schistosomiasis mansoni in Brazil. Acta Trop [Internet]. 2021;217:105863. Available from: https://www.sciencedirect.com/science/article/pii/S0001706X21000425

4.	Bethony J, Brooker S, Albonico M, Geiger SM, Loukas A, Diemert D, et al. Soil-transmitted helminth infections: ascariasis, trichuriasis, and hookworm. Lancet. 2006 May;367(9521):1521–32.

5.	Gasparinho C, Gonçalves MH, Chissaque A, Silva GL, Fortes F, Gonçalves L. Wasting, Stunting, and Anemia in Angolan Children after Deworming with Albendazole or a Test-and-Treat Approach for Intestinal Parasites: Binary Longitudinal Models with Temporal Structure in a Four-Arm Randomized Trial. Nutrients. 2022;14(11).

6.	Taylor-Robinson DC, Maayan N, Soares-Weiser K, Donegan S, Garner P. Deworming drugs for soil-transmitted intestinal worms in children: effects on nutritional indicators, haemoglobin and school performance. Cochrane Database of Systematic Reviews. 2012 Jul 11;

7.	Tchuem Tchuenté LA, Kueté Fouodo CJ, Kamwa Ngassam RI, Sumo L, Dongmo Noumedem C, Kenfack CM, et al. Evaluation of circulating cathodic antigen (CCA) urine-tests for diagnosis of  Schistosoma mansoni infection in Cameroon. PLoS Negl Trop Dis. 2012;6(7):e1758.

8.	Baker MC, Mathieu E, Fleming FM, Deming M, King JD, Garba A, et al. Mapping, monitoring, and surveillance of neglected tropical diseases: towards a policy framework. The Lancet [Internet]. 2010 Jan 16;375(9710):231–8. Available from: https://doi.org/10.1016/S0140-6736(09)61458-6

9.	Tchuem Tchuenté LA, Stothard JR, Rollinson D, Reinhard-Rupp J. Precision mapping: An innovative tool and way forward to shrink the map, better  target interventions, and accelerate toward the elimination of schistosomiasis. PLoS Negl Trop Dis. 2018 Aug;12(8):e0006563.

10.	World Health Organization (WHO). Assessing schistosomiasis and soil-transmitted helminthiases control programmes Monitoring and evaluation framework. Geneva: World Health Organization; 2024.

11.	Kepha S, Ochol D, Wakesho F, Omondi W, Njenga SM, Njaanake K, et al. Precision mapping of schistosomiasis and soil-transmitted helminthiasis among  school age children at the coastal region, Kenya. PLoS Negl Trop Dis. 2023 Jan;17(1):e0011043.

12.	The END Fund and Rwanda Biomedical Centre. Rwanda’s progress towards controlling, interrupting transmission and eliminating Schistosomiasis and Soil-Transmitted Helminthiases: A review of program achievements, 2008 to 2020, and the revised treatment strategy and goals for 2024. Kigali- Rwanda; 2024.

13.	Ruberanziza E, Owada K, Clark NJ, Umulisa I, Ortu G, Lancaster W, et al. Mapping Soil-Transmitted Helminth Parasite Infection in Rwanda: Estimating  Endemicity and Identifying At-Risk Populations. Trop Med Infect Dis. 2019 Jun;4(2).

14.	Rujeni N, Morona D, Ruberanziza E, Mazigo HD. Schistosomiasis and soil-transmitted helminthiasis in Rwanda: an update on their  epidemiology and control. Infect Dis Poverty. 2017 Mar;6(1):8.

15.	Kabatende J, Mugisha M, Ntirenganya L, Barry A, Ruberanziza E, Mbonigaba JB, et al. Prevalence, Intensity, and Correlates of Soil-Transmitted Helminth Infections  among School Children after a Decade of Preventive Chemotherapy in Western Rwanda. Pathogens. 2020 Dec;9(12).

16.	World Health Organization (WHO). Helminth control in school-age children: a guide for managers of control programmes. 2nd edition [Internet]. 2011 [cited 2022 Sep 9]. Available from: https://www.who.int/publications/i/item/9789241548267

17.	World Health Organization (WHO). Assessing schistosomiasis and soil-transmitted helminthiases control programmes Monitoring and evaluation framework [Internet]. Geneva: World Health Organization: Licence: CC BY-NC-SA 3.0 IGO.; 2024. Available from: https://iris.who.int/bitstream/handle/10665/379352/9789240099364-eng.pdf?sequence=1&isAllowed=y
